# Supplementary material for: Chiral recognition of neutral guests by chiral naphthotubes with a bis-thiourea endo-functionalized cavity
Source: Nat Commun. 2023 Sep 13;14:5645. doi: 10.1038/s41467-023-41390-8 (PMC10499783; doi:10.1038/s41467-023-41390-8)
Supplement: Supplementary file 1 — Supplementary Information [file 41467_2023_41390_MOESM1_ESM.pdf]

Supplementary Information

Chiral Recognition of Neutral Guests by Chiral Naphthotubes with a Bis-thiourea Endo-Functionalized Cavity

Song-Meng Wang,[†1] Yan-Fang Wang,[†1] Liping Huang,[1] Li-Shuo Zheng,[1] Hao Nian,[1] Yu-Tao Zheng,[1] Huan Yao,[2] Wei Jiang[1]*, Xiaoping Wang[1]*, Liu-Pan Yang[2]*

[1] Department of Chemistry, Southern University of Science and Technology, Xueyuan Blvd 1088, Shenzhen, 518055, China. [2] School of Pharmaceutical Science, Hengyang Medical School, University of South China, Hengyang, Hunan, 421001, China.

*Corresponding author E-mail: jiangw@sustech.edu.cn, wangxp@sustech.edu.cn, yanglp@usc.edu.cn

[†] Song-Meng Wang, Yan-Fang Wang contributed equally to this work.

Table of Contents

1 Supplementary Methods 2

1.1 General Method 2

2 Supplementary Discussion

2.1 Synthetic Procedures of Chiral Naphthotubes 3

2.2 2D NMR spectra of Chiral Naphthotubes 17

2.3 Single Crystals of Chiral Naphthotubes (anti-isomers) 21

2.4 Dihedral Angles of bis-Naphthalene Clefts in Naphthotubes 23

2.5 HPLC of Chiral naphthotubes on Chiral Column 24

2.6 ^{1}H NMR Spectra of the 1: 1 Host-Guest Complexes 25

2.7 ^{1}H NMR Spectra of the 1: 1 Host-Guest Complexes in DMSO-d_6 30

2.8 ^{1}H,^{1}H-ROESY NMR spectra of *R/S*-**5a**@*S,S*-**CT2** 31

2.9 Job Plot of Host and Guest 32

2.10 ESI-HRMS for Host-Guest Complexes 33

2.11 Determination of Association Constants of Hosts and Guests 34

2.12 X-Ray Single Crystallography 67

2.13 Variable Temperature ^{1}H NMR Experiments 74

2.14 Circular dichroism (CD), UV-Vis and Fluorescent Spectra of Hosts Interact with Guests (**5**) 76

2.15 Computational Data 82

2.16 Circular dichroism (CD), UV-Vis and Fluorescent Spectra of Hosts Interact with Guests (**4**) 89

3 Supplementary References 91

1. Supplementary Methods

1.1 General Method

All the reagents involved in this research were commercially available and used without further purification unless otherwise noted (2,6-Dihydroxynaphthalene, J&K Scientific, 95%; Methyl magnesium bromide (1.0 M THF solution), J&K Scientific; Trifluoroacetic acid, Adamas, 99%; K_2CO_3, bromobutane, 1,1,3,3-tetramethoxypropane, dichloromethyl methyl ether, *t*-Butyl carbamate, triethyl silane, N,N-Diisopropylethylamine, and titanium ethoxide, Energy-chemical, >95%; 1,1'-Thiocarbonyldiimidazole, Bidepharm, 85%; *tert*-butanesulfinamide, Bidepharm, 95%). Solvents (Energy-chemical, 99%) were either employed as purchased or dried before use by standard laboratory procedures. Thin-layer chromatography (TLC) was carried out on 0.25 mm Leyan silica gel plates (60F-254). Column chromatography was performed on silica gel (200-300 mesh) as the stationary phase. ^{1}H, ^{13}C NMR, 2D NMR spectra were performed on Bruker Avance-500 NMR spectrometers. Chemical shifts are reported in ppm with residual solvents or TMS (tetramethylsilane) as the internal standards. The following abbreviations were used for signal multiplicities: s, singlet; d, doublet; dd, doublet of doublet; m, multiplet. Host-guest complexes were prepared by simply mixing the guests and hosts in 1: 1 stoichiometry in the corresponding solvent. Electrospray-ionization high-resolution mass spectrometry (ESI-HRMS) experiments were conducted on an applied Q-EXACTIVE mass spectrometry system. Circular Dichroism (CD) and UV-Vis spectra were recorded on an Applied PhotoPhysics Chirascan CD spectropolarimeter, using a 1 cm quartz cuvette. Fluorescent spectra were recorded on a spectrofluorometer (Edinburgh FS5), using a 1 cm quartz cuvette. Specific rotations were measured on Rudolph Research Analytical Autopol I Polarimeter (589 nm) in a 1 dm length cell under 25 °C.

All ^{1}H NMR titration experiments were repeated 3 times, and the averaged values and standard deviations are given.

2. Supplementary Discussion

2.1 Synthetic Procedures of Chiral Naphthotubes

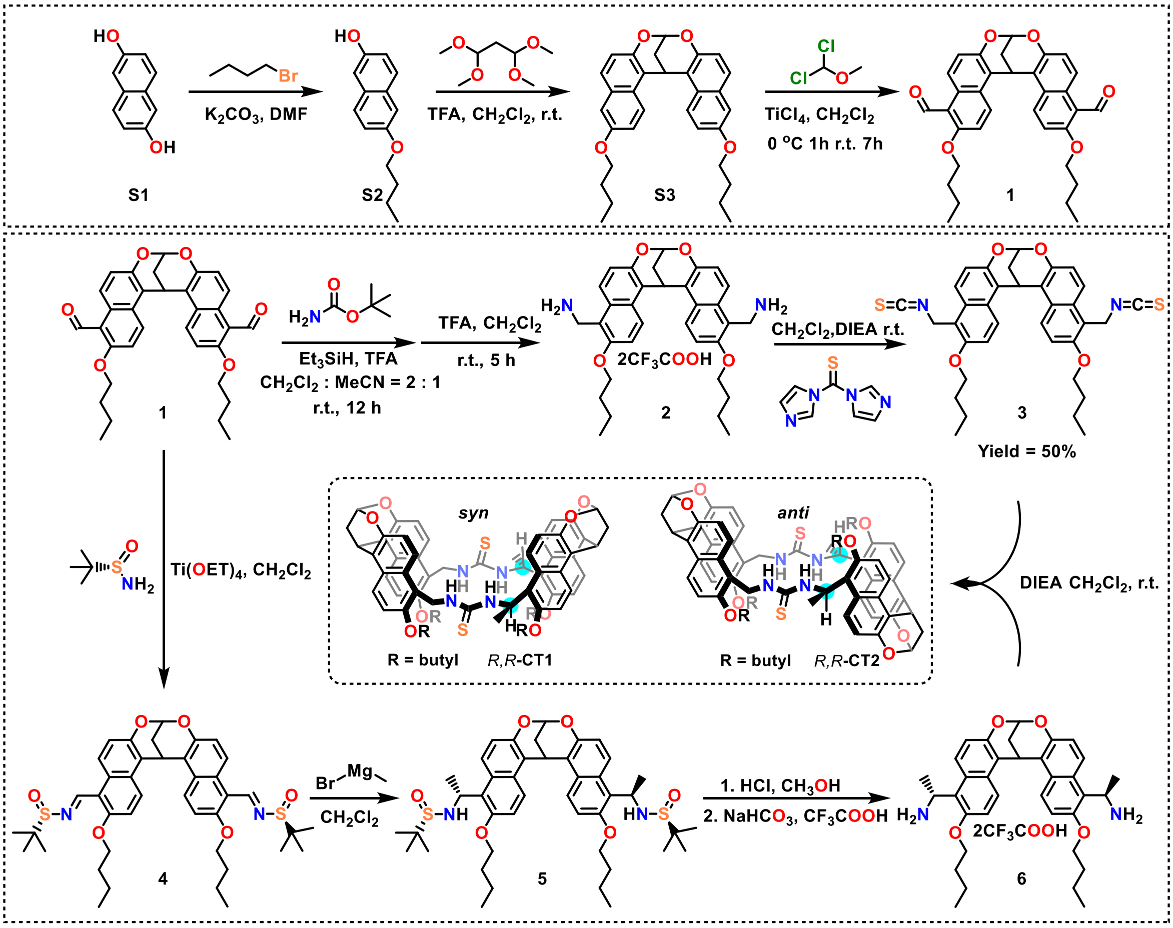

Supplementary Figure 1. **Synthetic procedures of naphthotubes**. The other pair of enantiomeric chiral macrocyclic compounds *S,S*-**CT1**, and *S,S*-**CT2** can be synthesized by similar methods.

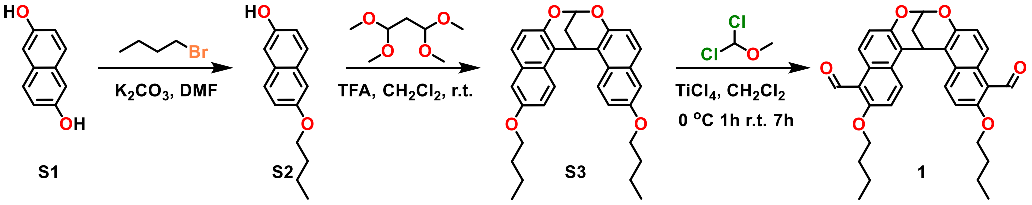

The synthesis of dialdehyde-substituted bis-naphthalene cleft **1** has been reported previously. [1] **S2**: 2,6-Dihydroxynaphthalene (**S1**, 1 mmol), K_2CO_3 (5 mmol), bromobutane (1 mmol), DMF, yield 30%; **S3**: 1,1,3,3-tetramethoxypropane (1 mmol), **S2** (2 mmol) TFA/CH_2Cl_2 (v/v = 1/2), yield 75%; **1**: dichloromethyl methyl ether (3 mmol), $TiCl_4$ (4 mmol), CH_2Cl_2, yield 80%.

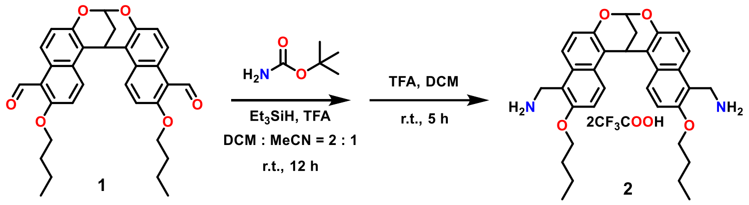

1 (5.0 g, 10 mmol, 1.0 equiv.) and *t*-Butyl carbamate (7.03 g, 60 mmol, 7.0 equiv.) were dissolved in the mixture of acetonitrile (60 mL) and dichloromethane (120 mL) in a 1 L two-neck flask under Ar atmosphere. To this solution, triethyl silane (9 mL) and trifluoroacetic acid (7.6 mL) were added through syringes. The resulting mixture was stirred at room temperature for another 12 h. The solvents were removed under reduced pressure. The crude product was dissolved in the mixture of dichloromethane (30 mL) and trifluoroacetic acid (15 mL). The solution was stirred at room temperature for 6 h. The solvents were removed under reduced pressure, and the residue was washed by ether to afford pure **2** as a white powder (6 g, yield 80%).

2: ^{1}H NMR (500 MHz, DMSO-d_6, 298K) δ [ppm] = 8.88 (d, J = 9.6 Hz, 2H), 8.17 (s, 6H), 7.88 (d, J = 9.3 Hz, 2H), 7.54 (d, J = 9.5 Hz, 2H), 7.22 (d, J = 9.2 Hz, 2H), 6.42 (q, J = 1.9 Hz, 1H), 5.70 (d, J = 3.6 Hz, 1H), 4.40 (s, 4H), 4.18 (ddt, J = 27.0, 9.3, 6.5 Hz, 4H), 2.47 (t, J = 2.5 Hz, 2H), 1.80 (dq, J = 9.0, 6.6 Hz, 4H), 1.54-1.47 (m, 4H), 0.98 (t, J = 7.4 Hz, 6H).

^{13}C NMR (126 MHz, DMSO-d_6, 298K) δ [ppm] = 159.03, 153.95, 148.77, 128.83, 126.98, 126.46, 123.46, 120.20, 119.71, 115.36, 114.46, 91.46, 68.97, 33.14, 31.43, 26.58, 22.15, 19.21, 14.28.

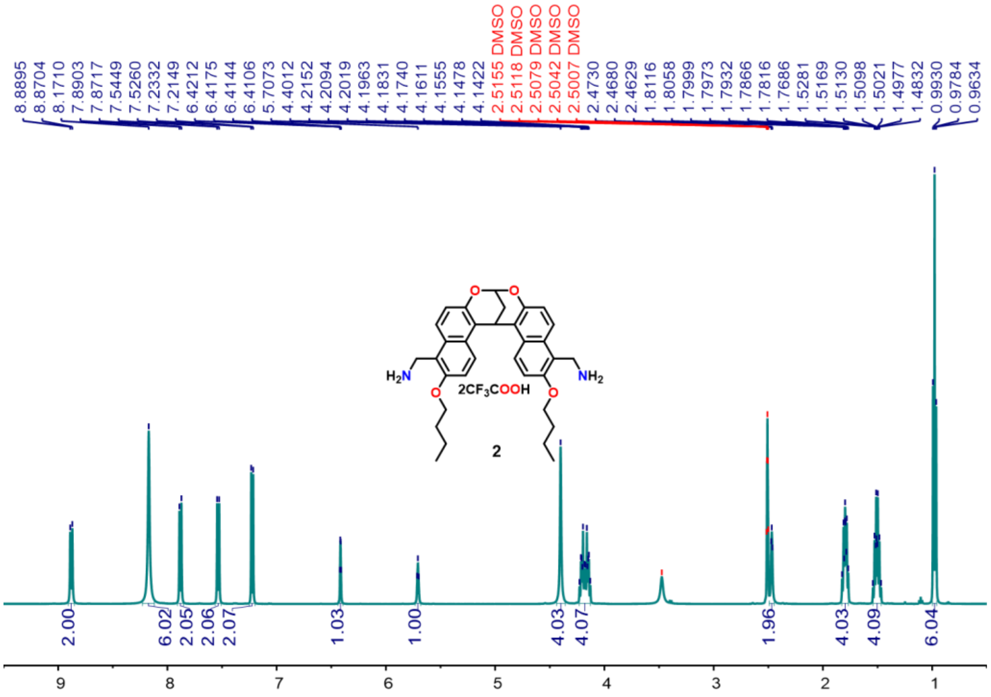

Supplementary Figure 2. ^{1}H NMR spectrum (500 MHz, DMSO-d_6, 298 K) of **2**.

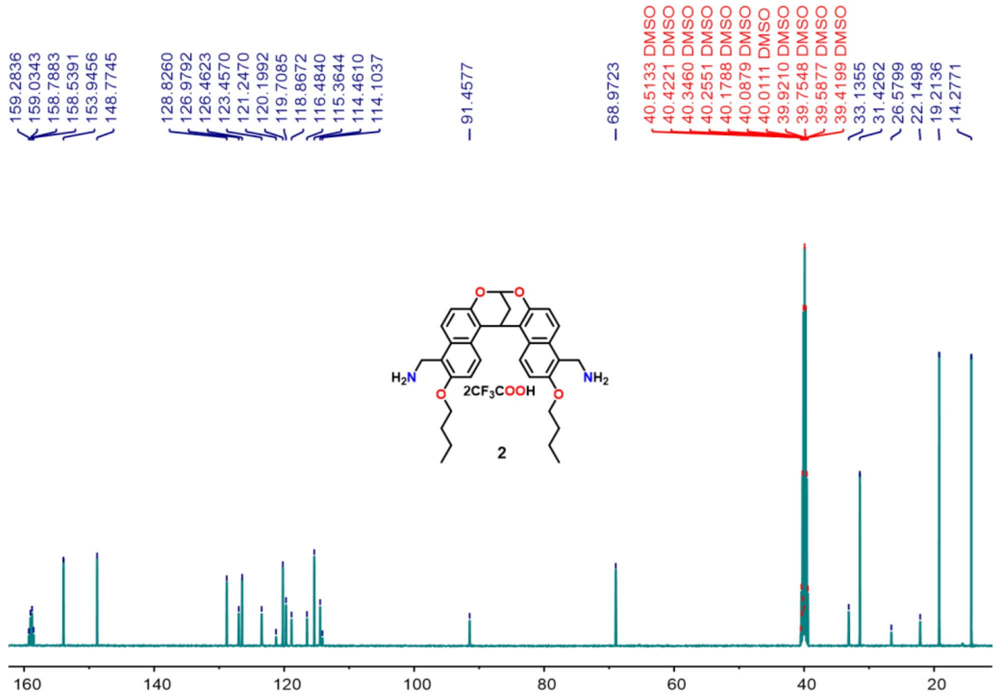

Supplementary Figure 3.^{13}C NMR spectrum (126 MHz, DMSO-d_6, 298 K) of **2**.

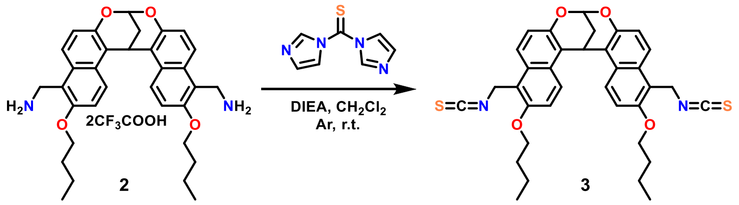

To the solution of 1, 1'-Thiocarbonyldiimidazole (5.2 g, 30 mmol) in CH_2Cl_2 (400 mL), added compound **2** (5.3 g, 10 mmol) and N,N-Diisopropylethylamine (DIEA, 2.6 g, 20 mmol) were added dropwise during 30 min. The resulting mixture was stirred at room temperature for 3 h. The solvent was removed under vacuum, and the residue was purified by column chromatography (SiO_2, Hexane / CH_2Cl_2 = 1 / 1) to give the compound **3** as a yellow solid (5.6 g, 92 %).

3: ^{1}H NMR (400 MHz, $CDCl_3$, 298K) δ [ppm] = 8.56 (d, J = 9.4 Hz, 2H), 7.69 (d, J = 9.2 Hz, 2H), 7.34 (d, J = 9.4 Hz, 2H), 7.28 (s, 1H), 7.26 (s, 1H), 5.35-5.27 (m, 1H), 5.11-4.92 (m, 4H), 4.14 (qt, J = 9.1, 6.5 Hz, 4H), 2.45 (dd, J = 3.1, 2.1 Hz, 2H), 1.90-1.78 (m, 4H), 1.54 (ddd, J = 9.5, 6.5, 5.1 Hz, 5H), 1.01 (t, J = 7.4 Hz, 6H).

^{13}C NMR (126 MHz, $CDCl_3$, 298K) δ 152.91, 148.85, 131.03, 128.72, 126.53, 125.25, 122.67, 120.32, 119.08, 116.03, 114.23, 91.37, 69.30, 39.26, 31.56, 26.87, 22.99, 19.36, 13.93.

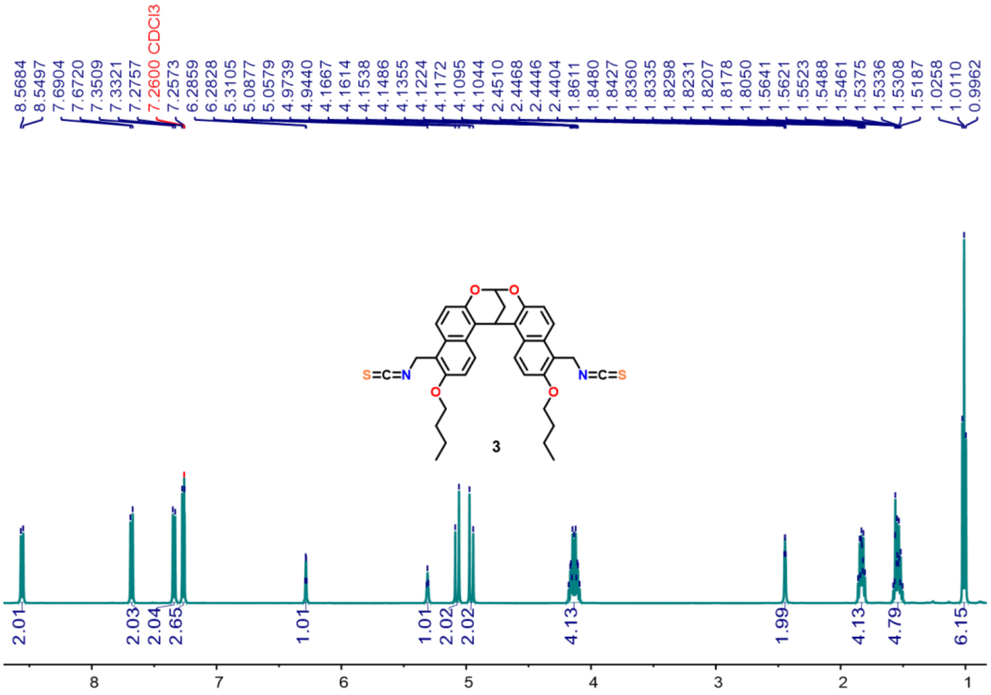

Supplementary Figure 4. ^{1}H NMR spectrum (500 MHz, $CDCl_3$, 298 K) of **3**.

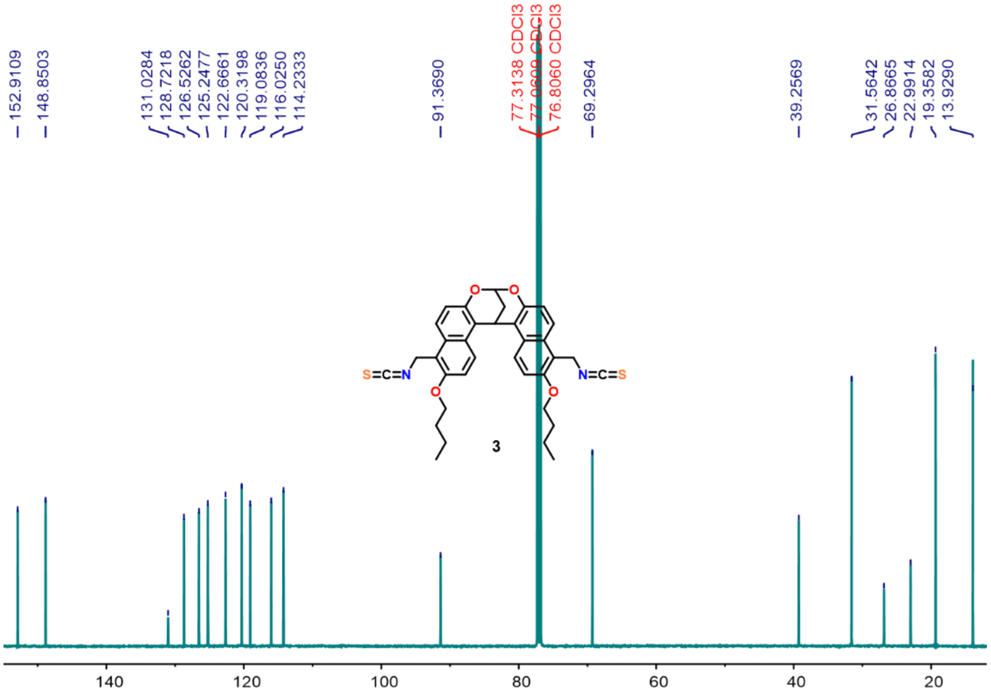

Supplementary Figure 5. ^{13}C NMR spectrum (126 MHz, $CDCl_3$, 298 K) of **3**.

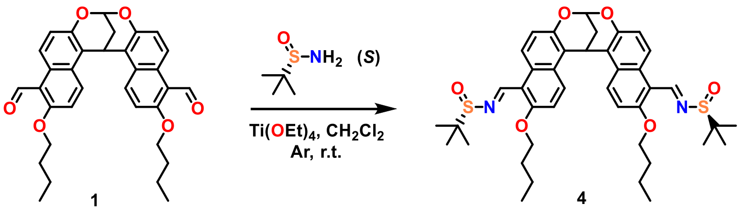

To a solution of **1** (1.05 g, 2 mmol) and *tert*-butanesulfinamide (1.45 g, 12 mmol) in CH_2Cl_2 (dry, 80 mL), titanium ethoxide (2.5 mL, 12 mmol) was added under Ar at 0 °C.

After stirring at 0 °C for 1 h, the resulting mixture was warmed to room temperature and stirred for another 12 h. The solution was then poured into ice water. The resulting suspension was extracted with CH_2Cl_2 (100 mL × 3). The combined organic phase was washed consecutively with H_2O (100 mL) and then dried over anhydrous Na_2SO_4. After removing the CH_2Cl_2, the solid was washed with n-hexane and the **4** (1.4 g, yield 97%, yellow solid) was obtained.

4: ^{1}H NMR (500 MHz, $CDCl_3$, 298K) δ[ppm] = 9.40 (d, *J* = 2.7 Hz, 2H), 9.17 (dd, *J* = 17.5, 9.4 Hz, 2H), 8.66 (d, *J* = 9.5 Hz, 2H), 7.36 (dd, *J* = 9.5, 2.8 Hz, 2H), 7.24 (dd, *J* = 9.4, 1.5 Hz, 2H), 6.27 (q, *J* = 1.8 Hz, 1H), 5.30 (d, *J* = 3.2 Hz, 1H), 4.17 (dtq, *J* = 12.7, 6.5, 2.9 Hz, 4H), 2.45 (t, *J* = 2.6 Hz, 2H), 1.87 – 1.81 (m, 4H), 1.55 – 1.49 (m, 4H), 1.28 (s, 6H), 1.21 (d, *J* = 1.8 Hz, 12H), 0.97 (td, *J* = 7.5, 2.4 Hz, 6H).

^{13}C NMR (126 MHz, $CDCl_3$, 298K) δ[ppm] = 160.23, 160.10, 158.27 (d, *J* = 6.3 Hz), 149.07, 128.90 (d, *J* = 6.0 Hz), 128.29, 126.50, 126.39, 125.90 (d, *J* = 12.5 Hz), 120.97 (d, *J* = 1.7 Hz), 118.82, 118.75, 116.18, 114.08 (d, *J* = 5.0 Hz), 91.23, 69.62 (d, *J* = 1.9 Hz), 57.19 (d, *J* = 5.0 Hz), 31.33, 26.96, 23.02, 22.60, 22.50, 22.13, 19.22, 13.86.

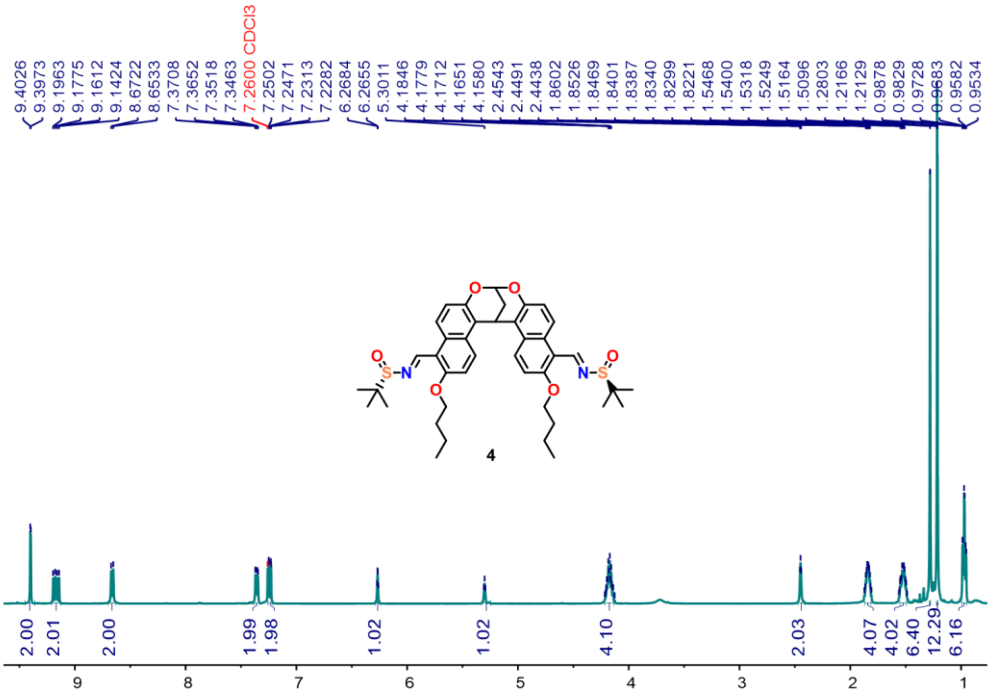

Supplementary Figure 6. ^{1}H NMR spectrum (500 MHz, $CDCl_3$, 298 K) of **4**.

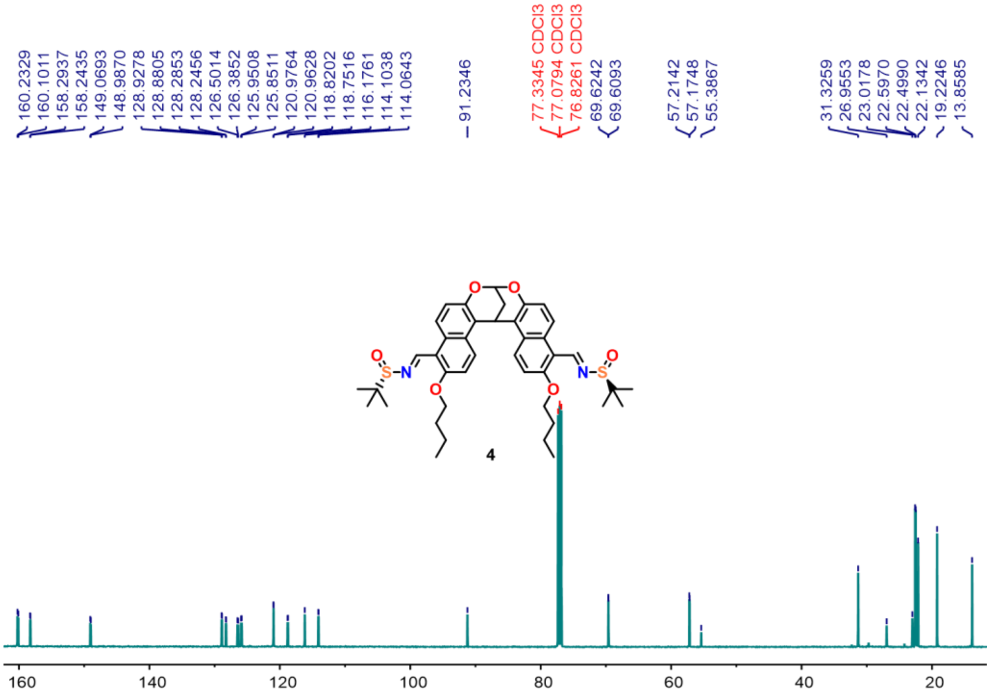

Supplementary Figure 7. ^{13}C NMR spectrum (126 MHz, $CDCl_3$, 298 K) of **4**.

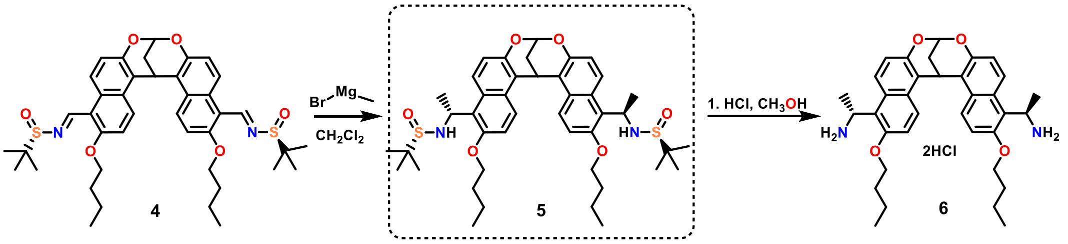

To a solution of **4** (2.92 g, 4 mmol) in dry CH_2Cl_2 (200 mL) at 0°C was added methyl magnesium bromide (40 mL of a 1.0 M THF solution) dropwise under Ar. The reaction mixture was stirred at 0°C for 2 h, the reaction was continued overnight at room temperature. A saturated ammonium chloride solution was added to the reaction mixture after cooling in an ice bath. The reaction mixture was stirred for 30 min, and then the resulting suspension was extracted with CH_2Cl_2 (100 mL × 3). The combined organic phase was washed consecutively with H_2O (100 mL×3) and then dried over anhydrous Na_2SO_4. After removing the CH_2Cl_2, the solid was washed with n-hexane to provide **5** (3.04 g, yield 99%, pale white solid), then **5** was used directly for the next step without further purification. To a solution of **5** (0.97 g, 1.3 mmol) in 20 mL of MeOH was added 0.5 mL (6 mmol) of 12 M HCl solution at room temperature, and the mixture was stirred for 30 min. After removing MeOH, the solid was washed with diethyl ether (20 mL×3), and the **6** (0.64 g, yield 91%, pale white solid) was obtained. Specific rotation (*R,R*-**6**: $[\alpha]_D^{25}$ = +0.9 (*c*, 0.01, MeOH), *S,S*-**6**: $[\alpha]_D^{25}$ = -0.7 (*c*, 0.01, MeOH)).

6: ^{1}H NMR (500 MHz, DMSO-d_6, 298K) δ [ppm] = ^{1}H NMR (500 MHz, DMSO-d_6) δ 8.90 – 8.79 (m, 2H), 8.44 (s, 2H), 8.35 (s, 1H), 7.92 (d, *J* = 9.4 Hz, 1H), 7.87 (d, *J* = 9.2 Hz, 1H), 7.53 (dd, *J* = 21.0, 9.5 Hz, 2H), 7.21 (dd, *J* = 9.3, 5.8 Hz, 2H), 6.41 (s, 1H),

5.72 (s, 1H), 5.15 (p, *J* = 6.1 Hz, 1H), 5.12 – 5.06 (m, 1H), 4.25 (t, *J* = 7.0 Hz, 3H), 4.21 – 4.14 (m, 1H), 2.45 (s, 2H), 1.87 – 1.78 (m, 5H), 1.62 (d, *J* = 6.8 Hz, 3H), 1.54 (d, *J* = 6.8 Hz, 3H), 1.46 (tq, *J* = 15.8, 7.9, 7.3 Hz, 6H), 0.97 (q, *J* = 7.4 Hz, 8H).

^{13}C NMR (126 MHz, DMSO-d_6) δ 153.12, 148.73, 148.53, 127.72, 127.63, 126.79, 126.60, 126.36, 122.72 (d, *J* = 30.2 Hz), 120.35, 120.23, 119.59 (dd, *J* = 51.9, 17.6 Hz), 114.75, 91.37, 68.84 (d, *J* = 15.2 Hz), 31.09 (d, *J* = 5.0 Hz), 26.58, 23.28, 22.20, 19.28, 19.12, 18.96, 14.31 (d, *J* = 2.3 Hz).

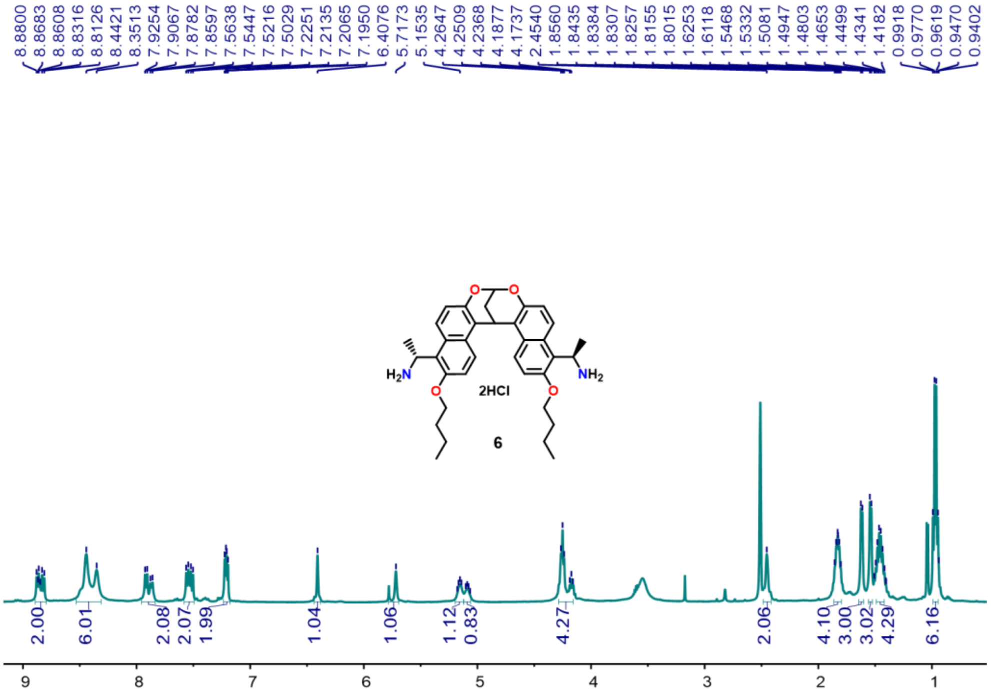

Supplementary Figure 8. ^{1}H NMR spectrum (500 MHz, DMSO-d_6, 298 K) of **6**.

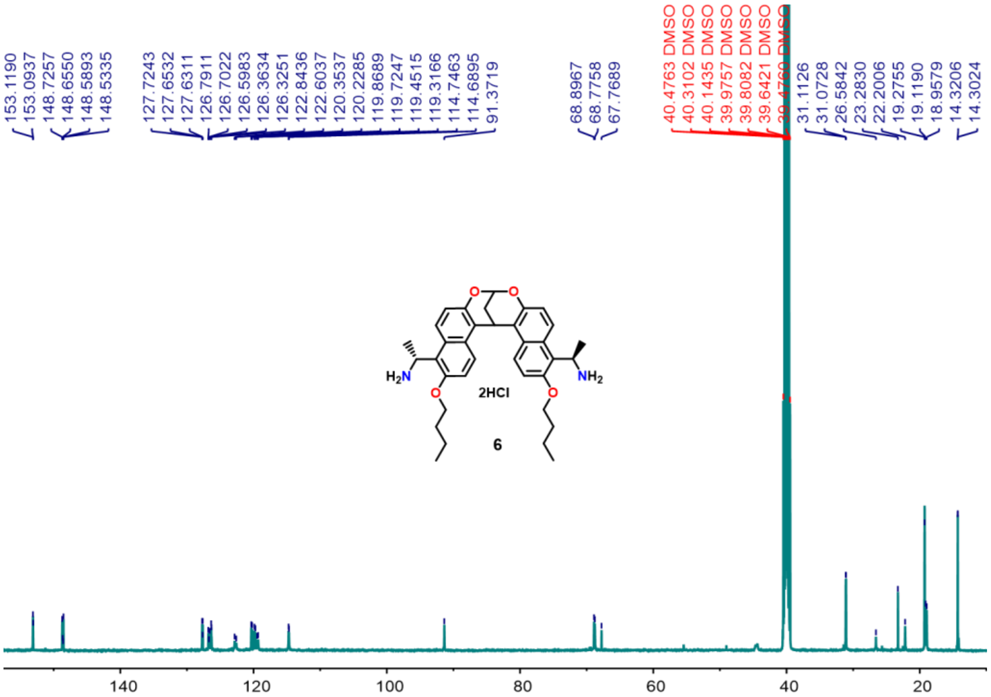

Supplementary Figure 9. ^{13}C NMR spectrum (126 MHz, $CDCl_3$, 298 K) of **6**.

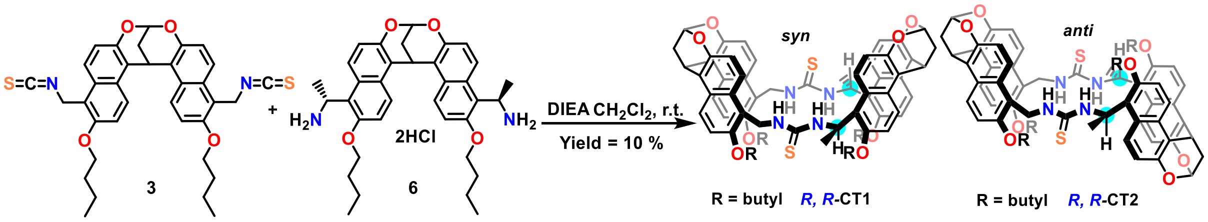

The solutions of compounds **3** (561 mg, 0.92 mmol; in 60 mL CH_2Cl_2) and **6** (577 mg, 0.92 mmol; in 60 mL CH_2Cl_2) in two separate syringes were added dropwise via a double-channel syringe pump to the solution of Hünig's base (545 mg, 5.0 mmol) in CH_2Cl_2 (400 mL) during the course of 10 h. The resulting mixture was stirred at room temperature for 24 h. The solvent was removed in vacuum, and the residue was purified by column chromatography (SiO_2, CH_2Cl_2: MeOH = 1000 / 5) to give the compound *R*,*R*-**CT1** and *R*,*R*-**CT2** as a white solid (54mg, 5.0 %, 55mg, 5.1 %). Specific rotation (*R,R*-**CT1**: $[\alpha]_D^{25}$ = +135.0 (*c*, 0.002, DCE), *R,R*-**CT2**: $[\alpha]_D^{25}$ = +10.0 (*c*, 0.002, DCE)).

R,*R*-**CT1**: ^{1}H NMR (500 MHz, Toluene-d_8, 298 K) δ [ppm] = 8.65 (d, *J* = 9.2 Hz, 1H), 8.39 (d, *J* = 9.3 Hz, 2H), 8.34 (d, *J* = 9.4 Hz, 1H), 8.27 (dd, *J* = 12.3, 9.4 Hz, 2H), 7.74 (d, *J* = 9.3 Hz, 1H), 7.29 (dd, *J* = 9.2, 6.0 Hz, 2H), 7.16 (d, *J* = 9.1 Hz, 2H), 7.13 – 7.10 (m, 2H), 6.98 (d, *J* = 9.1 Hz, 1H), 6.93 (dd, *J* = 9.3, 5.8 Hz, 3H), 6.49 (d, *J* = 8.9 Hz, 2H), 6.20 (dd, *J* = 13.7, 5.8 Hz, 1H), 5.92 – 5.85 (m, 2H), 5.57 (s, 1H), 5.16 (d, *J* = 8.9 Hz, 1H), 4.98 – 4.94 (m, 2H), 4.89 (s, 1H), 4.68 (d, *J* = 14.0 Hz, 1H), 4.49 (d, *J* = 13.6 Hz, 1H), 3.92 – 3.83 (m, 2H), 3.76 – 3.61 (m, 4H), 3.52 (dt, *J* = 9.1, 6.6 Hz, 1H), 3.37 (dt, *J* = 9.0, 6.7 Hz, 1H), 1.99 (ddd, *J* = 12.5, 3.8, 1.8 Hz, 1H), 1.89 – 1.17 (m, 24H), 0.98 (t, *J* = 7.4 Hz, 3H), 0.92 (d, *J* = 7.4 Hz, 3H), 0.85 (t, *J* = 7.4 Hz, 3H), 0.73 (t, *J* = 7.4 Hz, 3H).

^{13}C NMR (126 MHz, Toluene-d_8, 298 K) δ [ppm] = 183.56, 181.47, 153.05, 152.97, 152.59, 151.61, 150.17, 149.95, 148.30, 147.85, 137.12, 129.76, 129.42, 128.70, 128.51, 128.32, 127.89, 127.79, 127.60, 127.41, 126.76, 126.40, 124.96, 124.76, 124.57, 123.96, 123.63, 123.50, 123.42, 121.69, 120.75, 120.49, 120.46, 120.07, 119.78, 118.91, 118.81, 118.24, 117.99, 115.39, 114.53, 113.42, 113.16, 91.33, 91.08, 70.07, 69.30, 68.67, 67.78, 65.55, 48.20, 46.67, 39.12, 39.09, 31.71, 31.59, 31.23, 26.71, 26.24, 23.19, 22.82, 21.03, 20.52, 20.37, 20.21, 20.06, 19.91, 19.76, 19.60, 19.34, 19.26, 19.25, 19.22, 15.19, 13.72, 13.69, 13.61, 13.35.

R,*R*-**CT1**: ^{1}H NMR (500 MHz, DMSO-d_6, 298 K) δ [ppm] = 8.83 (d, *J* = 9.4 Hz, 1H), 8.75 (d, *J* = 9.4 Hz, 1H), 8.42 (d, *J* = 9.5 Hz, 1H), 8.24 (d, *J* = 9.4 Hz, 1H), 7.88 (d, *J* = 9.6 Hz, 1H), 7.71 (d, *J* = 9.3 Hz, 2H), 7.54 (dd, *J* = 9.1, 4.3 Hz, 2H), 7.50 (d, *J* = 9.3 Hz, 2H), 7.20 (d, *J* = 9.2 Hz, 2H), 7.12 (dd, *J* = 8.9, 5.1 Hz, 2H), 7.06 (d, *J* = 9.3 Hz, 2H), 6.96 (d, *J* = 9.2 Hz, 1H), 6.92 (d, *J* = 4.9 Hz, 1H), 6.88 – 6.85 (m, 1H), 6.38 (p, *J* = 6.9 Hz, 1H), 6.34 – 6.27 (m, 2H), 6.21 – 6.12 (m, 1H), 5.61 – 5.55 (m, 1H), 5.51 (d, *J* = 24.7 Hz, 2H), 5.25 (dd, *J* = 13.3, 4.1 Hz, 1H), 4.52 (d, *J* = 13.3 Hz, 1H), 4.16 (t, *J* = 6.3 Hz, 2H), 4.11 (t, *J* = 6.4 Hz, 2H), 4.01 – 3.91 (m, 2H), 3.84 (ddt, *J* = 16.0, 9.2, 6.2 Hz,

2H), 2.57 – 2.52 (m, 2H), 2.32 – 2.19 (m, 2H), 1.76 – 1.40 (m, 22H), 0.98 – 0.85 (m, 12H).

^{13}C NMR (126 MHz, DMSO-d_6, 298 K) δ [ppm] = 153.48, 153.21, 152.50, 150.95, 149.97, 149.90, 147.42, 146.91, 127.83, 127.41, 127.38, 127.27, 126.54, 125.60, 125.43, 125.13, 124.71, 124.59, 124.44, 124.05, 123.14, 121.22, 120.82, 120.38, 120.18, 119.30, 119.17, 118.81, 117.64, 115.82, 115.13, 115.03, 113.99, 91.66, 91.51, 69.39, 69.15, 69.08, 68.16, 65.41, 47.08, 45.77, 38.22, 31.63, 31.61, 31.51, 31.42, 26.97, 26.72, 22.54, 22.17, 21.30, 19.93, 19.37, 19.26, 19.21, 19.13, 15.66, 14.27, 14.23, 14.14.

ESI-HRMS: m/z calculated for *R*,*R*-**CT1** [M + H]$^+$ $C_{70}H_{77}N_4O_8S_2$: 1165.5178, found 1165.5173 (error = -0.4 ppm).

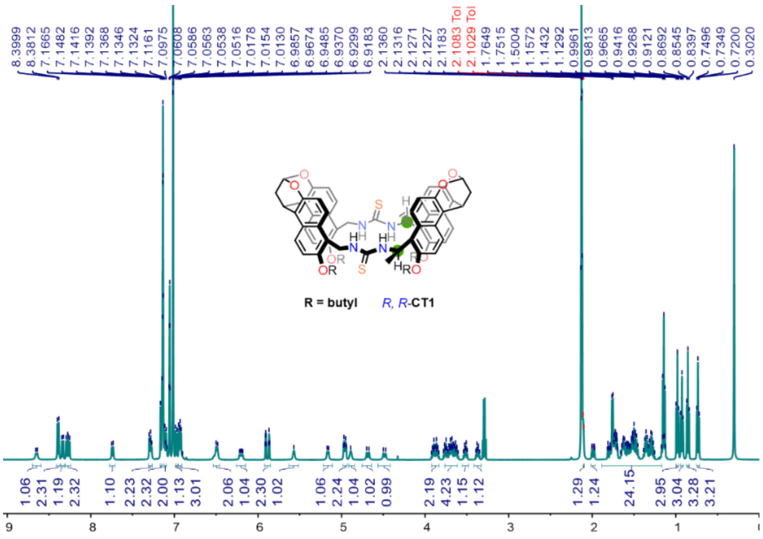

Supplementary Figure 10. ^{1}H NMR spectrum (500 MHz, Toluene-d_8, 298 K) of *R*,*R*-**CT1**.

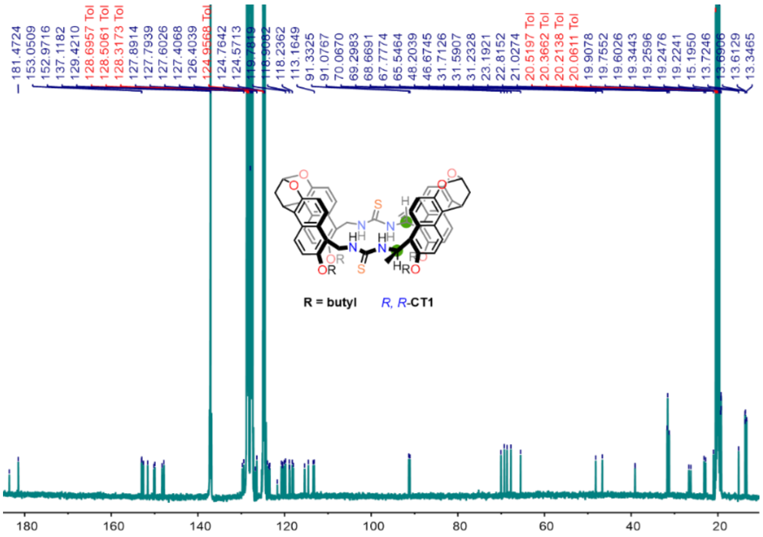

Supplementary Figure 11. ^{13}C NMR spectrum (126 MHz, Toluene-d_8, 298 K) of *R*,*R*-**CT1**.

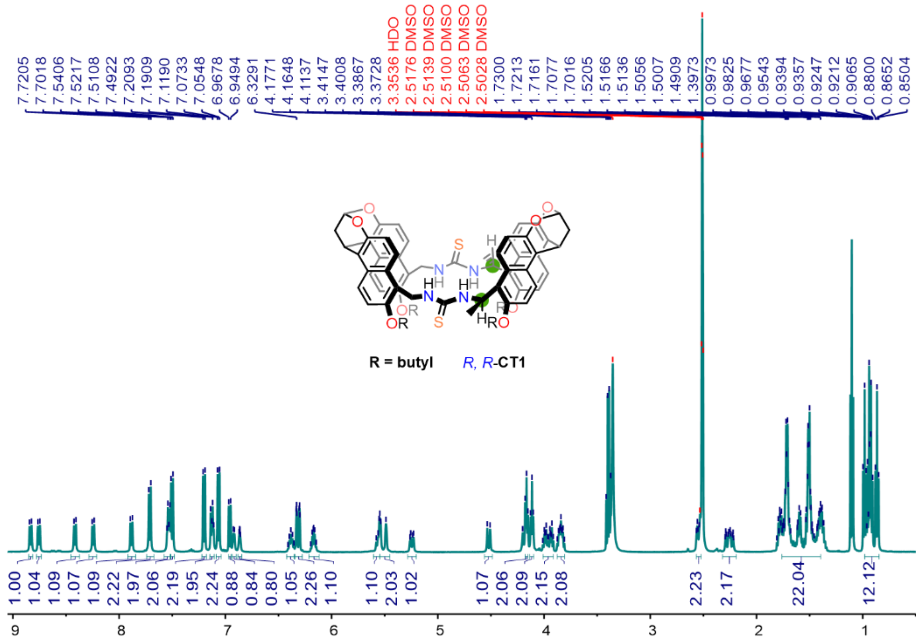

Supplementary Figure 12. ^{1}H NMR spectrum (500 MHz, DMSO-d_6, 298 K) of *R*,*R*-**CT1**.

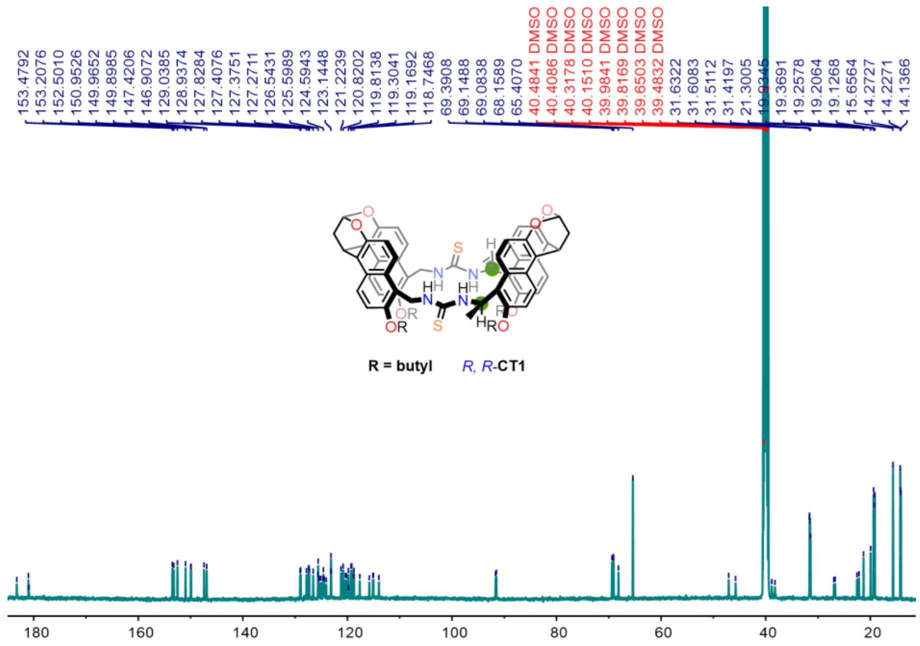

Supplementary Figure 13. ^{13}C NMR spectrum (126 MHz, DMSO-d_6, 298 K) of *R*,*R*-**CT1**.

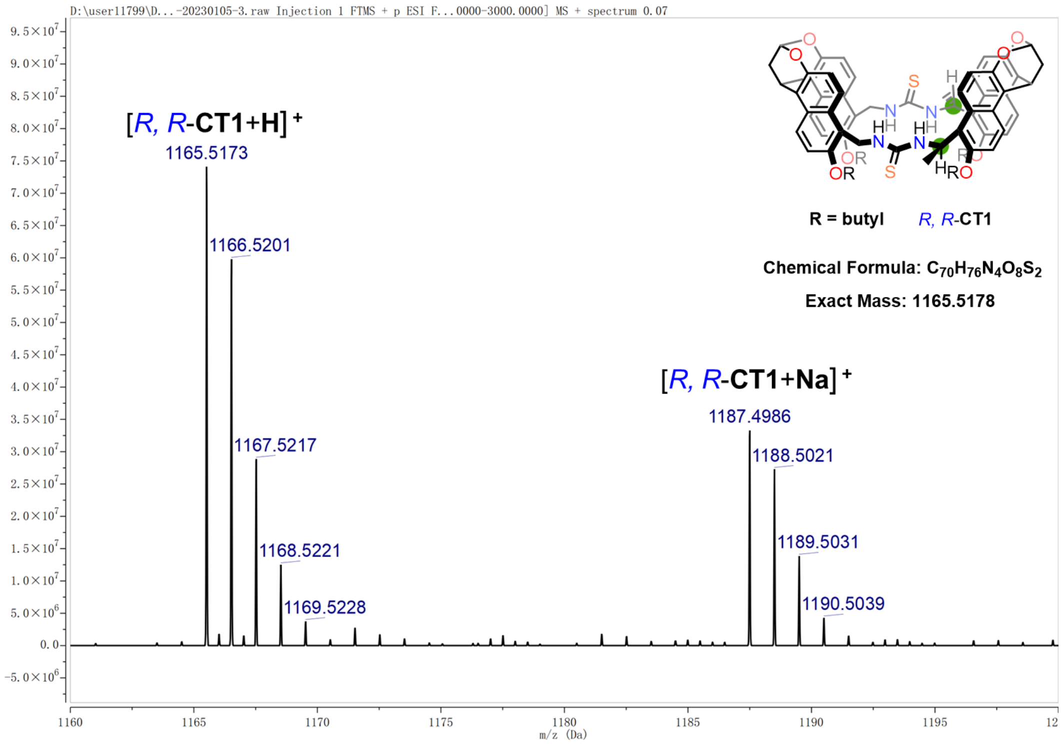

Supplementary Figure 14. ESI mass spectrum of *R*,*R*-**CT1**.

R,*R*-**CT2**: ^{1}H NMR (500 MHz, CD_2Cl_2, 298K) δ [ppm] = 8.30 (t, *J* = 10.1 Hz, 3H), 8.13 (d, *J* = 9.4 Hz, 1H), 7.86 – 7.65 (m, 4H), 7.26 – 7.08 (m, 3H), 7.06 – 7.00 (m, 2H), 6.98 (s, 1H), 6.97 – 6.92 (m, 2H), 6.41 (s, 1H), 6.13 – 6.07 (m, 2H), 5.72 – 5.64 (m, 1H), 5.51 (s, 1H), 5.18 (d, *J* = 7.9 Hz, 1H), 5.12 (dd, *J* = 5.7, 3.1 Hz, 2H), 4.49 (d, *J* = 13.8 Hz, 1H), 3.93 (hept, *J* = 16.8, 15.9 Hz, 9H), 2.40 – 2.18 (m, 4H), 1.73 (dq, *J* = 19.6, 6.3 Hz, 9H), 1.61 (t, *J* = 7.5 Hz, 2H), 1.47 (d, *J* = 2.6 Hz, 9H), 1.34 (q, *J* = 7.5 Hz, 2H), 0.92 (ddd, *J* = 9.5, 7.3, 2.0 Hz, 9H), 0.85 (t, *J* = 7.4 Hz, 3H).

^{13}C NMR (126 MHz, CD_2Cl_2, 298K) δ [ppm] = 124.22, 124.05, 123.98, 123.65, 123.46, 119.39, 119.23, 119.17, 118.35, 115.88, 114.03, 113.89, 91.71, 91.60, 70.19, 69.42, 68.88, 68.68, 53.89, 53.68, 53.46, 53.25, 53.03, 48.35, 46.67, 39.15, 31.73, 31.67, 31.51, 27.05, 26.79, 23.21, 22.95, 20.66, 20.06, 19.60, 19.39, 19.34, 19.29, 13.74, 13.65.

ESI-HRMS: m/z calculated for *R*,*R*-**CT2** $[M + H]^+$ $C_{70}H_{77}N_4O_8S_2$: 1165.5178, found 1165.5178 (no error).

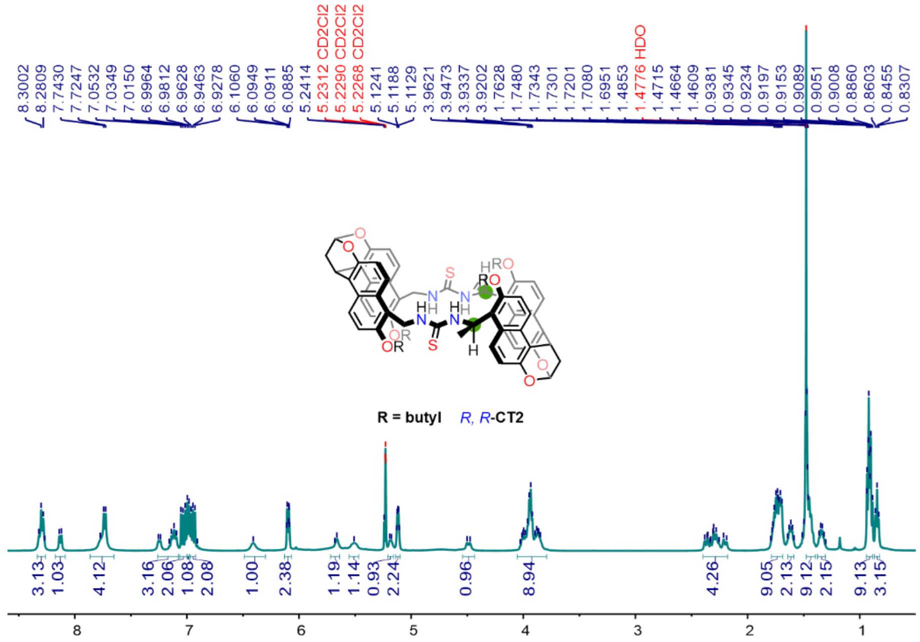

Supplementary Figure 15. ^{1}H NMR spectrum (500 MHz, CD_2Cl_2, 298 K) of *R*,*R*-**CT2**.

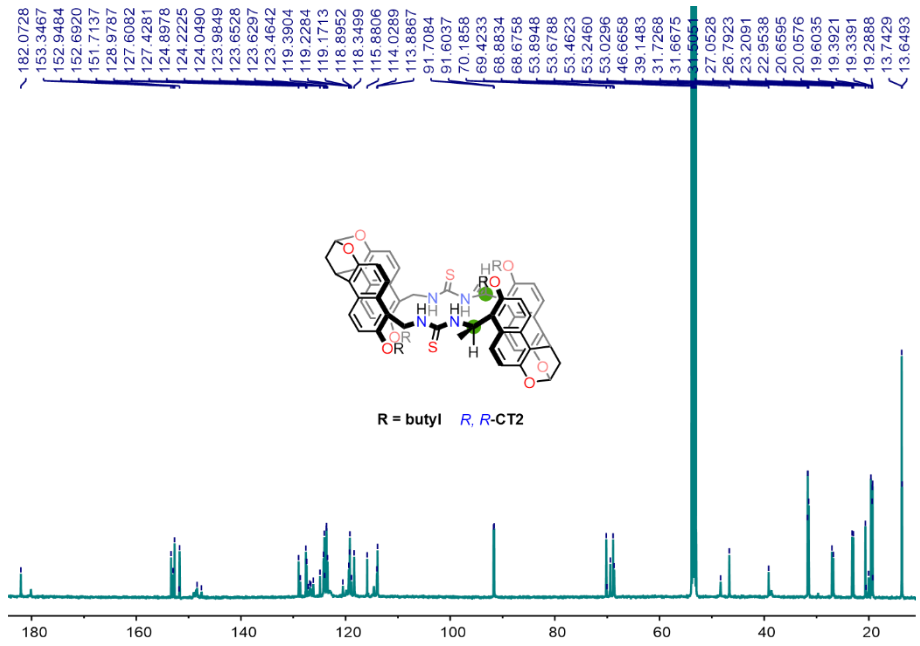

Supplementary Figure 16. ^{13}C NMR spectrum (126 MHz, CD_2Cl_2, 298 K) of *R*,*R*-**CT2**.

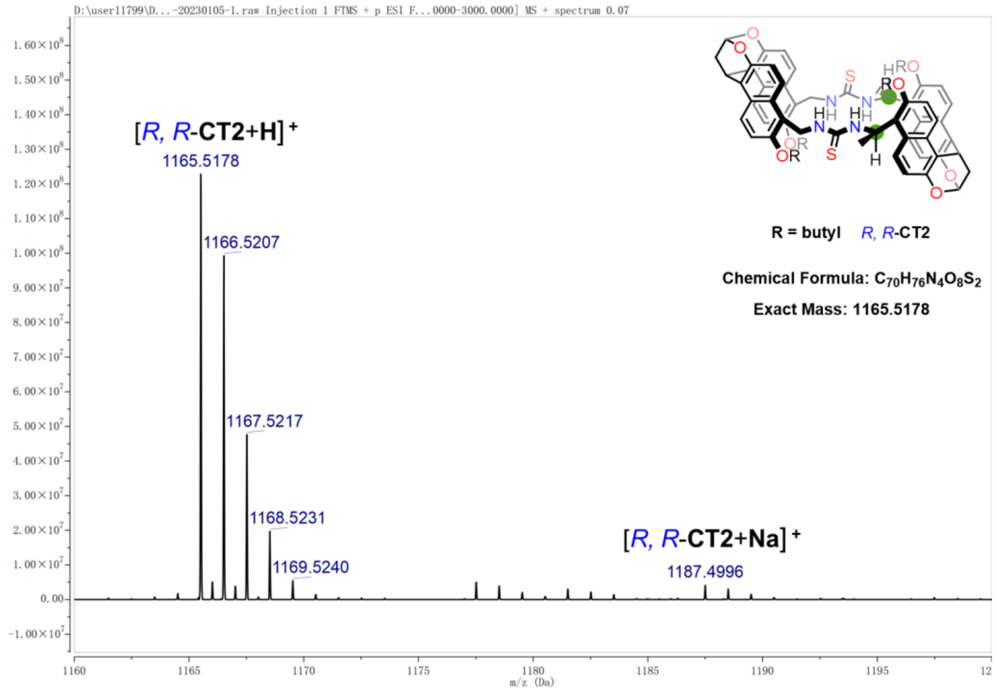

Supplementary Figure 17. ESI mass spectrum of *R*,*R*-**CT2**.

S,S-**CT1** and *S,S*-**CT2:** The NMR spectra of enantiomers including *S,S*-**CT1** and *S,S*-**CT2** are consistent with *R,R*-**CT1** and *R,R*-**CT2** respectively. **ESI-HRMS:** m/z calculated for *S,S*-**CT1** [M + H]$^+$ $C_{70}H_{77}N_4O_8S_2$: 1165.5178, found 1165.5175 (error = -0.3 ppm). m/z calcd for *S,S*-**CT2** [M + H]$^+$ $C_{70}H_{77}N_4O_8S_2$: 1165.5178, found 1165.5173 (error = -0.4 ppm). Specific rotation (*S,S*-**CT1**: $[\alpha]_D^{25}$ = -136.7 (*c*, 0.002, DCE), *S,S*-**CT2**: $[\alpha]_D^{25}$ = -10.0 (*c*, 0.002, DCE)).

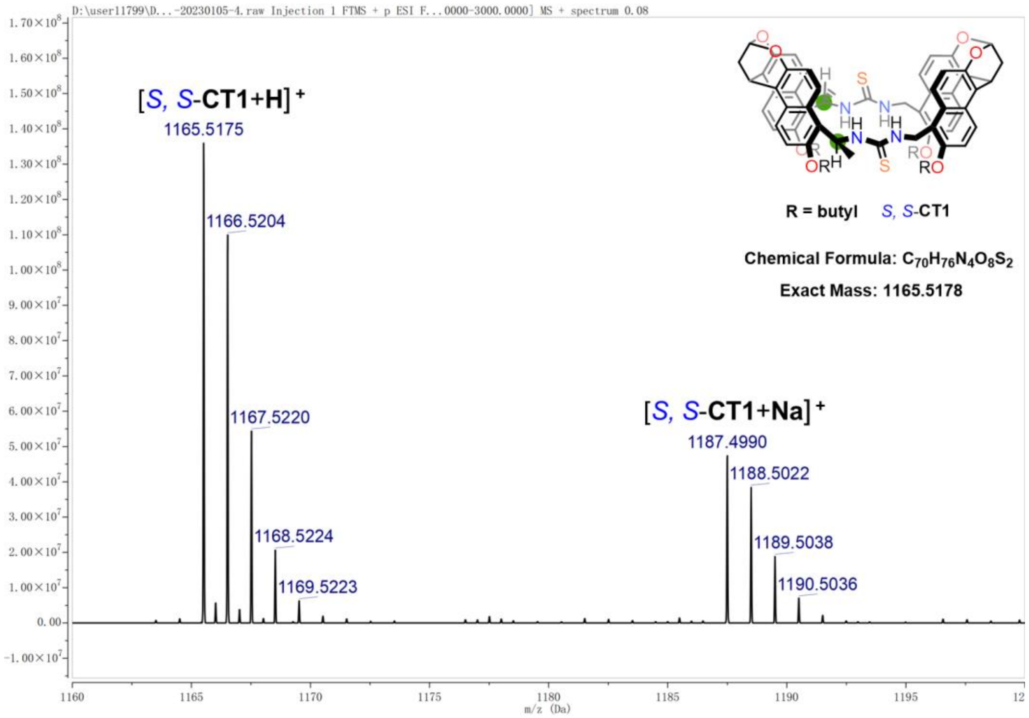

Supplementary Figure 18. ESI mass spectrum of *S,S*-**CT1**.

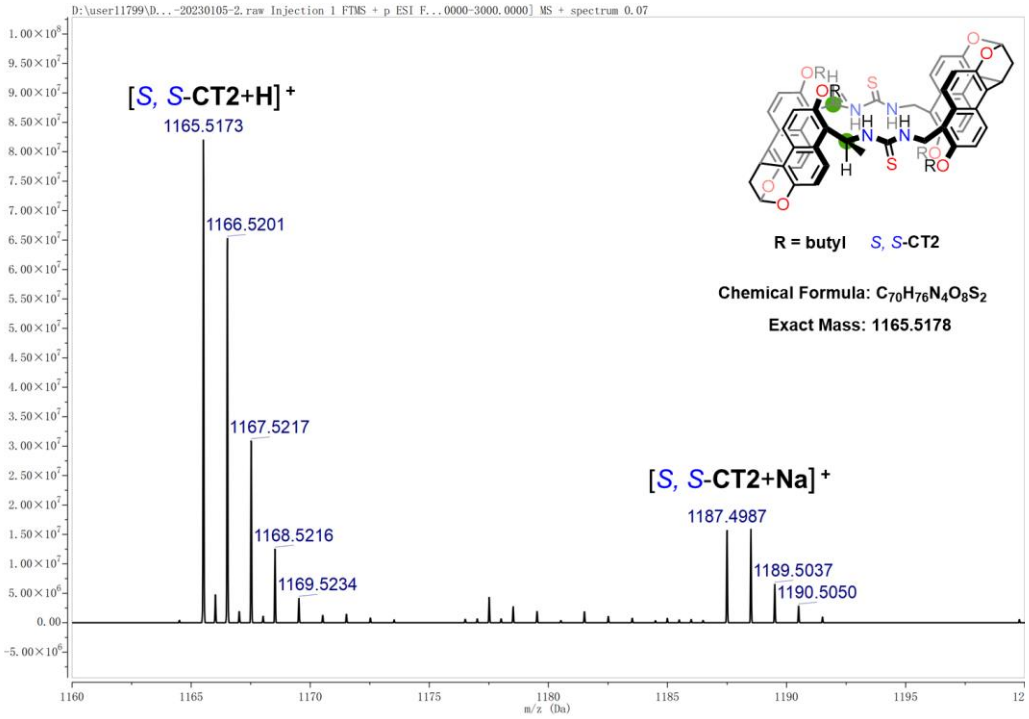

Supplementary Figure 19. ESI mass spectrum of *S,S*-**CT2**.

2.2 2D NMR spectra of Chiral Naphthotubes

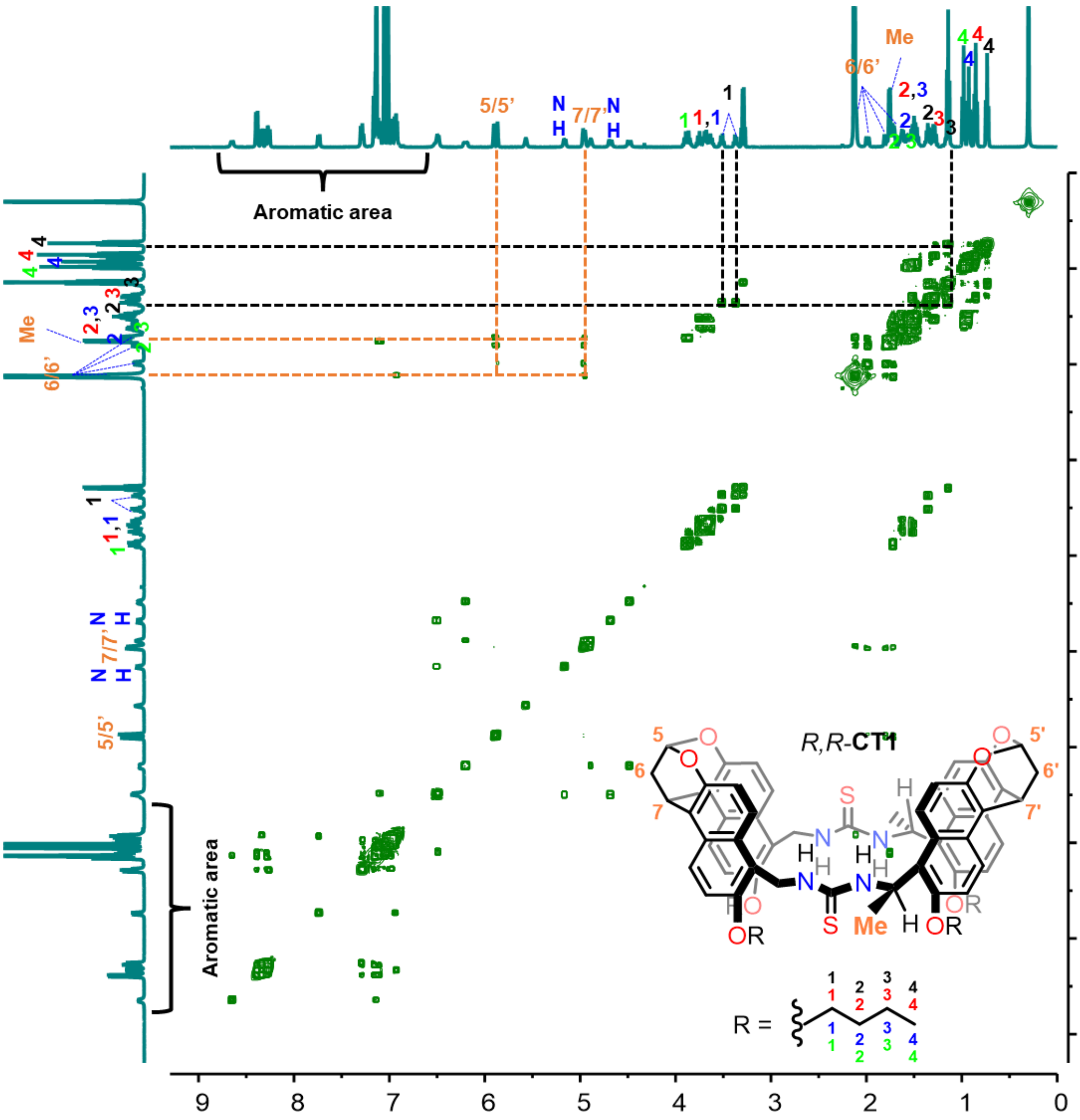

Supplementary Figure 20. 2D NMR spectra of naphthotubes. $^{1}H,^{1}H$-COSY NMR spectrum of *R,R*-**CT1** (500 MHz, Toluene-d$_8$, 298 K), the introduction of a chiral center reduces the symmetry of the naphthotube, and the protons on the four side chains are clearly distinguished. The 2D NMR spectra of *S,S*-**CT1** are consistent with *R,R*-**CT1**.

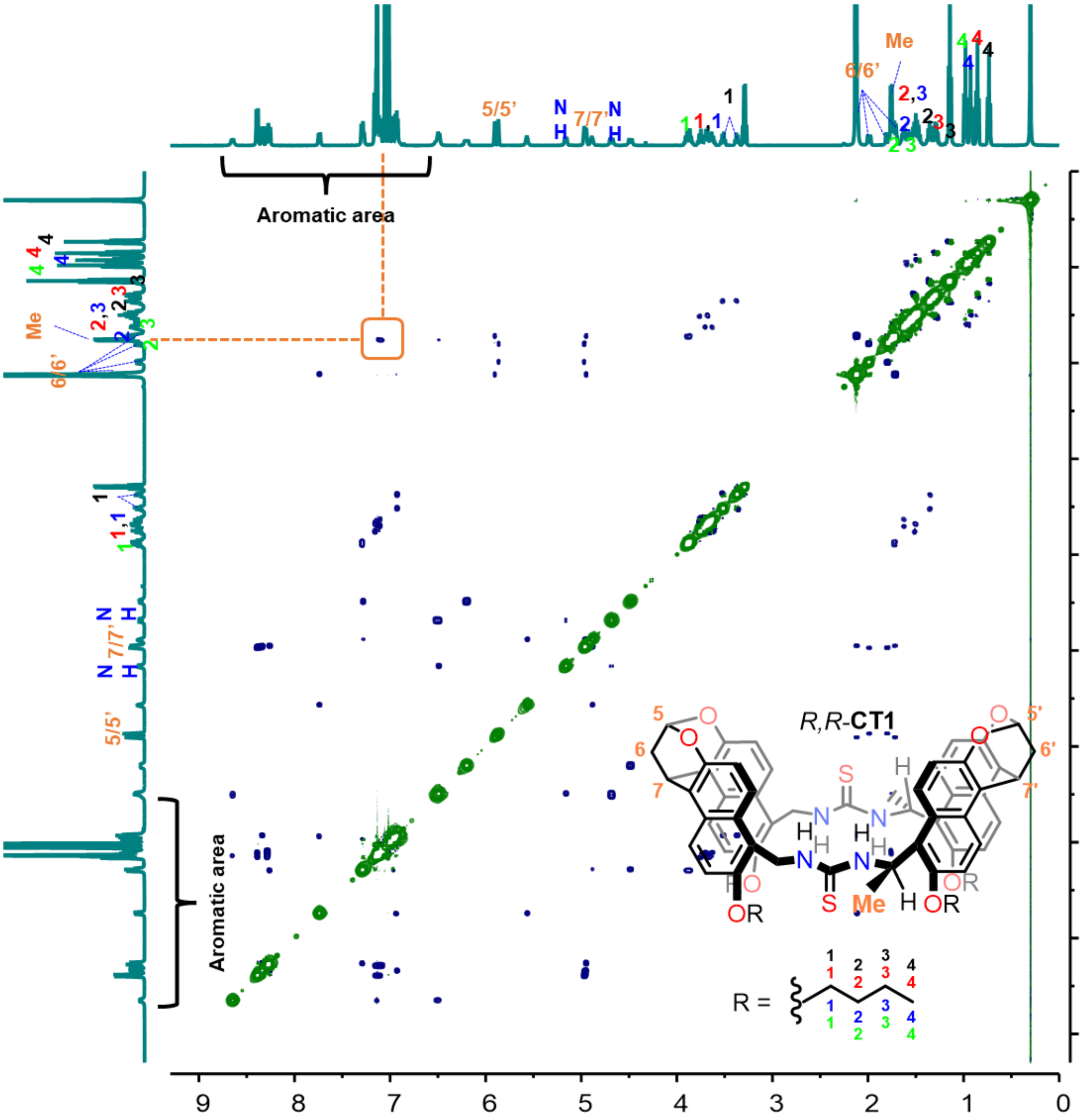

Supplementary Figure 21. 2D NMR spectra of naphthotubes. $^{1}H,^{1}H$-ROESY NMR spectrum of *R,R*-**CT1** (500 MHz, Toluene-d_8, 298 K). The NOE signal between one of two methyl groups in chiral centers and aromatic protons, as shown by the orange box, indicating that the methyl group is close to the bis-naphthalene cleft. The 2D NMR spectra of *S,S*-**CT1** are consistent with *R,R*-**CT1**.

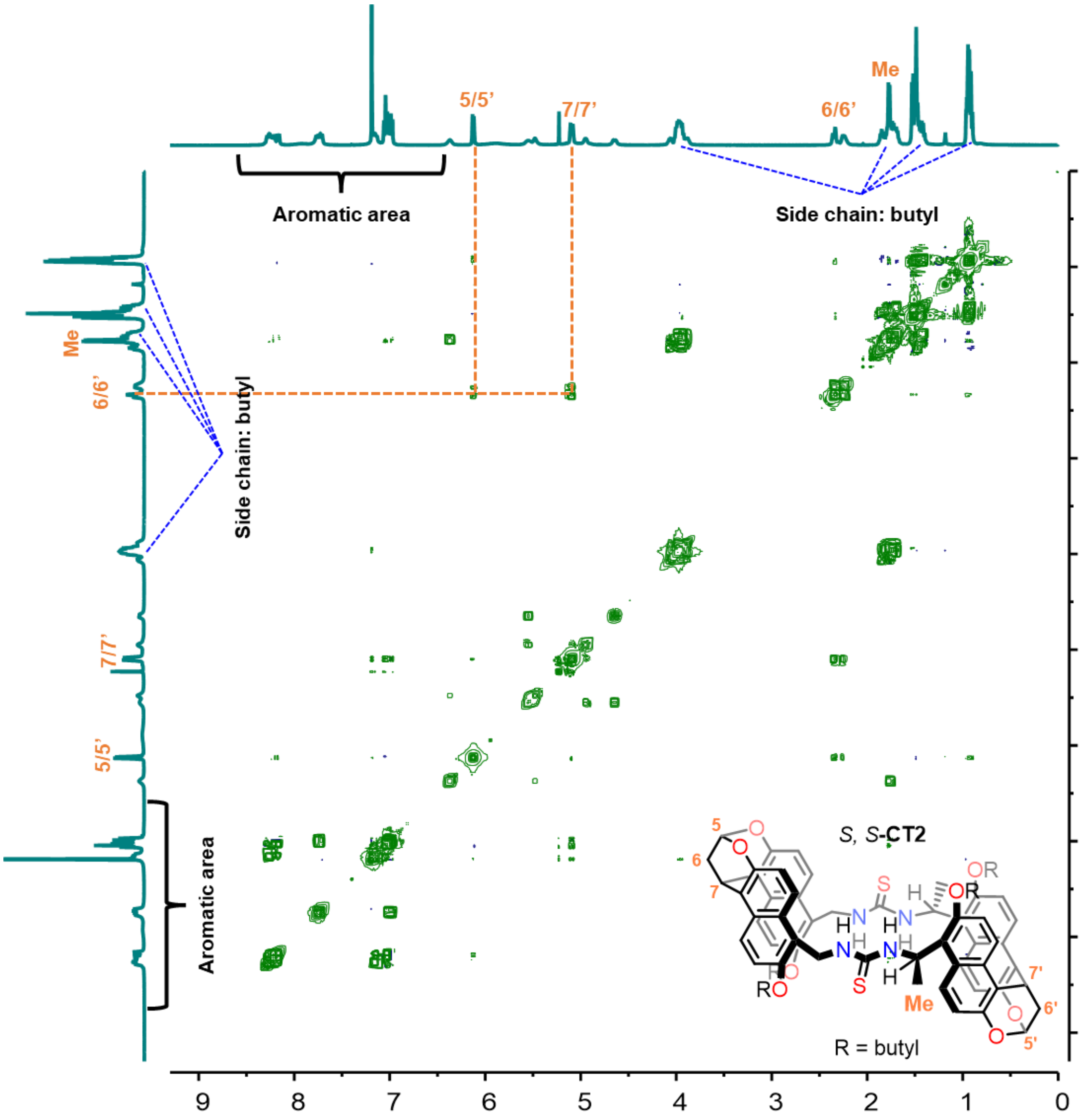

Supplementary Figure 22. 2D NMR spectra of naphthotubes. $^{1}H,^{1}H$-COSY NMR spectrum of *S,S*-**CT2** (500 MHz, $CDCl_3$, 298 K). The 2D NMR spectra of *R,R*-**CT2** are consistent with *S,S*-**CT2**.

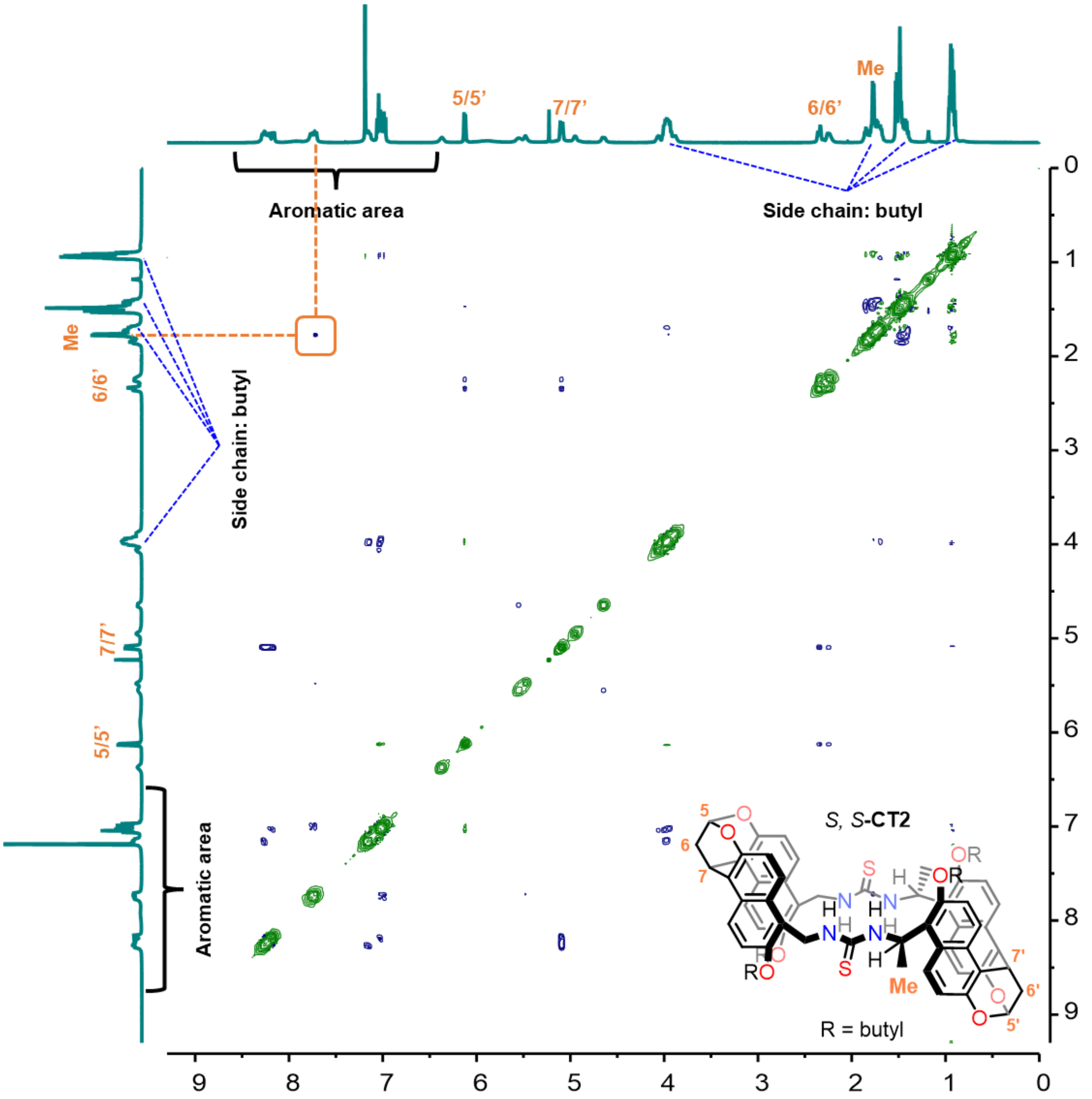

Supplementary Figure 23. 2D NMR spectra of naphthotubes. ^{1}H,^{1}H-ROESY NMR spectrum of *S,S*-**CT2** (500 MHz, $CDCl_3$, 298 K). The NOE signal between one of two methyl groups in chiral centers and aromatic protons, as shown by the orange box, indicating that the methyl group is close to the bis-naphthalene cleft. The 2D NMR spectra of *R,R*-**CT2** are consistent with *S,S*-**CT2**.

2.3. Single Crystals of Chiral Naphthotubes (anti-isomers)

R,*R*-**CT2**•2Acetone

No A or B alerts

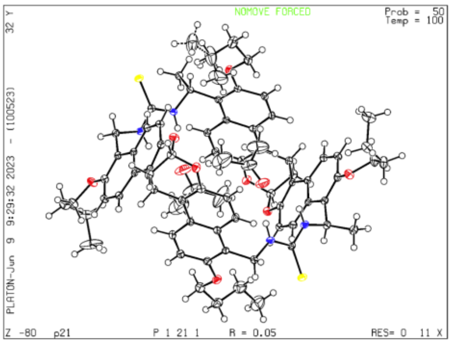

Crystal structure of *R*,*R*-**CT2**•2Acetone.

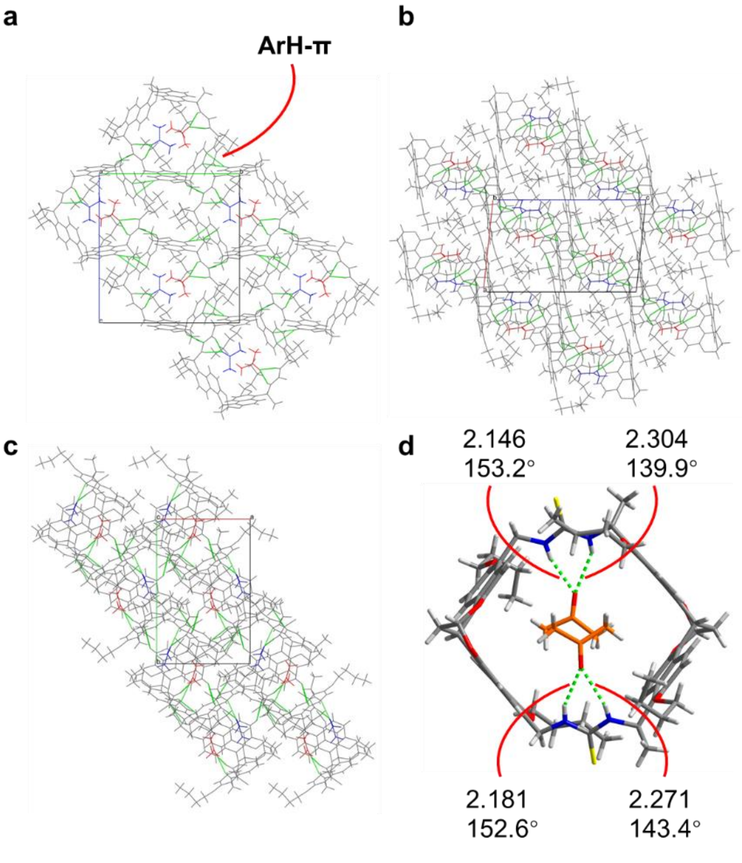

Supplementary Figure 24. Crystal data. Crystal structure of *R*,*R*-**CT2**•2Acetone. Stacking diagrams viewed along **a** a-axis; **b** b-axis; and **c** c-axis; **d** key intermolecular interactions between host and acetone. In an *R*,*R*-**CT2** molecule, two acetone molecules with different orientations form hydrogen bonds with two thiourea groups, and there are multiple CH-π (ArH-π) interactions in the crystal to achieve crystal stability. Non-covalent interactions are shown as green dashed lines.

S,S-**CT2**•2Acetone

No A or B alerts

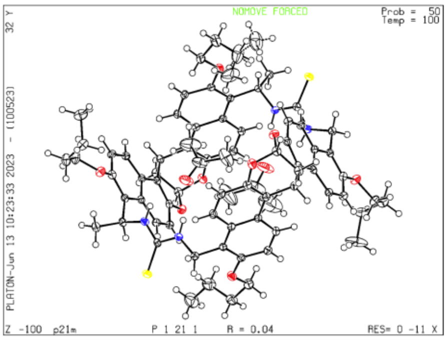

Crystal structure of *S,S*-**CT2**•2Acetone.

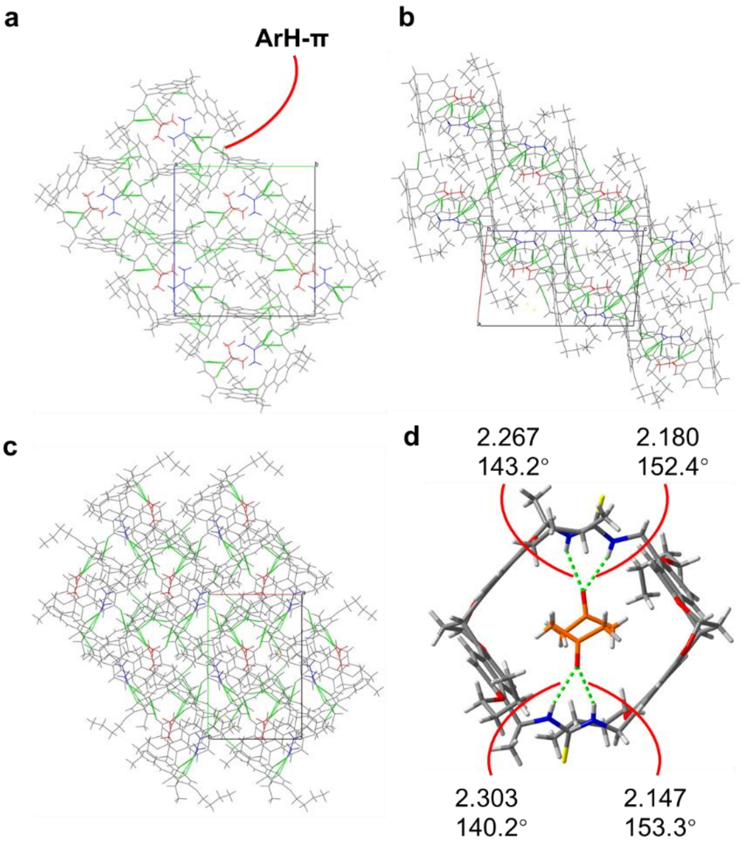

Supplementary Figure 25. Crystal data. Crystal structure of *S,S*-**CT2**•2Acetone. Stacking diagrams viewed along **a** a-axis; **b** b-axis; and **c** c-axis; **d** key intermolecular interactions between host and acetone. In an *S,S*-**CT2** molecule, two acetone molecules with different orientations form hydrogen bonds with two thiourea groups, and there are multiple CH-π (ArH-π) interactions in the crystal to achieve crystal stability. Non-covalent interactions are shown as green dashed lines.

2.4. Dihedral Angles of Bis-Naphthalene Clefts in Naphthotubes

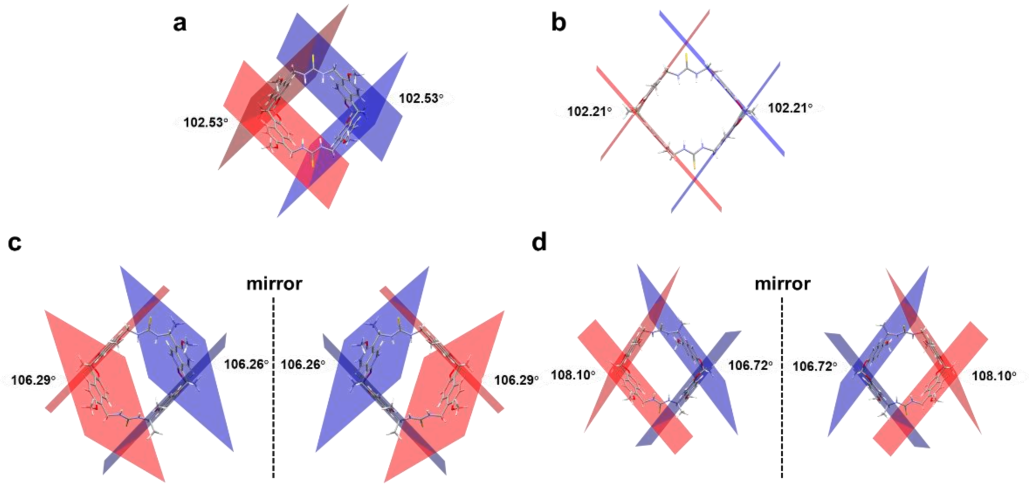

Supplementary Figure 26. Dihedral angles of bridged bis-naphthalene groups in macrocycles. a Achiral *syn*-naphthotube (102.53°, 102.53°); **b** Achiral *anti*-naphthotube (102.21°, 102.21°), **c** *R,R*-**CT1** and *S,S*-**CT1** (106.29°, 106.26°), **d** *R,R*-**CT2** and *S,S*-**CT2** (108.10°, 106.72°). All planes are calculated using 10 carbon atoms of naphthalene, butoxys are replaced with methoxys for clarity. Energy minimized structures obtained by DFT (B3LYP/6-31G* with D3)[2,3,4,5] calculations with the PCM solution model[6,7] in chloroform at 298 K.

2.5. HPLC of Chiral naphthotubes on Chiral Column

**Column :**	CHIRALPAK® IK-3 (IK30CE-CM026)
**Column size**	0.46 cm I.D. × 25 cm L × 3 μm
**Injection**	1μl Mobile
**Phase**	n-Hexane / Ethanol / Trifluoroacetic acid = 60/40/0.1(v/v/v)
**Flow rate**	1.0 mL/min
**Wavelength**	UV 220 nm
**Temperature**	35 °C
**Sample solution**	1 mg/ml in MeOH 50% DCM 50%
**HPLC equipment**	Shimadzu LC 20A QA&QC-HPLC-12

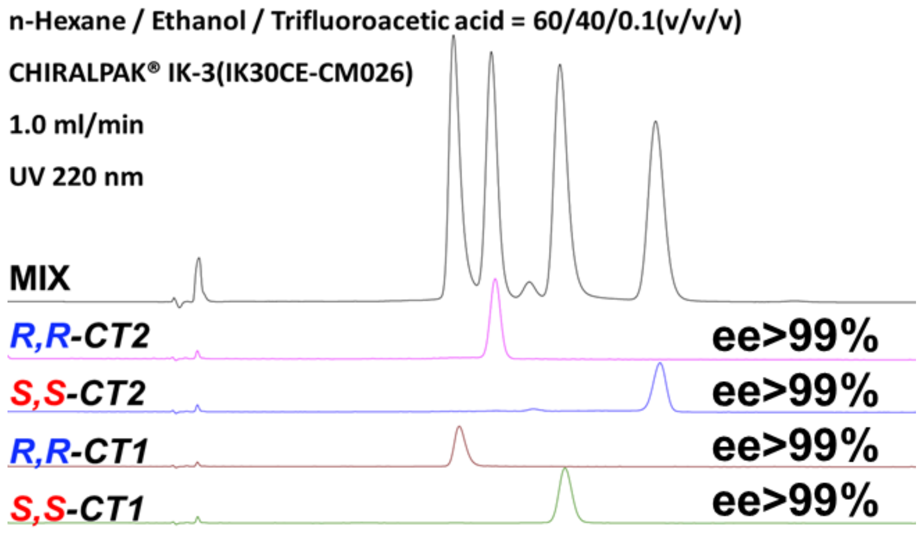

Supplementary Figure 27. Chiral HPLC stack spectra of four macrocycles (*R*,*R*-**CT1**, *S*,*S*-**CT1**, *R*,*R*-**CT2** and *S*,*S*-**CT2**).

2.6. ¹H NMR Spectra of the 1: 1 Host-Guest Complexes

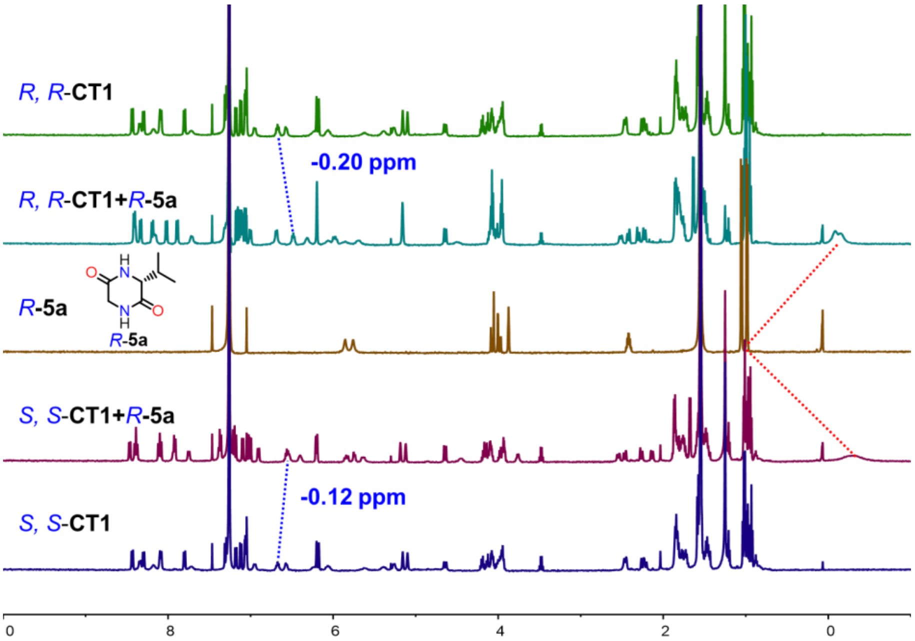

Supplementary Figure 28. **1: 1 host-guest complexes.** ¹H NMR spectra of host-guest 1: 1 complexes (500 MHz, $CDCl_3$, 0.5 mM, 298 K). The blue dashed line marks the signal change of host chemical shift, The red dashed line marks the signal change of guest chemical shift.

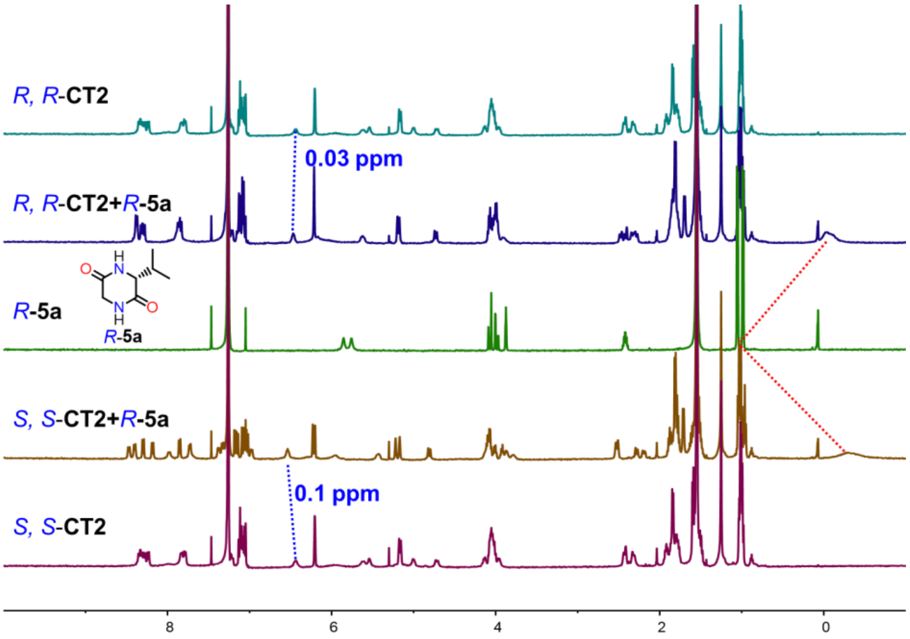

Supplementary Figure 29. **1: 1 host-guest complexes.** ¹H NMR spectra of host-guest 1: 1 complexes (500 MHz, $CDCl_3$, 0.5 mM, 298 K). The blue dashed line marks the signal change of host chemical shift, The red dashed line marks the signal change of guest chemical shift.

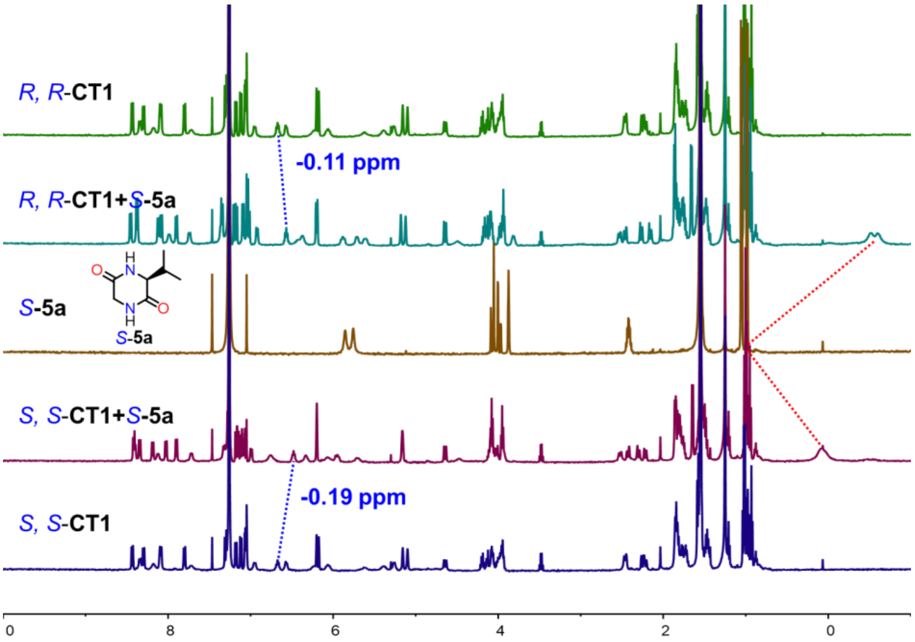

Supplementary Figure 30. 1: 1 host-guest complexes. ^{1}H NMR spectra of host-guest 1: 1 complexes (500 MHz, $CDCl_3$, 0.5 mM, 298 K). The blue dashed line marks the signal change of host chemical shift, The red dashed line marks the signal change of guest chemical shift.

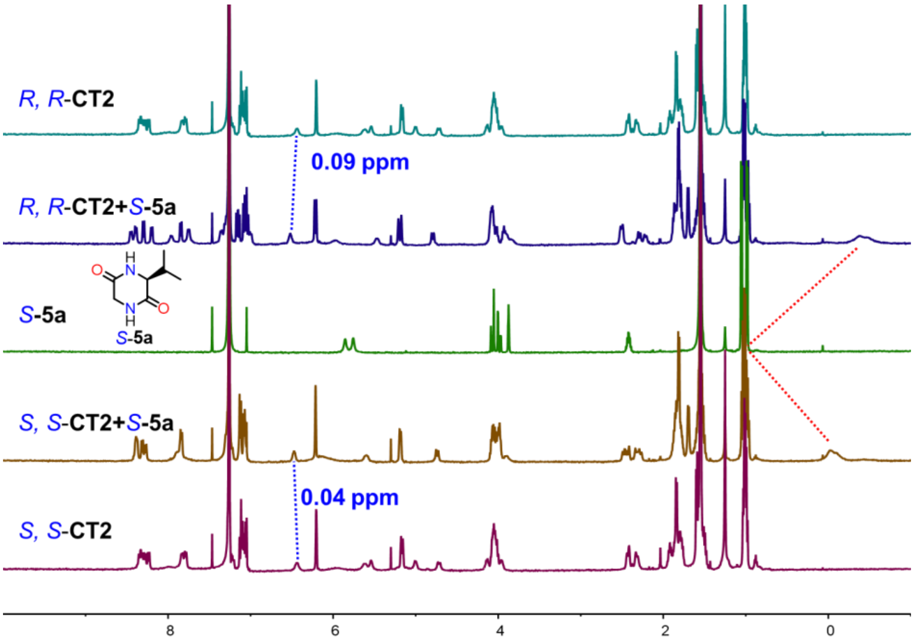

Supplementary Figure 31. 1: 1 host-guest complexes. ^{1}H NMR spectra of host-guest 1: 1 complexes (500 MHz, $CDCl_3$, 0.5 mM, 298 K). The blue dashed line marks the signal change of host chemical shift, The red dashed line marks the signal change of guest chemical shift.

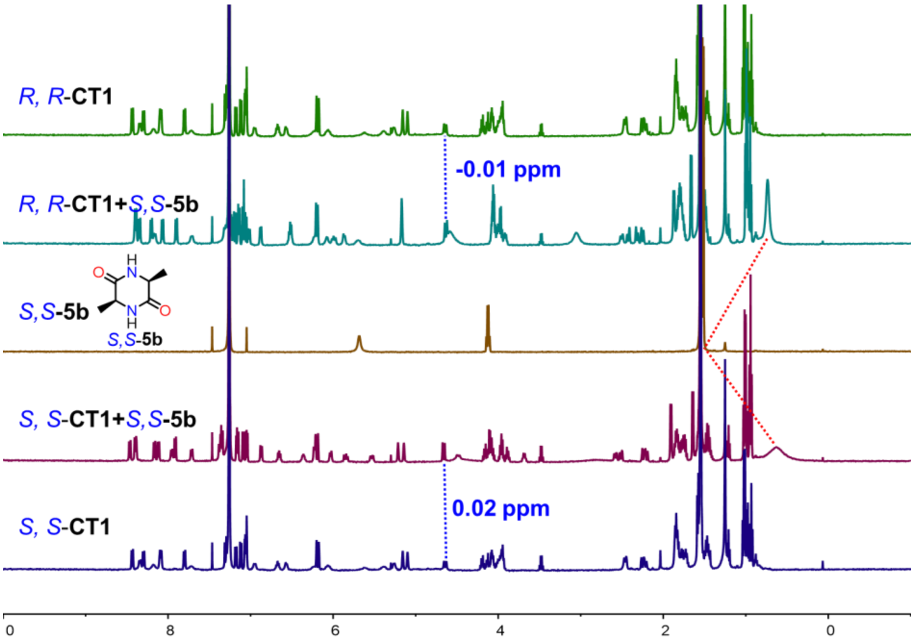

Supplementary Figure 32. **1: 1 host-guest complexes.** ^{1}H NMR spectra of host-guest 1: 1 complexes (500 MHz, $CDCl_3$, 0.5 mM, 298 K). The blue dashed line marks the signal change of host chemical shift, The red dashed line marks the signal change of guest chemical shift.

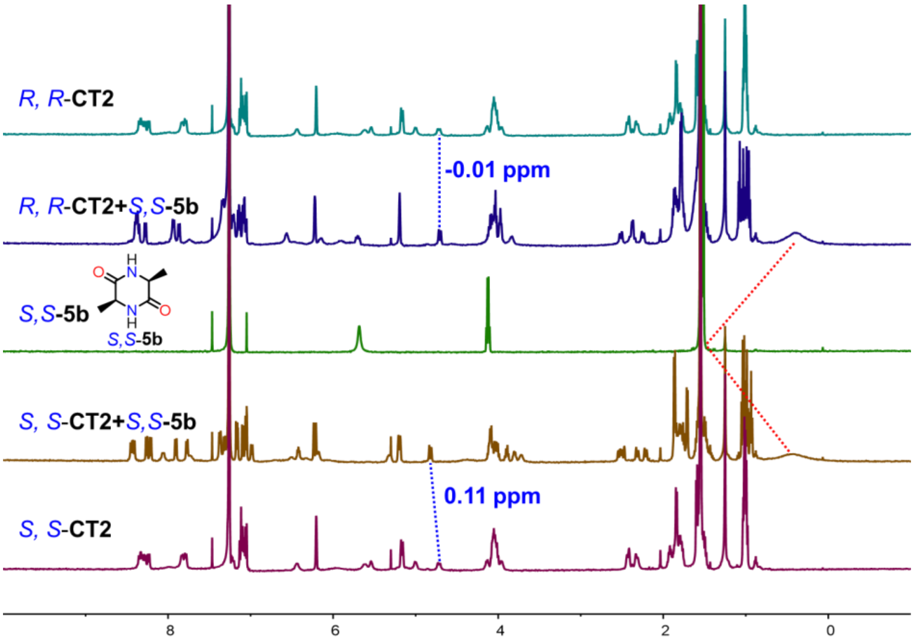

Supplementary Figure 33. **1: 1 host-guest complexes.** ^{1}H NMR spectra of host-guest 1: 1 complexes (500 MHz, $CDCl_3$, 0.5 mM, 298 K). The blue dashed line marks the signal change of host chemical shift, The red dashed line marks the signal change of guest chemical shift.

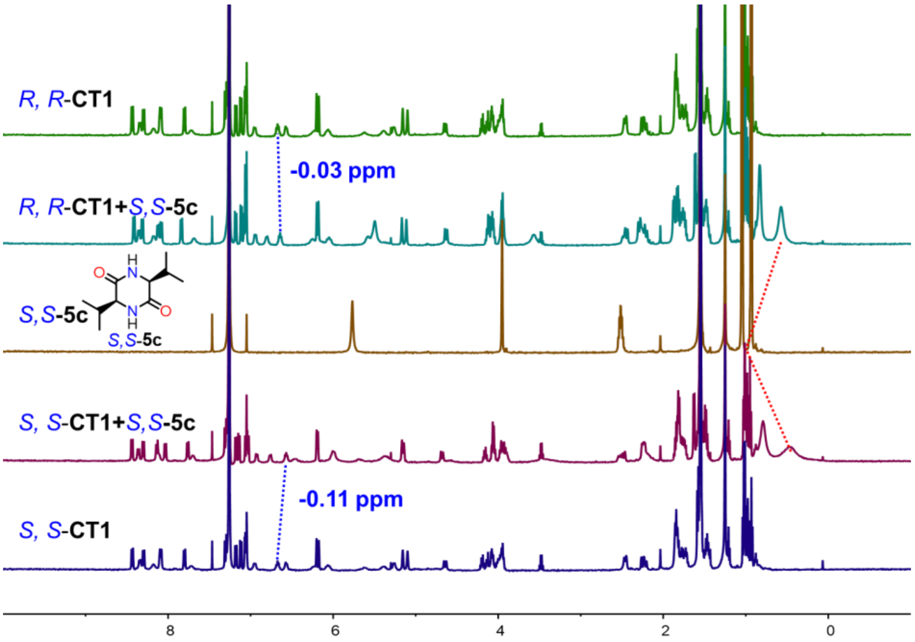

Supplementary Figure 34. **1: 1 host-guest complexes.** ^{1}H NMR spectra of host-guest 1: 1 complexes (500 MHz, $CDCl_3$, 0.5 mM, 298 K). The blue dashed line marks the signal change of host chemical shift, The red dashed line marks the signal change of guest chemical shift.

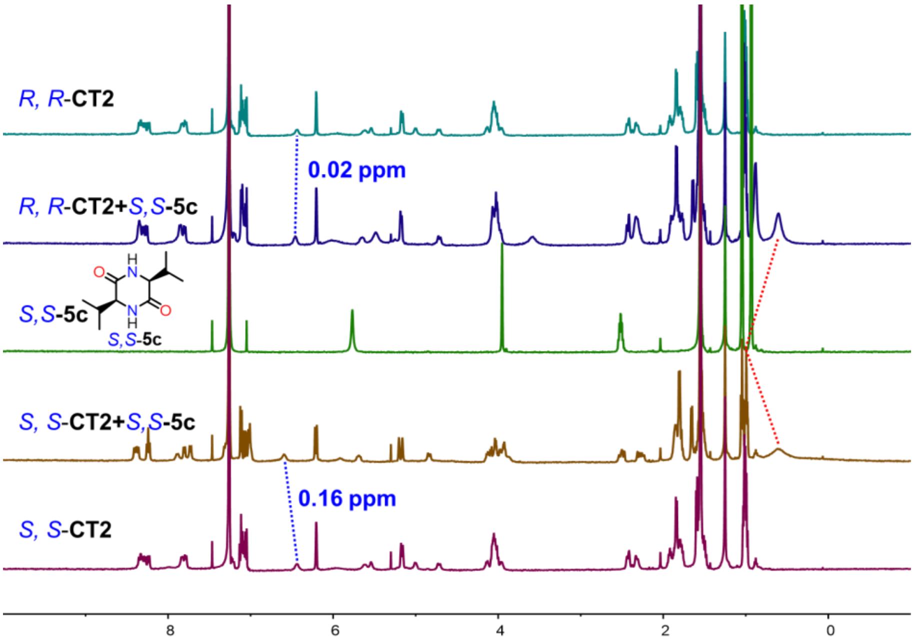

Supplementary Figure 35. **1: 1 host-guest complexes.** ^{1}H NMR spectra of host-guest 1: 1 complexes (500 MHz, $CDCl_3$, 0.5 mM, 298 K). The blue dashed line marks the signal change of host chemical shift, The red dashed line marks the signal change of guest chemical shift.

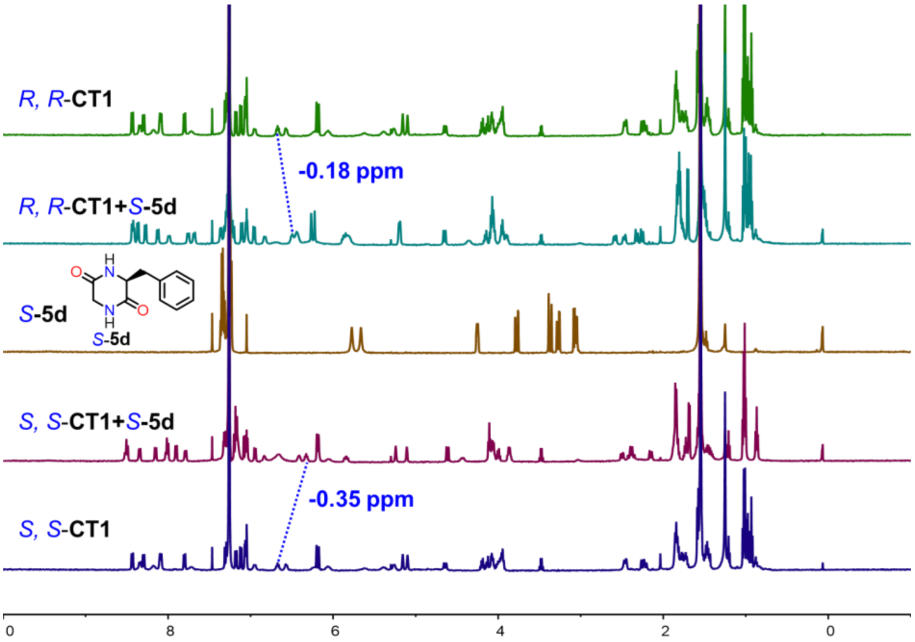

Supplementary Figure 36. **1: 1 host-guest complexes.** ^{1}H NMR spectra of host-guest 1: 1 complexes (500 MHz, $CDCl_3$, 0.5 mM, 298 K). The blue dashed line marks the signal change of host chemical shift, The red dashed line marks the signal change of guest chemical shift.

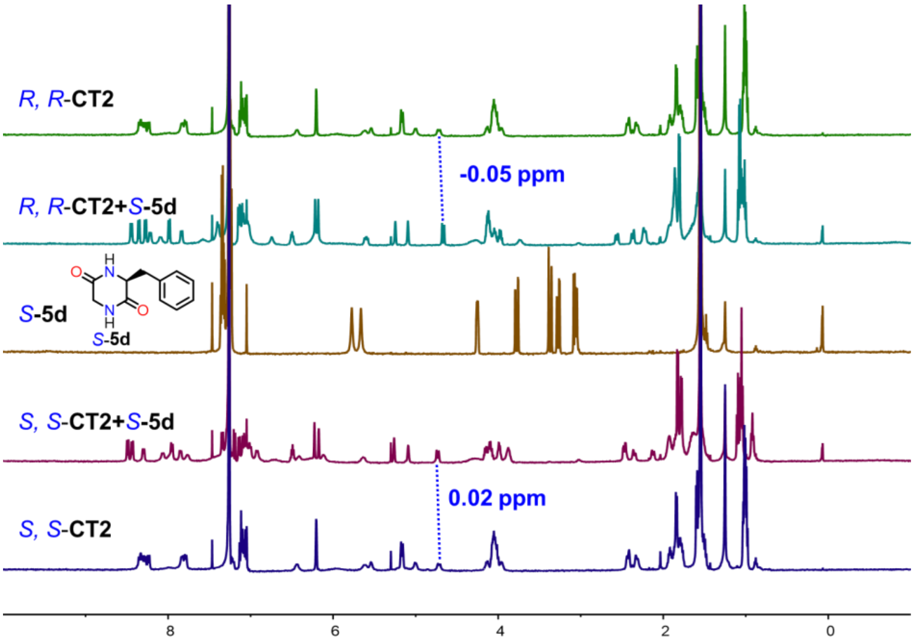

Supplementary Figure 37. **1: 1 host-guest complexes.** ^{1}H NMR spectra of host-guest 1: 1 complexes (500 MHz, $CDCl_3$, 0.5 mM, 298 K). The blue dashed line marks the signal change of the host chemical shift.

2.7. ^{1}H NMR Spectra of the 1: 1 Host-Guest Complexes in DMSO-d6

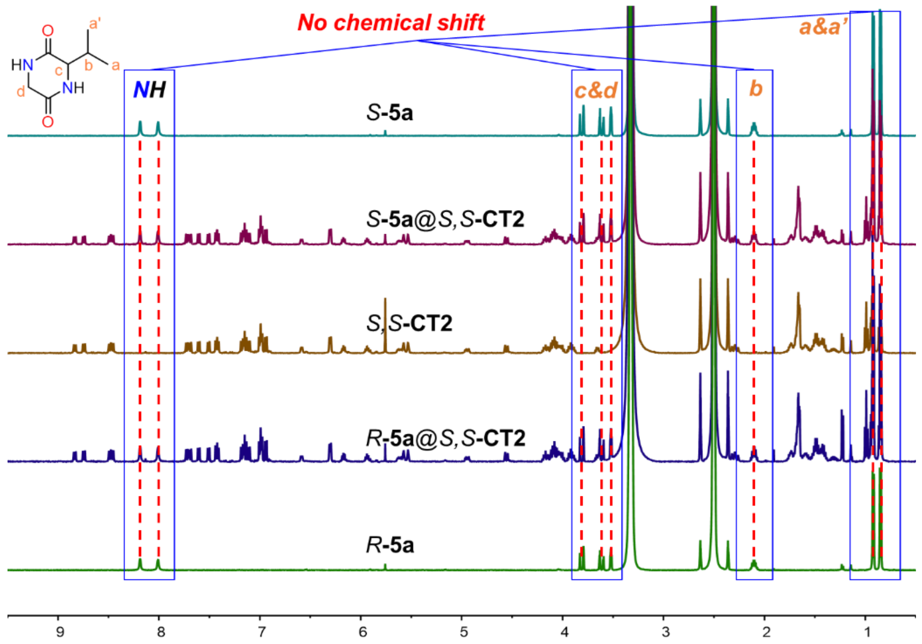

Supplementary Figure 38. **1: 1 host-guest complexes.** ^{1}H NMR spectra of host-guest 1: 1 complexes (500 MHz, DMSO-d6, 0.5 mM, 298 K). All signals of chiral guests **5a** almost have no shift after mixing with chiral host *S,S*-**CT2**, indicating that the noncovalent interactions between the guests and host were shielded by the presence of DMSO.

2.8. $^{1}H,^{1}H$-ROESY NMR spectra of *R/S*-*5a*@*S,S*-CT2

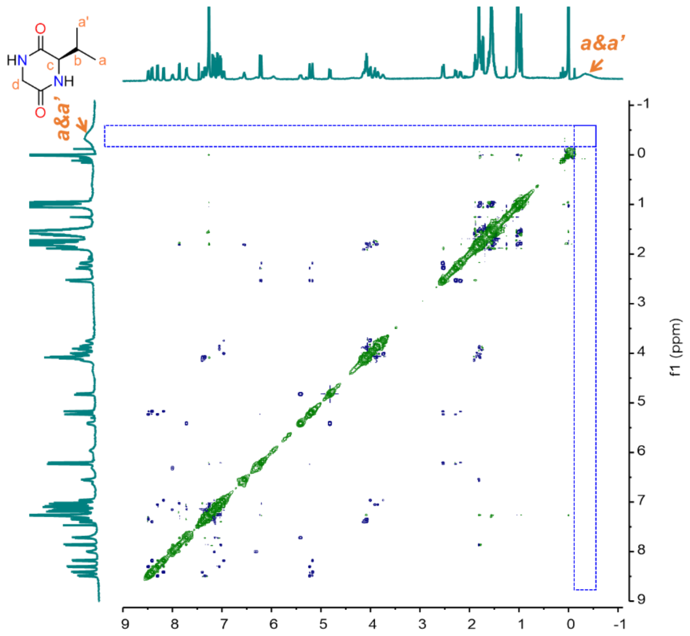

Supplementary Figure 39. **2D NMR spectra of host-guest complexes.** $^{1}H,^{1}H$-ROESY NMR spectrum of *R*-***5a***@*S,S*-**CT2** (500 MHz, $CDCl_3$, 298 K). One broad peak located at negative field can be attributed to methyl groups (**a**&**a'**) of chiral guest *R*-**5a**, however, there was no NOE signal between the methyl groups and chiral host *S,S*-**CT2**, as shown by the blue boxes.

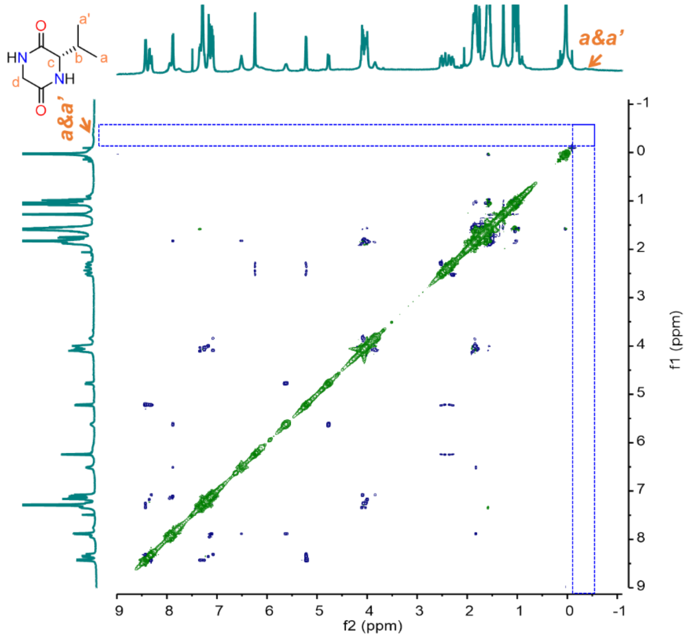

Supplementary Figure 40. **2D NMR spectra of host-guest complexes.** $^{1}H,^{1}H$-ROESY NMR spectrum of *S*-***5a***@*S,S*-**CT2** (500 MHz, $CDCl_3$, 298 K). One broad peak located at negative field can be attributed to methyl groups (**a**&**a'**) of chiral guest *S*-**5a**, however, there was no NOE signal between the methyl groups and chiral host *S,S*-**CT2**, as shown by the blue boxes.

2.9. Job Plot of Host and Guest

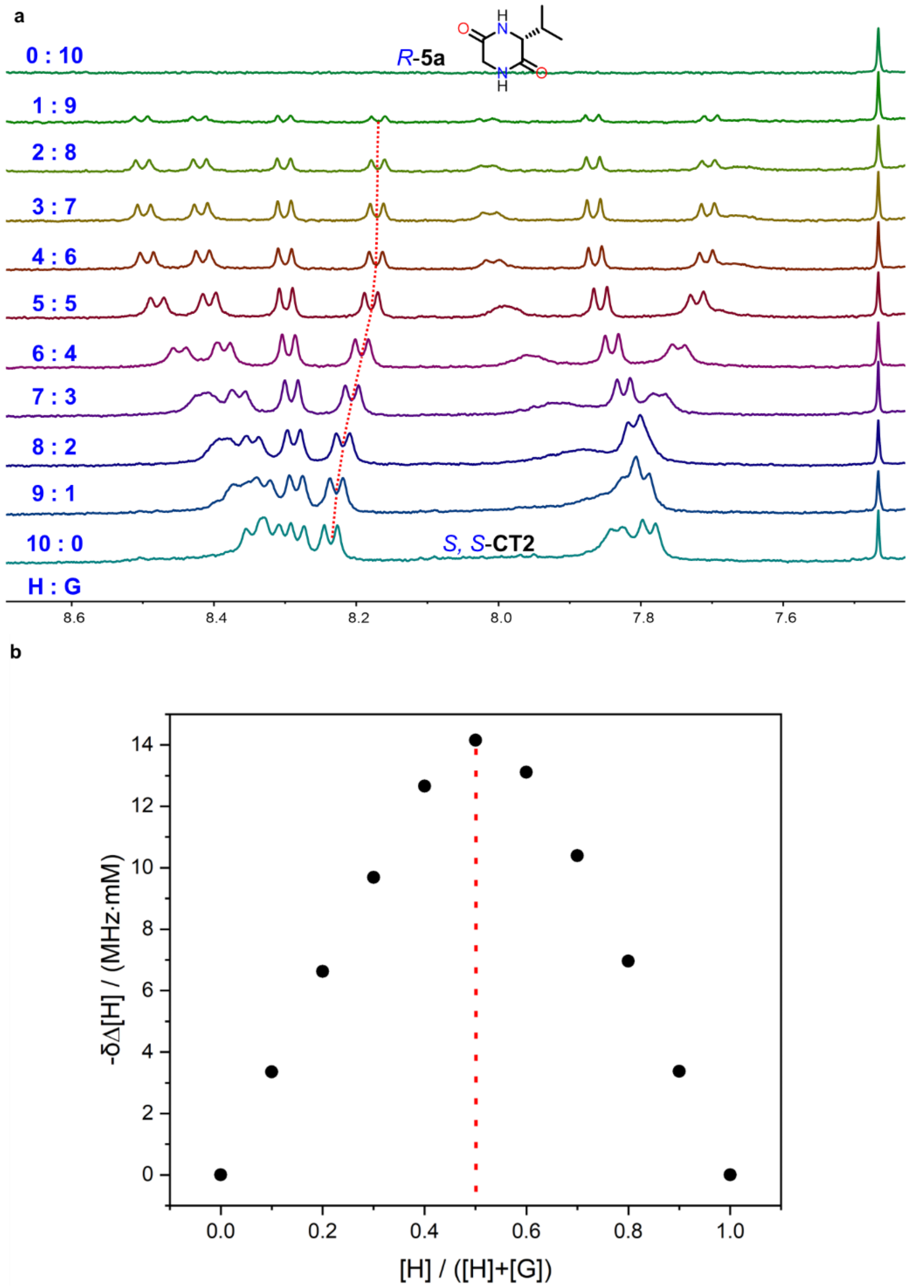

Supplementary Figure 41. **Job Plot of Host and Guest. a** Job plot was obtained by plotting the chemical shift change (Δδ) of the *S,S*-**CT2**'s proton (Ar-H) in ^{1}H NMR spectra by varying the ratio of the *S,S*-**CT2** and *R*-**5a** against the mole fraction of *S,S*-**CT2** and *R*-**5a**. The total concentration of the host and the guest is fixed: [Host] + [Guest] = 0.5 mM. **b** This experiment supports a 1: 1 binding stoichiometry between *R*-**5a** and *S,S*-**CT2** in $CDCl_3$.

2.10. ESI-HRMS for Host-Guest Complexes

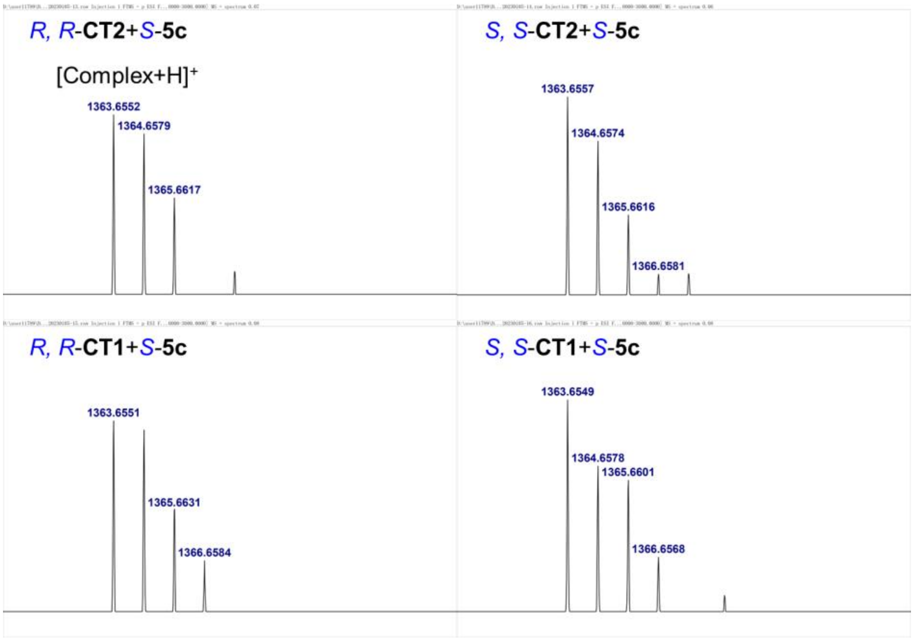

Supplementary Figure 42. ESI-HRMS data. ESI mass spectra of the solution of Hosts (*R,R*-**CT1**, *S,S*-**CT1**, *R,R*-**CT2**, *S,S*-**CT2**) in the presence of one equivalent of *S*-**5c**. The results support a 1: 1 binding stoichiometry.

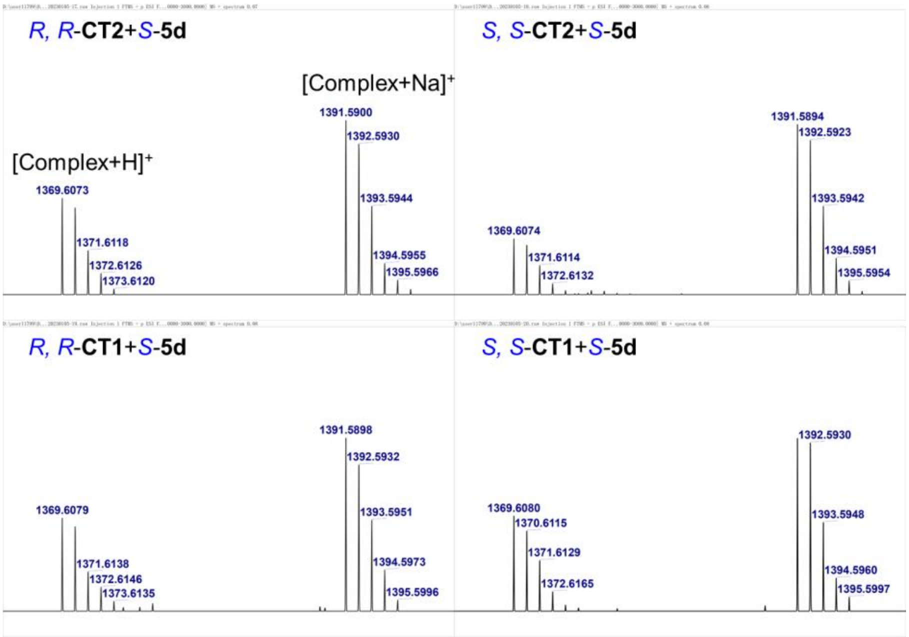

Supplementary Figure 43. ESI-HRMS data. ESI mass spectra of the solution of hosts (*R,R*-**CT1**, *S,S*-**CT1**, *R,R*-**CT2**, *S,S*-**CT2**) in the presence of one equivalent of *S*-**5d**. The results support a 1: 1 binding stoichiometry.

2.11. Determination of Association Constants of Hosts and Guests

- The association constants for the complexes of **CT**s with most guests (except *S,S*-**CT2** with *S*-**5d**) are small (< 10^5 M^{-1}) and the chemical exchange is fast at the NMR timescale. Thus, we used direct NMR titrations to determine their association constants.

- For the cases (*S,S*-**CT2** complex with *S*-**5d**) with large association constants (> 10^5 M^{-1}) and fast exchange kinetics, the binding affinities were determined by competitive NMR titrations and using guest *S*-**5a** (binding constant with *S,S*-**CT2** is 1.2×10^4 M^{-1}) as the outgoing guest. The data from competitive titrations was nonlinearly fitted[8] according to the equations developed by Prof. Werner Nau (available from their website, http://www.jacobs-university.de/ses/wnau).

a

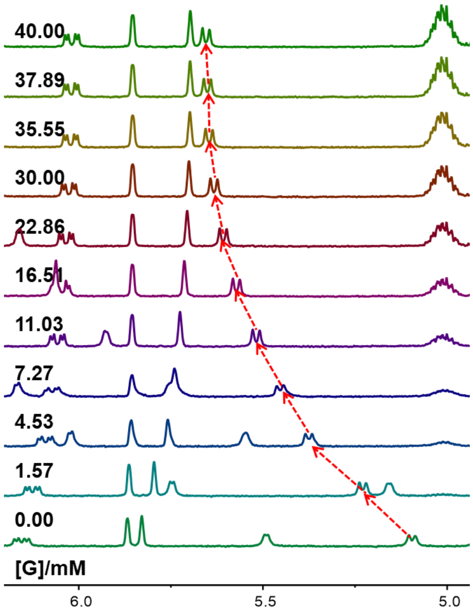

b

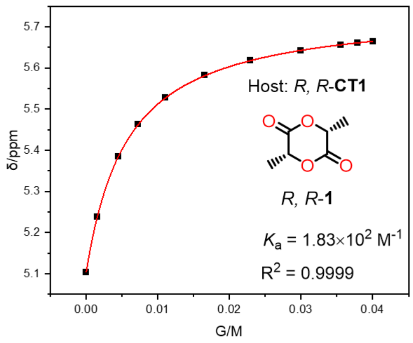

c

	K_a /M^{-1}	R^2
Titration-1	1.83×10^2	0.9999
Titration-2	1.70×10^2	0.9992
Titration-3	1.83×10^2	0.9998
Average	(1.8±0.1)×10^2	

Supplementary Figure 44. NMR titrations and association constants. **a** Partial ^{1}H NMR spectra (500 MHz, Toluene-d$_8$, 298 K) of *R*,*R*-**CT1** (0.5 mM) titrated by *R*,*R*-**1**. From bottom to top, the concentration range of 1 is 0 ~ 40.00 mM. **b** Nonlinear curve fitting for the complexation between *R*,*R*-**CT1** and *R*,*R*-**1** based on the NMR data, and **c** the averaged values and standard deviations are given in table.

a

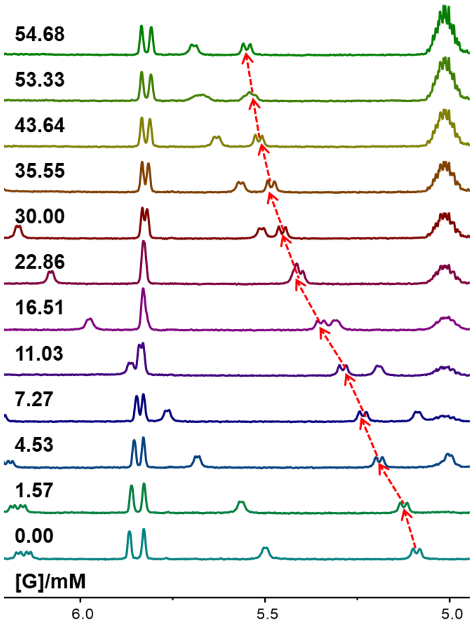

b

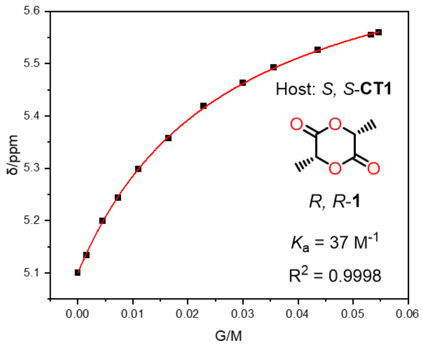

c

	K_a /M^{-1}	R^2
Titration-1	37	0.9998
Titration-2	36	0.9991
Titration-3	38	0.9994
Average	37±1	

Supplementary Figure 45. NMR titrations and association constants. **a** Partial ^{1}H NMR spectra (500 MHz, Toluene-d$_8$, 298 K) of *S*,*S*-**CT1** (0.5 mM) titrated by *R*,*R*-**1**. From bottom to top, the concentration range of 1 is 0 ~ 54.68 mM. **b** Nonlinear curve fitting for the complexation between *S*,*S*-**CT1** and *R*,*R*-**1** based on the NMR data, and **c** the averaged values and standard deviations are given in table.

a

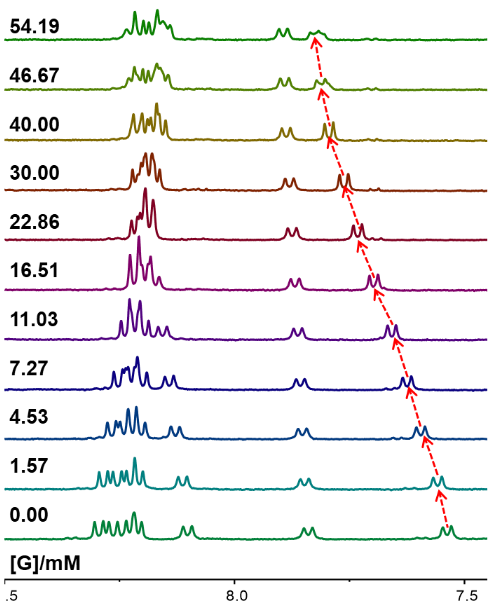

b

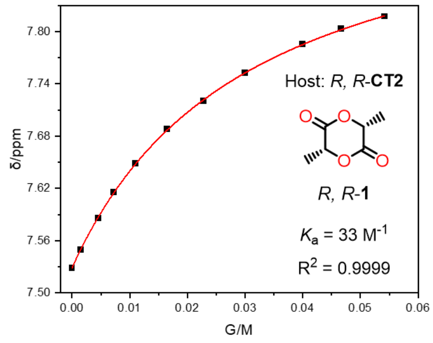

c

	K_a /M^{-1}	R^2
Titration-1	33	0.9999
Titration-2	32	0.9999
Titration-3	41	0.9991
Average	35±4	

Supplementary Figure 46. NMR titrations and association constants. a Partial ^{1}H NMR spectra (500 MHz, Toluene-d$_8$, 298 K) of *R*,*R*-**CT2** (0.5 mM) titrated by *R*,*R*-**1**. From bottom to top, the concentration range of 1 is 0 ~ 54.19 mM. **b** Nonlinear curve fitting for the complexation between *R*,*R*-**CT2** and *R*,*R*-**1** based on the NMR data, and **c** the averaged values and standard deviations are given in table.

a

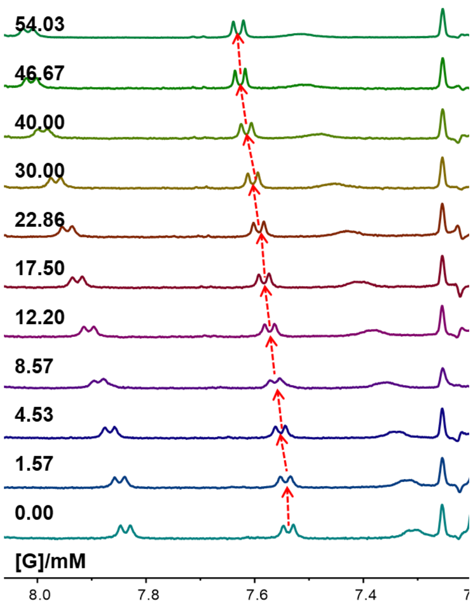

b

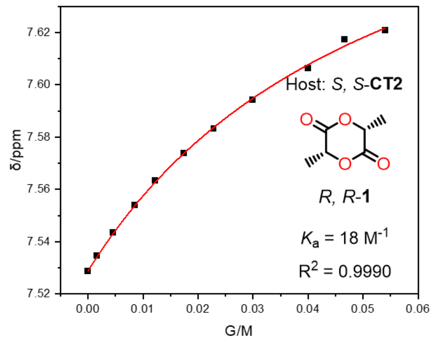

c

	K_a /M^{-1}	R^2
Titration-1	18	0.9990
Titration-2	18	0.9999
Titration-3	19	0.9998
Average	18±1	

Supplementary Figure 47. NMR titrations and association constants. a Partial ^{1}H NMR spectra (500 MHz, Toluene-d$_8$, 298 K) of *S*,*S*-**CT2** (0.5 mM) titrated by *R*,*R*-**1**. From bottom to top, the concentration range of 1 is 0 ~ 54.03 mM. **b** Nonlinear curve fitting for the complexation between *S*,*S*-**CT2** and *R*,*R*-**1** based on the NMR data, and **c** the averaged values and standard deviations are given in table.

a

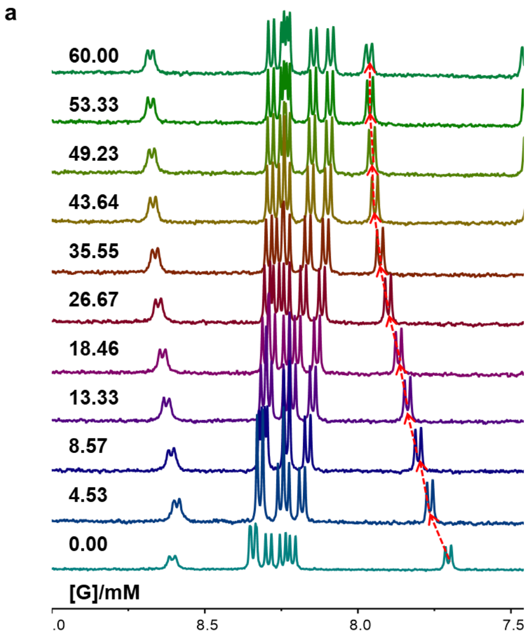

b

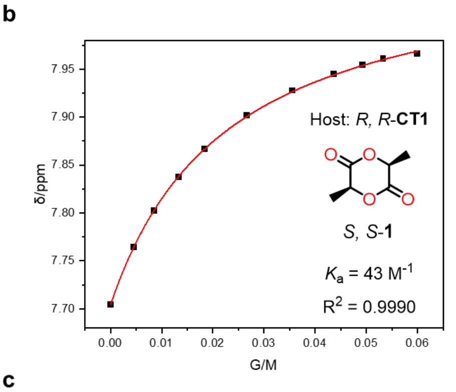

c

	K_a /M^{-1}	R^2
Titration-1	44	0.9998
Titration-2	43	0.9990
Titration-3	45	0.9998
Average	41±1	

Supplementary Figure 48. NMR titrations and association constants. a Partial ^{1}H NMR spectra (500 MHz, Toluene-d$_8$, 298 K) of *R*,*R*-**CT2**(0.5 mM) titrated by *R*,*R*-**1**. From bottom to top, the concentration range of 1 is 0 ~ 60.00 mM. **b** Nonlinear curve fitting for the complexation between *R*,*R*-**CT2** and *R*,*R*-**1** based on the NMR data, and **c** the averaged values and standard deviations are given in table.

a

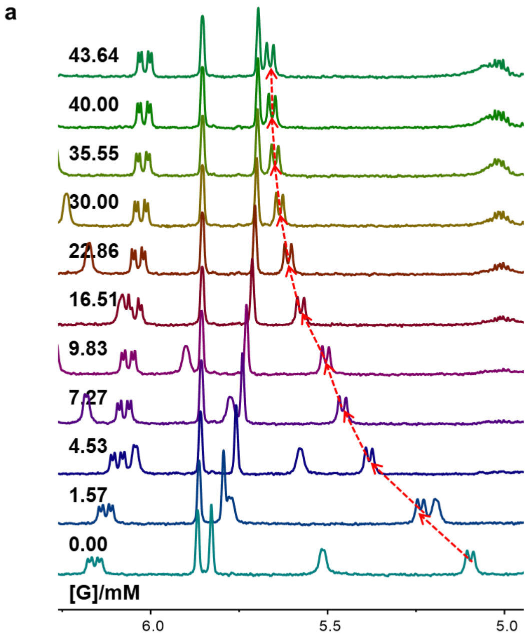

b

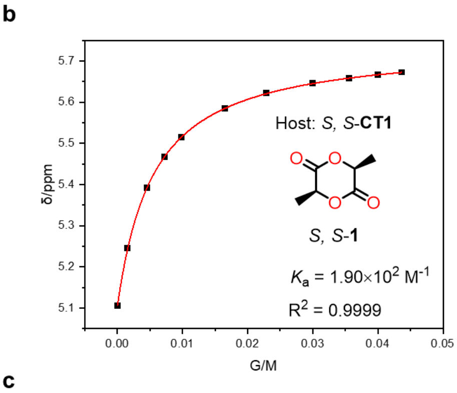

c

	K_a /M^{-1}	R^2
Titration-1	1.90×10^2	0.9999
Titration-2	1.76×10^2	0.9990
Titration-3	2.00×10^2	0.9998
Average	(1.9±0.1)×10^2	

Supplementary Figure 49. NMR titrations and association constants. a Partial ^{1}H NMR spectra (500 MHz, Toluene-d$_8$, 298 K) of *S*,*S*-**CT2** (0.5 mM) titrated by *R*,*R*-**1**. From bottom to top, the concentration range of 1 is 0 ~ 43.64 mM. **b** Nonlinear curve fitting for the complexation between *S*,*S*-**CT2** and *R*,*R*-**1** based on the NMR data, and **c** the averaged values and standard deviations are given in table.

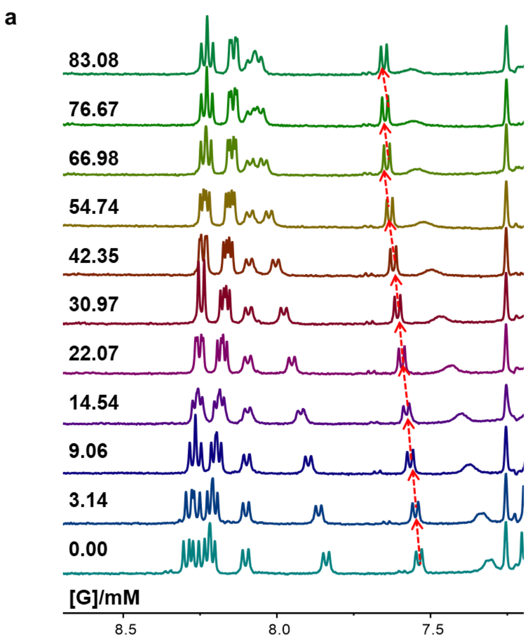

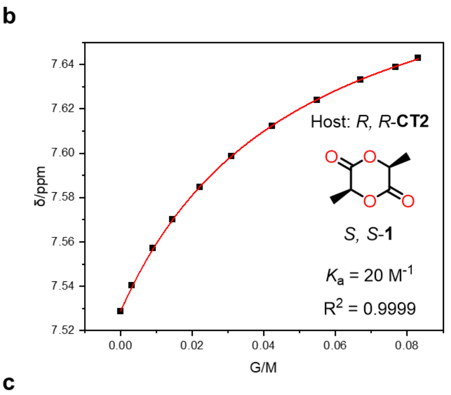

c

	K_a /M^{-1}	R^2
Titration-1	20	0.9999
Titration-2	21	0.9996
Titration-3	23	0.9998
Average	21±1	

Supplementary Figure 50. NMR titrations and association constants. a Partial ^{1}H NMR spectra (500 MHz, Toluene-d$_8$, 298 K) of *R,R*-**CT1** (0.5 mM) titrated by *R,R*-**1**. From bottom to top, the concentration range of 1 is 0 ~ 83.03 mM. **b** Nonlinear curve fitting for the complexation between *R,R*-**CT1** and *R,R*-**1** based on the NMR data, and **c** the averaged values and standard deviations are given in table.

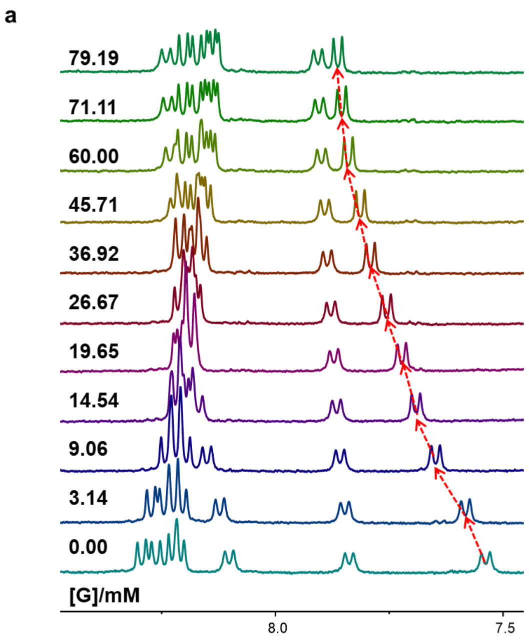

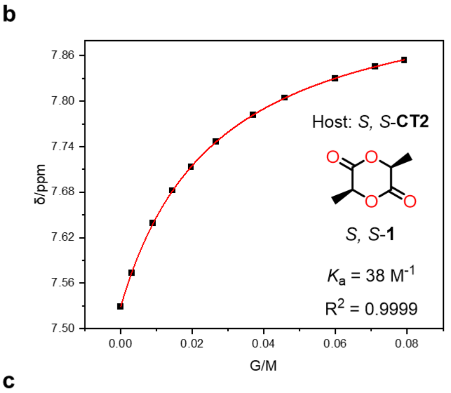

c

	K_a /M^{-1}	R^2
Titration-1	38	0.9999
Titration-2	42	0.9997
Titration-2	43	0.9979
Average	41±2	

Supplementary Figure 51. NMR titrations and association constants. a Partial ^{1}H NMR spectra (500 MHz, Toluene-d$_8$, 298 K) of *S,S*-**CT1** (0.5 mM) titrated by *R,R*-**1**. From bottom to top, the concentration range of 1 is 0 ~ 79.19 mM. **b** Nonlinear curve fitting for the complexation between *S,S*-**CT1** and *R,R*-**1** based on the NMR data, and **c** the averaged values and standard deviations are given in table.

a

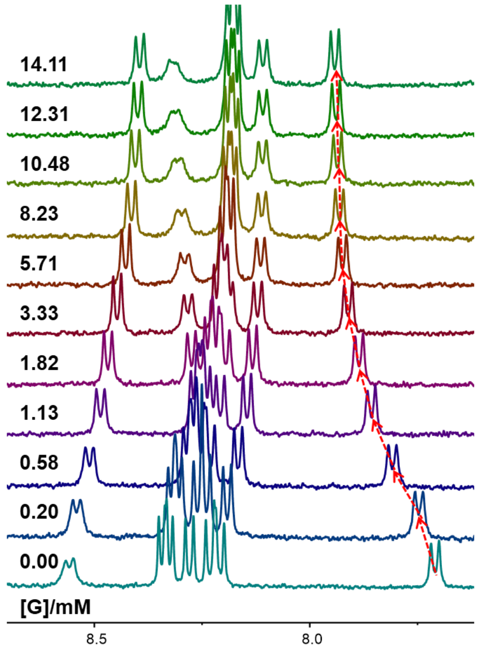

b

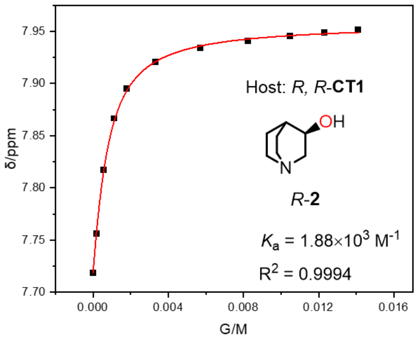

c

	K_a /M^{-1}	R^2
Titration-1	1.88×10^3	0.9994
Titration-2	1.67×10^3	0.9996
Titration-3	1.77×10^3	0.9991
Average	(1.7±0.1)×10^3	

Supplementary Figure 52. NMR titrations and association constants. a Partial ^{1}H NMR spectra (500 MHz, Toluene-d$_8$, 298 K) of *R,R*-**CT1** (0.5 mM) titrated by *R*-**2**. From bottom to top, the concentration range of 1 is 0 ~ 14.11 mM. **b** Nonlinear curve fitting for the complexation between *R,R*-**CT1** and *R*-**2** based on the NMR data, and **c** the averaged values and standard deviations are given in table.

a

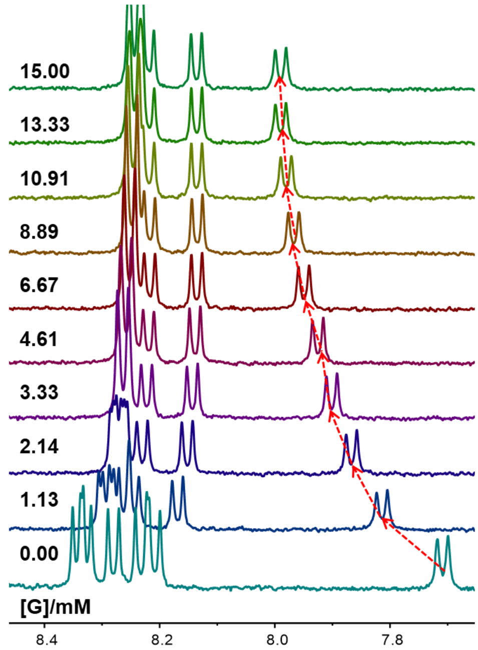

b

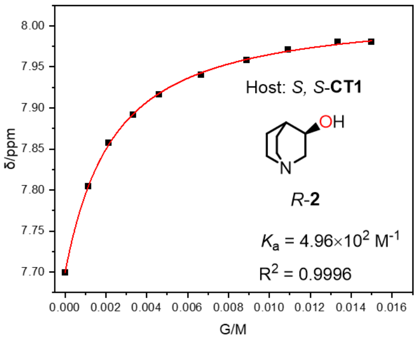

c

	K_a /M^{-1}	R^2
Titration-1	4.96×10^2	0.9996
Titration-2	4.93×10^2	0.9994
Titration-3	5.21×10^2	0.9995
Average	(5.0±0.1)×10^2	

Supplementary Figure 53. NMR titrations and association constants. a Partial ^{1}H NMR spectra (500 MHz, Toluene-d$_8$, 298 K) of *S,S*-**CT1** (0.5 mM) titrated by *R*-**2**. From bottom to top, the concentration range of 1 is 0 ~ 16.00 mM. **b** Nonlinear curve fitting for the complexation between *S,S*-**CT1** and *R*-**2** based on the NMR data, and **c** the averaged values and standard deviations are given in table.

a

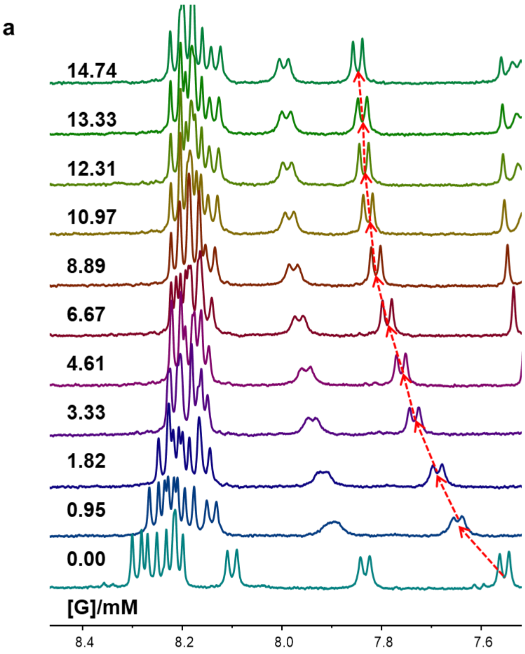

b

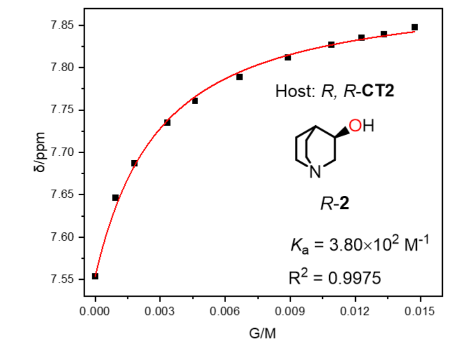

c

	K_a /M^{-1}	R^2
Titration-1	3.80×10^2	0.9975
Titration-2	3.42×10^2	0.9972
Titration-3	3.46×10^2	0.9968
Average	(3.6±0.2)×10^2	

Supplementary Figure 54. NMR titrations and association constants. a Partial ^{1}H NMR spectra (500 MHz, Toluene-d$_8$, 298 K) of *R*,*R*-**CT2** (0.5 mM) titrated by *R*-**2**. From bottom to top, the concentration range of 1 is 0 ~ 14.74 mM. **b** Nonlinear curve fitting for the complexation between *R*,*R*-**CT2** and *R*-**2** based on the NMR data, and **c** the averaged values and standard deviations are given in table.

a

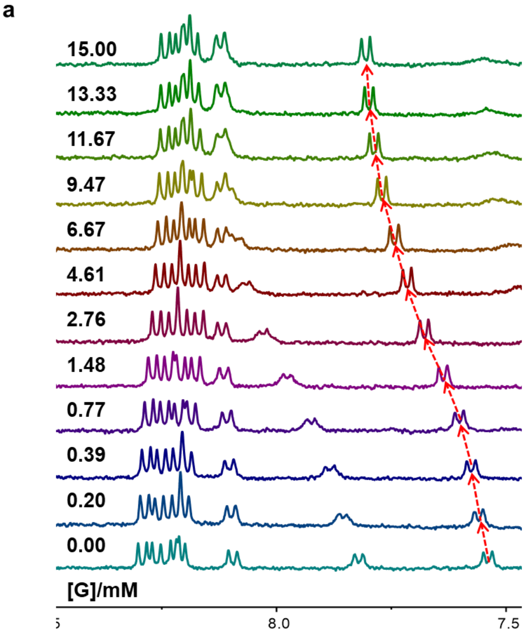

b

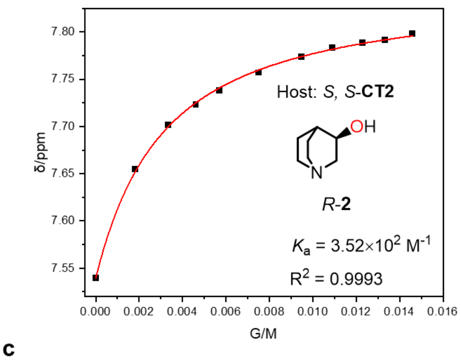

c

	K_a /M^{-1}	R^2
Titration-1	3.52×10^2	0.9993
Titration-2	2.99×10^2	0.9977
Titration-3	3.04×10^2	0.9980
Average	(3.2±0.2)×10^2	

Supplementary Figure 55. NMR titrations and association constants. a Partial ^{1}H NMR spectra (500 MHz, Toluene-d$_8$, 298 K) of *S*,*S*-**CT2** (0.5 mM) titrated by *R*-**2**. From bottom to top, the concentration range of 1 is 0 ~ 15.00 mM. **b** Nonlinear curve fitting for the complexation between *S*,*S*-**CT2** and *R*-**2** based on the NMR data, and **c** the averaged values and standard deviations are given in table.

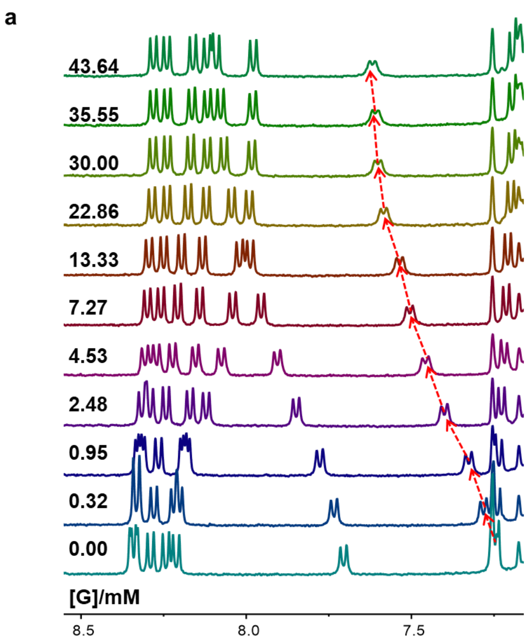

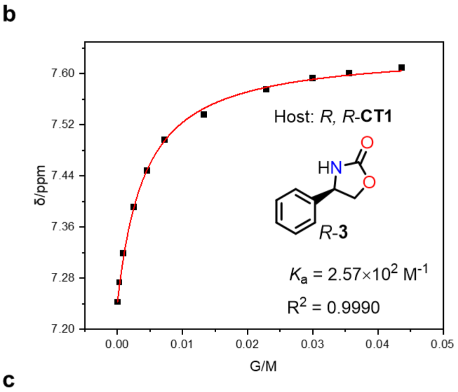

c

	K_a /M^{-1}	R^2
Titration-1	2.57×10^2	0.9990
Titration-2	2.18×10^2	0.9990
Titration-3	2.11×10^2	0.9993
Average	(2.3±0.2)×10^2	

Supplementary Figure 56. NMR titrations and association constants. a Partial ^{1}H NMR spectra (500 MHz, Toluene-d$_8$, 298 K) of *R,R*-**CT1** (0.5 mM) titrated by *R*-**3**. From bottom to top, the concentration range of 1 is 0 ~ 43.64 mM. **b** Nonlinear curve fitting for the complexation between *R,R*-**CT1** and *R*-**3** based on the NMR data, and **c** the averaged values and standard deviations are given in table.

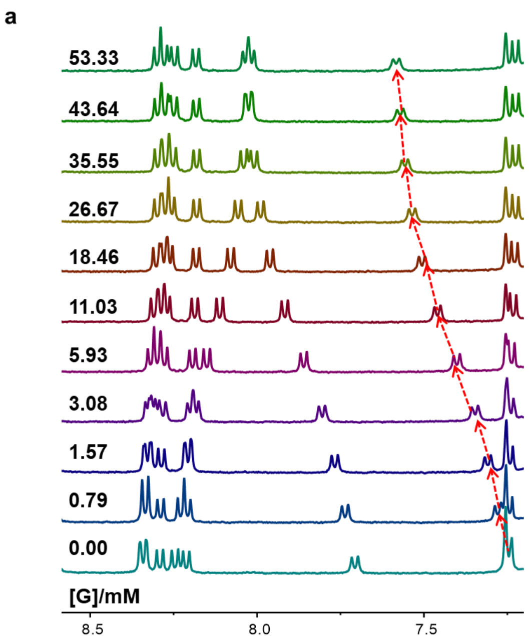

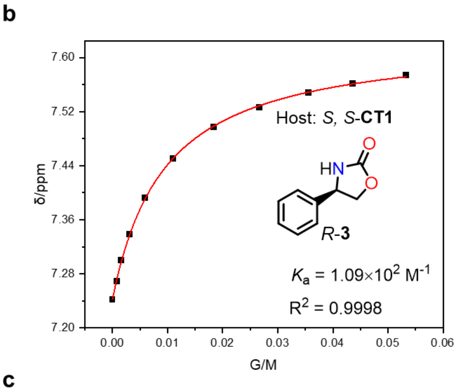

c

	K_a /M^{-1}	R^2
Titration-1	1.09×10^2	0.9998
Titration-2	1.06×10^2	0.9986
Titration-3	1.28×10^2	0.991
Average	(1.1±0.1)×10^2	

Supplementary Figure 57. NMR titrations and association constants. a Partial ^{1}H NMR spectra (500 MHz, Toluene-d$_8$, 298 K) of *S,S*-**CT1** (0.5 mM) titrated by *R*-**3**. From bottom to top, the concentration range of 1 is 0 ~ 53.33 mM. **b** Nonlinear curve fitting for the complexation between *S,S*-**CT1** and *R*-**3** based on the NMR data, and **c** the averaged values and standard deviations are given in table.

a

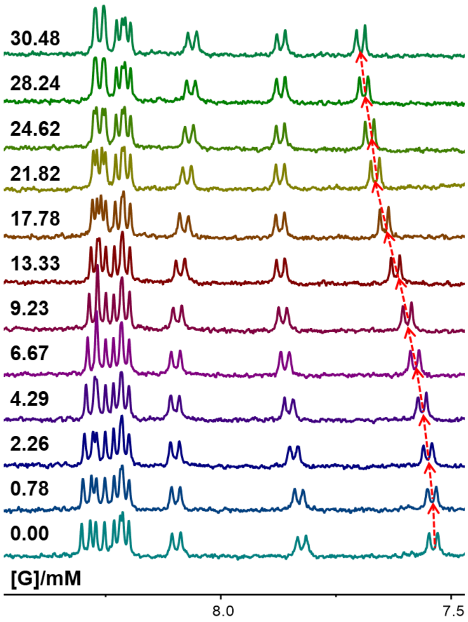

b

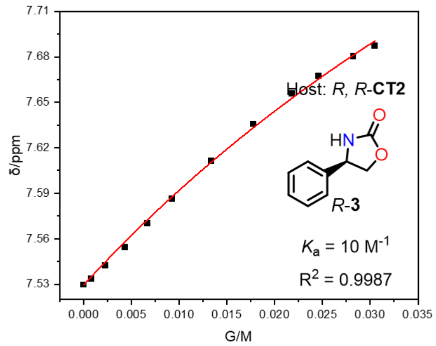

c

	K_a /M^{-1}	R^2
Titration-1	10	0.9987
Titration-2	9	0.9982
Titration-3	10	0.9983
Average	9±1	

Supplementary Figure 58. NMR titrations and association constants. **a** Partial ^{1}H NMR spectra (500 MHz, Toluene-d$_8$, 298 K) of *R*,*R*-**CT2** (0.5 mM) titrated by *R*-**3**. From bottom to top, the concentration range of 1 is 0 ~ 30.48 mM. **b** Nonlinear curve fitting for the complexation between *R*,*R*-**CT2** and *R*-**3** based on the NMR data, and **c** the averaged values and standard deviations are given in table.

a

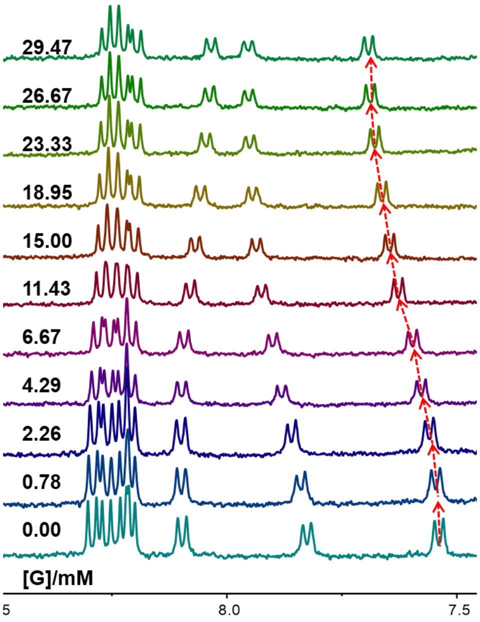

b

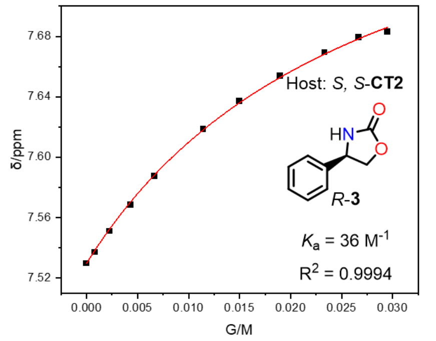

c

	K_a /M^{-1}	R^2
Titration-1	36	0.9994
Titration-2	37	0.9994
Titration-3	35	0.9995
Average	36±1	

Supplementary Figure 59. NMR titrations and association constants. **a** Partial ^{1}H NMR spectra (500 MHz, Toluene-d$_8$, 298 K) of *S*,*S*-**CT2** (0.5 mM) titrated by *R*-**3**. From bottom to top, the concentration range of 1 is 0 ~ 29.47 mM. **b** Nonlinear curve fitting for the complexation between *S*,*S*-**CT2** and *R*-**3** based on the NMR data, and **c** the averaged values and standard deviations are given in table.

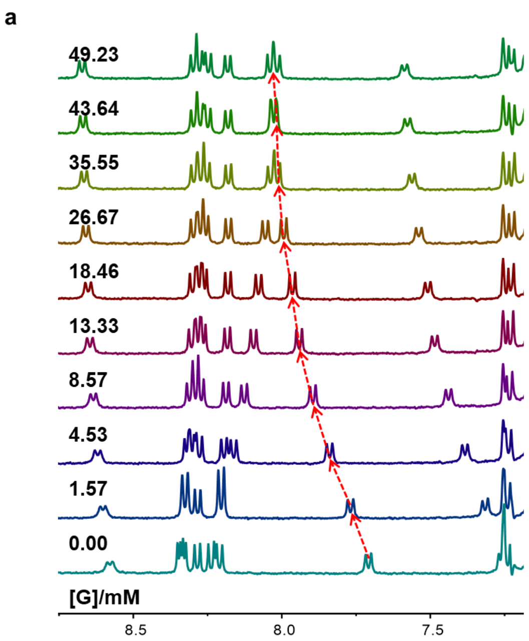

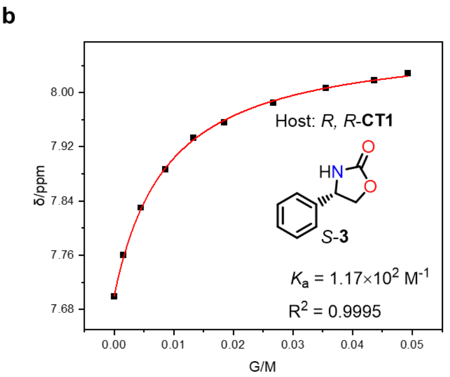

c

	K_a /M^{-1}	R^2
Titration-1	1.17×10^2	0.9995
Titration-2	1.11×10^2	0.9993
Titration-3	1.06×10^2	0.9997
Average	(1.1±0.1)×10^2	

Supplementary Figure 60. NMR titrations and association constants. a Partial ^{1}H NMR spectra (500 MHz, Toluene-d$_8$, 298 K) of *R,R*-**CT1** (0.5 mM) titrated by *S*-**3**. From bottom to top, the concentration range of 1 is 0 ~ 49.23 mM. **b** Nonlinear curve fitting for the complexation between *R,R*-**CT1** and *S*-**3** based on the NMR data, and **c** the averaged values and standard deviations are given in table.

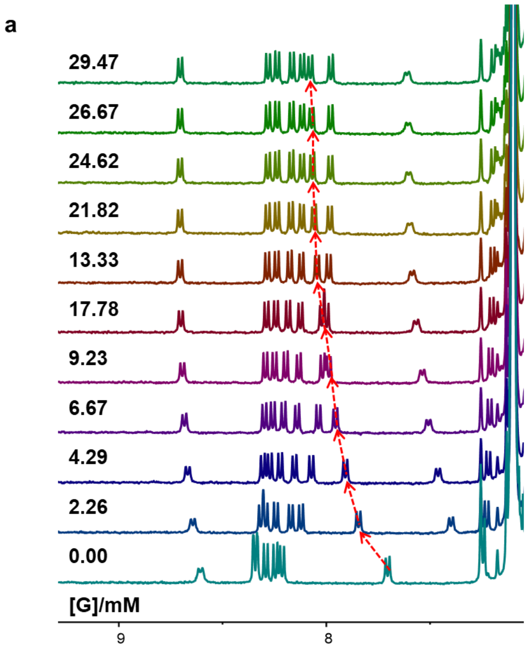

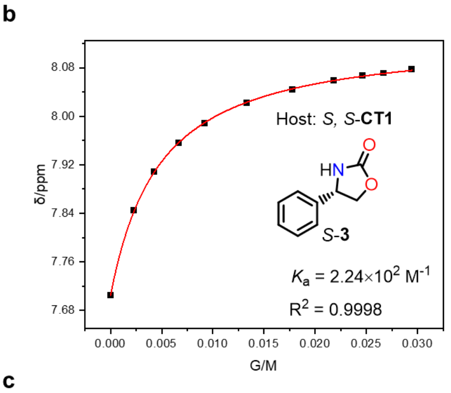

c

	K_a /M^{-1}	R^2
Titration-1	2.24×10^2	0.9998
Titration-2	2.47×10^2	0.9996
Titration-3	2.26×10^2	0.9998
Average	(2.3±0.1)×10^2	

Supplementary Figure 61. NMR titrations and association constants. a Partial ^{1}H NMR spectra (500 MHz, Toluene-d$_8$, 298 K) of *S,S*-**CT1** (0.5 mM) titrated by *S*-**3**. From bottom to top, the concentration range of 1 is 0 ~ 29.47 mM. **b** Nonlinear curve fitting for the complexation between *S,S*-**CT1** and *S*-**3** based on the NMR data, and **c** the averaged values and standard deviations are given in table.

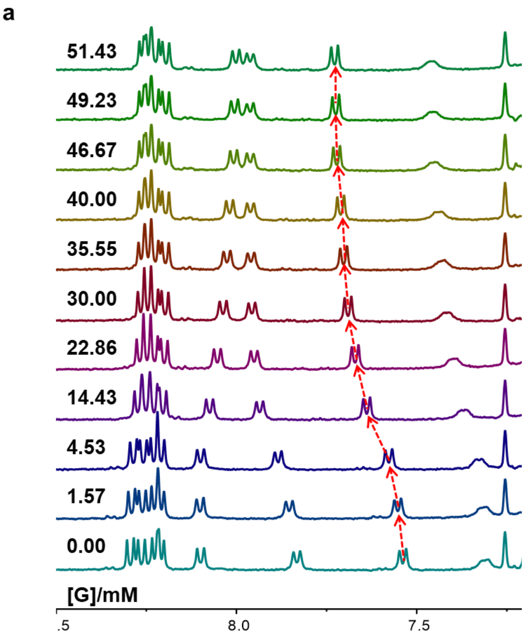

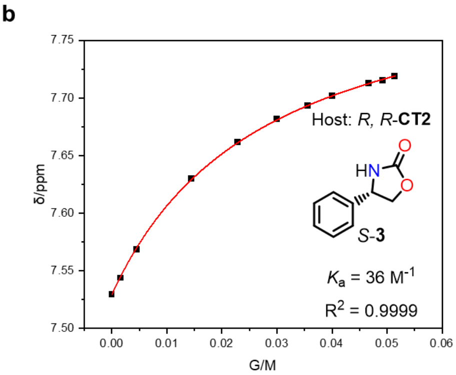

c

	K_a /M^{-1}	R^2
Titration-1	36	0.9999
Titration-2	37	0.9999
Titration-3	33	0.9998
Average	35±2	

Supplementary Figure 62. NMR titrations and association constants. a Partial ^{1}H NMR spectra (500 MHz, Toluene-d$_8$, 298 K) of *R,R*-**CT2** (0.5 mM) titrated by *S*-**3**. From bottom to top, the concentration range of 1 is 0 ~ 51.43 mM. **b** Nonlinear curve fitting for the complexation between *R,R*-**CT2** and *S*-**3** based on the NMR data, and **c** the averaged values and standard deviations are given in table.

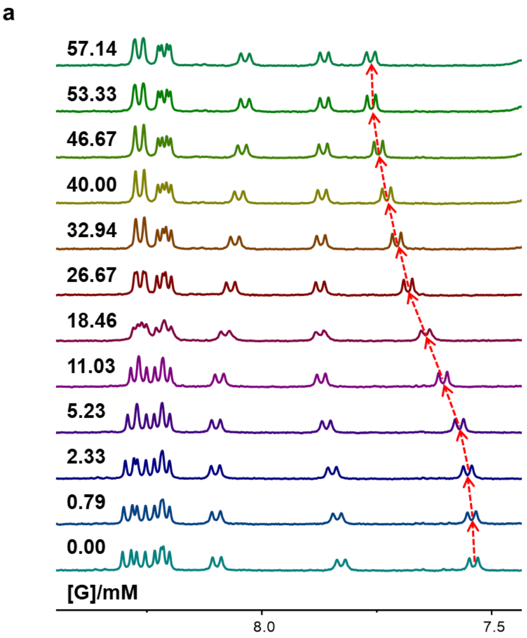

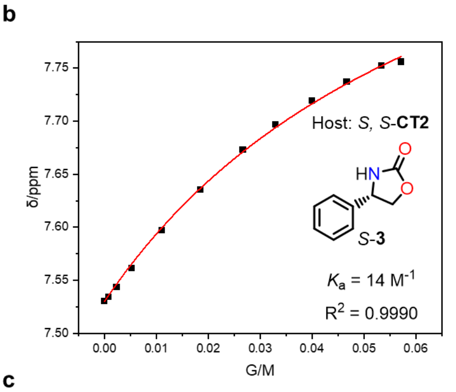

c

	K_a /M^{-1}	R^2
Titration-1	14	0.9990
Titration-2	13	0.9973
Titration-3	13	0.9994
Average	13±1	

Supplementary Figure 63. NMR titrations and association constants. a Partial ^{1}H NMR spectra (500 MHz, Toluene-d$_8$, 298 K) of *S,S*-**CT2** (0.5 mM) titrated by *S*-**3**. From bottom to top, the concentration range of 1 is 0 ~ 57.14 mM. **b** Nonlinear curve fitting for the complexation between *S,S*-**CT2** and *S*-**3** based on the NMR data, and **c** the averaged values and standard deviations are given in table.

a

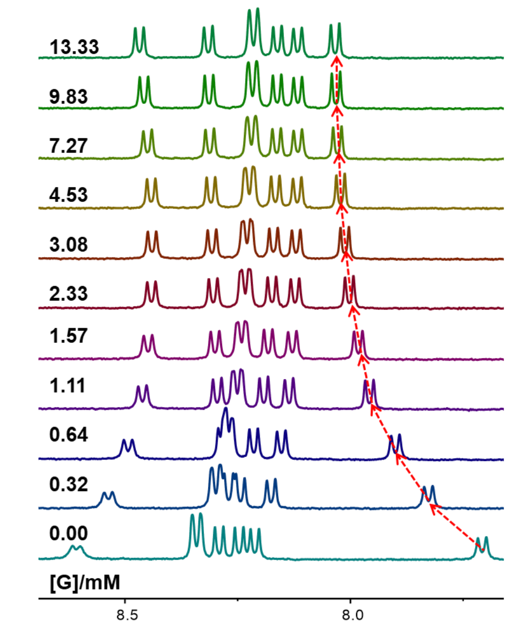

b

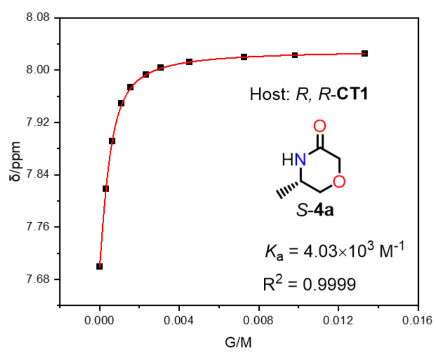

c

	K_a /M^{-1}	R^2
Titration-1	4.03×10^3	0.9999
Titration-2	3.79×10^3	0.9998
Titration-3	4.43×10^3	0.9997
Average	(4.1±0.3)×10^3	

Supplementary Figure 64. NMR titrations and association constants. a Partial ^{1}H NMR spectra (500 MHz, Toluene-d$_8$, 298 K) of *R,R*-**CT1** (0.5 mM) titrated by *S*-**4a**. From bottom to top, the concentration range of 1 is 0 ~ 13.33 mM. **b** Nonlinear curve fitting for the complexation between *R,R*-**CT1** and *S*-**4a** based on the NMR data, and **c** the averaged values and standard deviations are given in table.

a

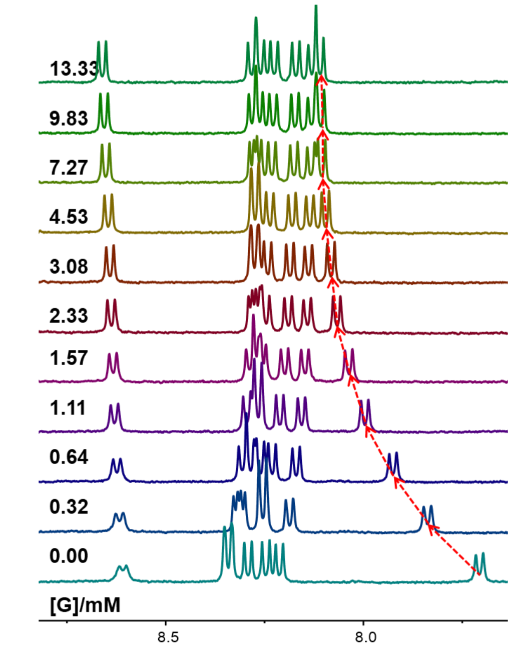

b

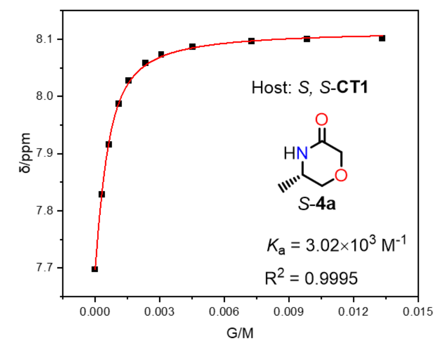

c

	K_a /M^{-1}	R^2
Titration-1	3.02×10^3	0.9995
Titration-2	3.29×10^3	0.9994
Titration-3	3.36×10^3	0.9997
Average	(3.2±0.1)×10^3	

Supplementary Figure 65. NMR titrations and association constants. a Partial ^{1}H NMR spectra (500 MHz, Toluene-d$_8$, 298 K) of *S,S*-**CT1** (0.5 mM) titrated by *S*-**4a**. From bottom to top, the concentration range of 1 is 0 ~ 1.33 mM. **b** Nonlinear curve fitting for the complexation between *S,S*-**CT1** and *S*-**4a** based on the NMR data, and **c** the averaged values and standard deviations are given in table.

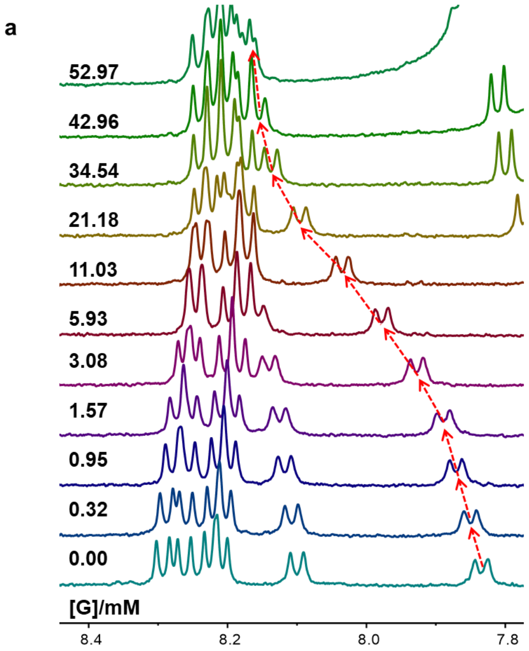

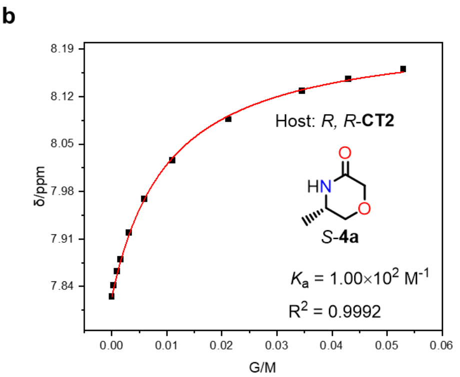

c

	K_a /M^{-1}	R^2
Titration-1	1.00×10^2	0.9992
Titration-2	0.83×10^2	0.9998
Titration-3	0.80×10^2	0.9998
Average	90±1	

Supplementary Figure 66. NMR titrations and association constants. a Partial ^{1}H NMR spectra (500 MHz, Toluene-d$_8$, 298 K) of *R,R*-**CT2** (0.5 mM) titrated by *S*-**4a**. From bottom to top, the concentration range of 1 is 0 ~ 52.97 mM. **b** Nonlinear curve fitting for the complexation between *R,R*-**CT2** and *S*-**4a** based on the NMR data, and **c** the averaged values and standard deviations are given in table.

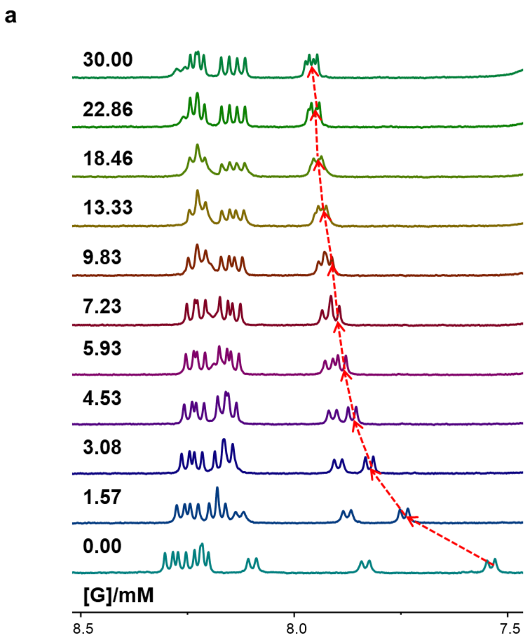

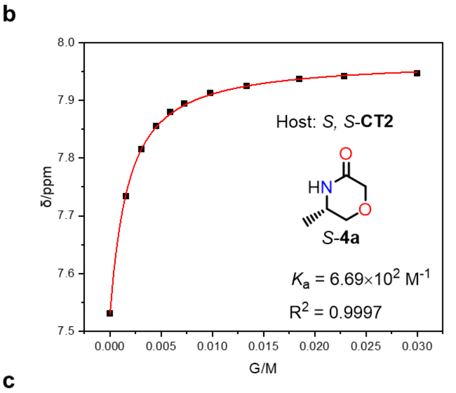

c

	K_a /M^{-1}	R^2
Titration-1	6.69×10^2	0.9997
Titration-2	6.67×10^2	0.9993
Titration-3	6.54×10^2	0.9998
Average	(6.6±0.1)×10^2	

Supplementary Figure 67. NMR titrations and association constants. a Partial ^{1}H NMR spectra (500 MHz, Toluene-d$_8$, 298 K) of *S,S*-**CT2** (0.5 mM) titrated by *S*-**4a**. From bottom to top, the concentration range of 1 is 0 ~ 30.00 mM. **b** Nonlinear curve fitting for the complexation between *S,S*-**CT2** and *S*-**4a** based on the NMR data, and **c** the averaged values and standard deviations are given in table.

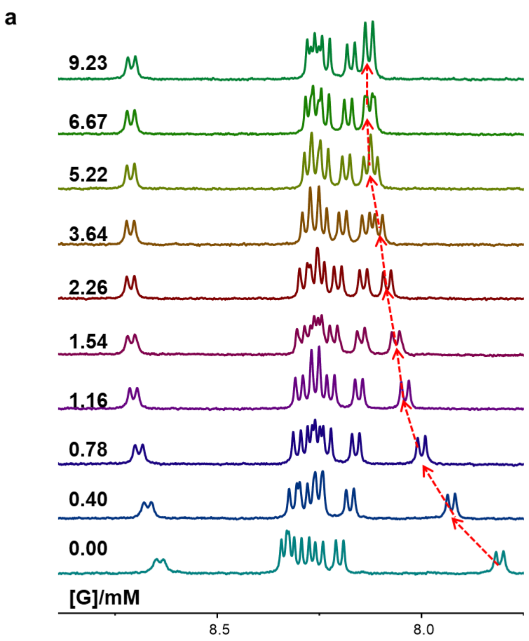

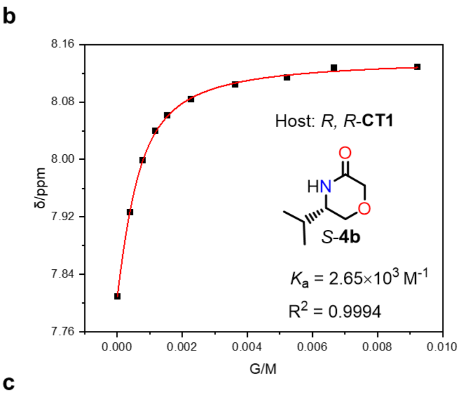

c

	K_a /M^{-1}	R^2
Titration-1	2.65×10^3	0.9994
Titration-2	3.01×10^3	0.9998
Titration-3	2.88×10^3	0.9986
Average	(2.8±0.1)×10^3	

Supplementary Figure 68. NMR titrations and association constants. a Partial ^{1}H NMR spectra (500 MHz, Toluene-d$_8$, 298 K) of *R,R*-**CT1** (0.5 mM) titrated by *S*-**4b**. From bottom to top, the concentration range of 1 is 0 ~ 9.23 mM. **b** Nonlinear curve fitting for the complexation between *R,R*-**CT1** and *S*-**4b** based on the NMR data, and **c** the averaged values and standard deviations are given in table.

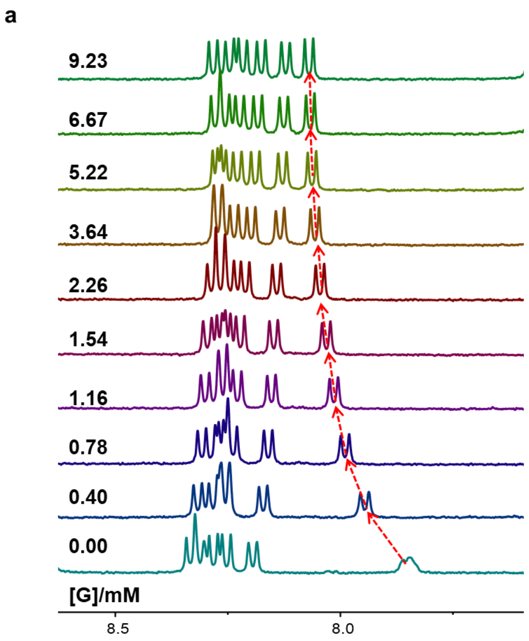

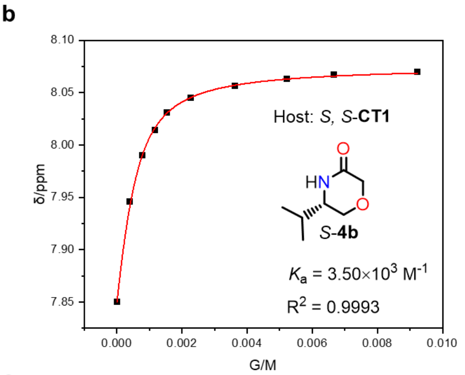

c

	K_a /M^{-1}	R^2
Titration-1	3.50×10^3	0.9993
Titration-2	2.81×10^3	0.9994
Titration-3	3.13×10^3	0.9998
Average	(3.1±0.3)×10^3	

Supplementary Figure 69. NMR titrations and association constants. a Partial ^{1}H NMR spectra (500 MHz, Toluene-d$_8$, 298 K) of *S,S*-**CT1** (0.5 mM) titrated by *S*-**4b**. From bottom to top, the concentration range of 1 is 0 ~ 9.23 mM. **b** Nonlinear curve fitting for the complexation between *S,S*-**CT1** and *S*-**4b** based on the NMR data, and **c** the averaged values and standard deviations are given in table.

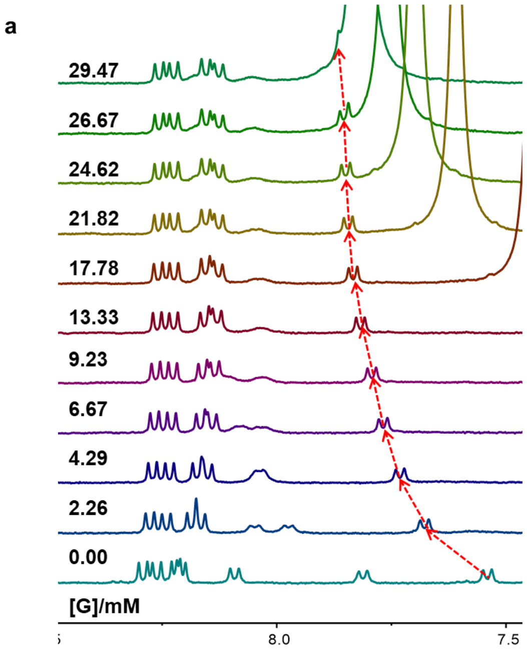

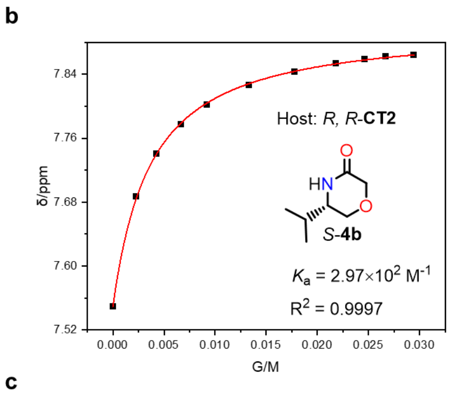

c

	K_a /M^{-1}	R^2
Titration-1	2.97×10^2	0.9997
Titration-2	4.17×10^2	0.9989
Titration-3	3.15×10^2	0.9995
Average	(3.4±0.5)×10^2	

Supplementary Figure 70. NMR titrations and association constants. a Partial ^{1}H NMR spectra (500 MHz, Toluene-d$_8$, 298 K) of *R,R*-**CT2** (0.5 mM) titrated by *S*-**4b**. From bottom to top, the concentration range of 1 is 0 ~ 29.47 mM. **b** Nonlinear curve fitting for the complexation between *R,R*-**CT2** and *S*-**4b** based on the NMR data, and **c** the averaged values and standard deviations are given in table.

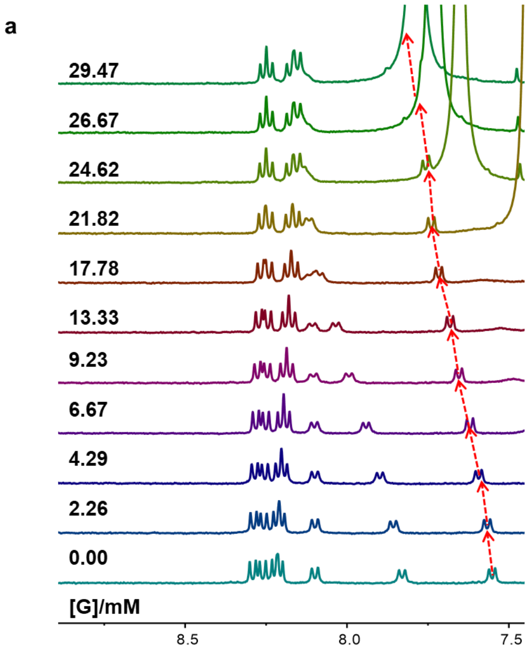

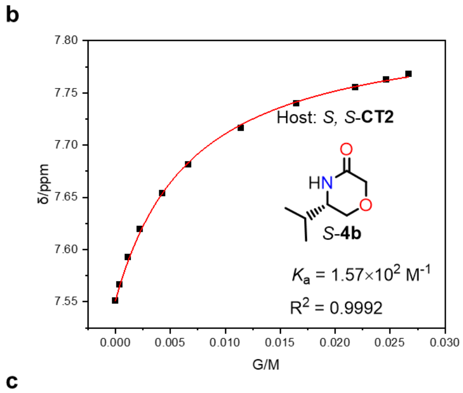

c

	K_a /M^{-1}	R^2
Titration-1	1.57×10^2	0.9992
Titration-2	1.72×10^2	0.9994
Titration-3	2.08×10^2	0.9998
Average	(1.8±0.2)×10^2	

Supplementary Figure 71. NMR titrations and association constants. a Partial ^{1}H NMR spectra (500 MHz, Toluene-d$_8$, 298 K) of *S,S*-**CT2** (0.5 mM) titrated by *S*-**4b**. From bottom to top, the concentration range of 1 is 0 ~ 29.47 mM. **b** Nonlinear curve fitting for the complexation between *S,S*-**CT2** and *S*-**4b** based on the NMR data, and **c** the averaged values and standard deviations are given in table.

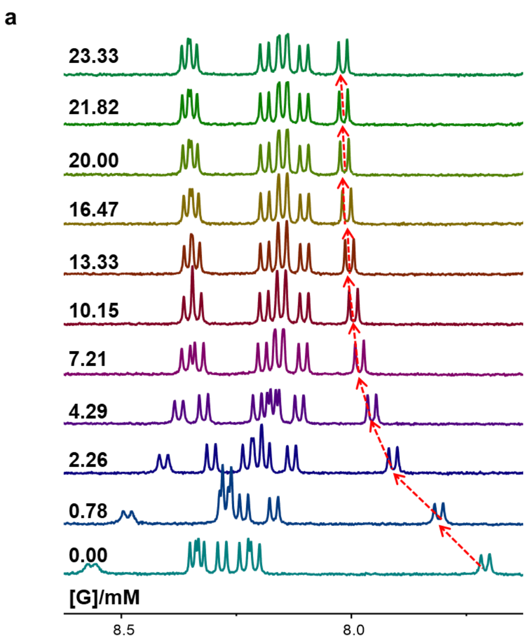

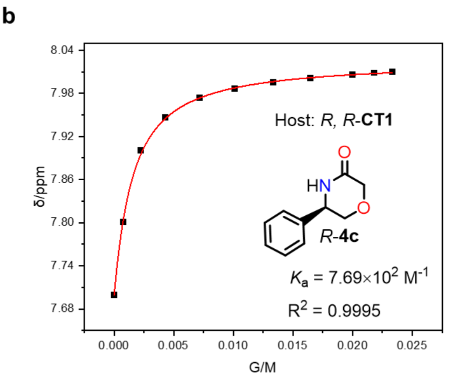

c

	K_a /M^{-1}	R^2
Titration-1	7.69×10^2	0.9995
Titration-2	8.63×10^2	0.9998
Titration-3	7.63×10^2	0.9993
Average	(8.0±0.4)×10^2	

Supplementary Figure 72. NMR titrations and association constants. a Partial ^{1}H NMR spectra (500 MHz, Toluene-d$_8$, 298 K) of *R,R*-**CT1** (0.5 mM) titrated by *R*-**4c**. From bottom to top, the concentration range of 1 is 0 ~ 23.33 mM. **b** Nonlinear curve fitting for the complexation between *R,R*-**CT1** and *R*-**4c** based on the NMR data, and **c** the averaged values and standard deviations are given in table.

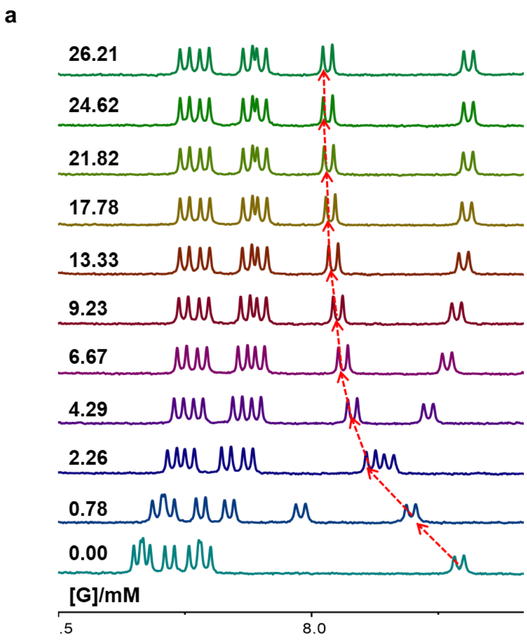

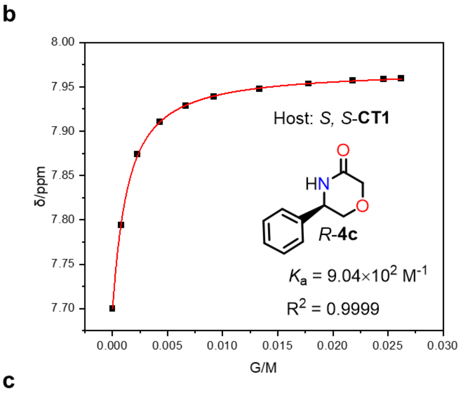

c

	K_a /M^{-1}	R^2
Titration-1	9.04×10^2	0.9999
Titration-2	8.89×10^2	0.9999
Titration-3	8.97×10^2	0.9999
Average	(9.0±0.1)×10^2	

Supplementary Figure 73. NMR titrations and association constants. a Partial ^{1}H NMR spectra (500 MHz, Toluene-d$_8$, 298 K) of *S,S*-**CT1** (0.5 mM) titrated by *R*-**4c**. From bottom to top, the concentration range of 1 is 0 ~ 26.21 mM. **b** Nonlinear curve fitting for the complexation between *S,S*-**CT1** and *R*-**4c** based on the NMR data, and **c** the averaged values and standard deviations are given in table.

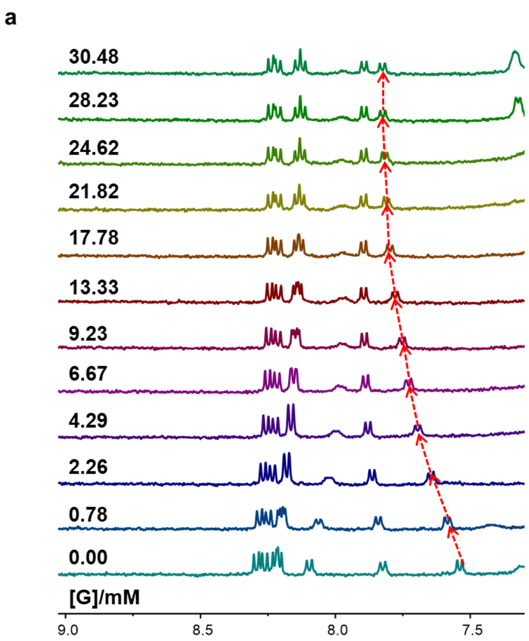

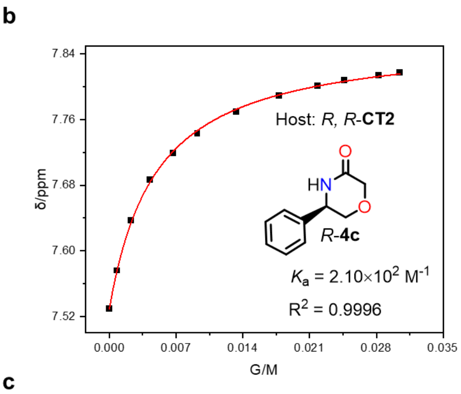

c

	K_a /M^{-1}	R^2
Titration-1	2.10×10^2	0.9996
Titration-2	2.09×10^2	0.9995
Titration-3	2.19×10^2	0.9995
Average	(2.1±0.1)×10^2	

Supplementary Figure 74. NMR titrations and association constants. a Partial ^{1}H NMR spectra (500 MHz, Toluene-d$_8$, 298 K) of *R,R*-**CT2** (0.5 mM) titrated by *R*-**4c**. From bottom to top, the concentration range of 1 is 0 ~ 30.48 mM. **b** Nonlinear curve fitting for the complexation between *R,R*-**CT2** and *R*-**4c** based on the NMR data, and **c** the averaged values and standard deviations are given in table.

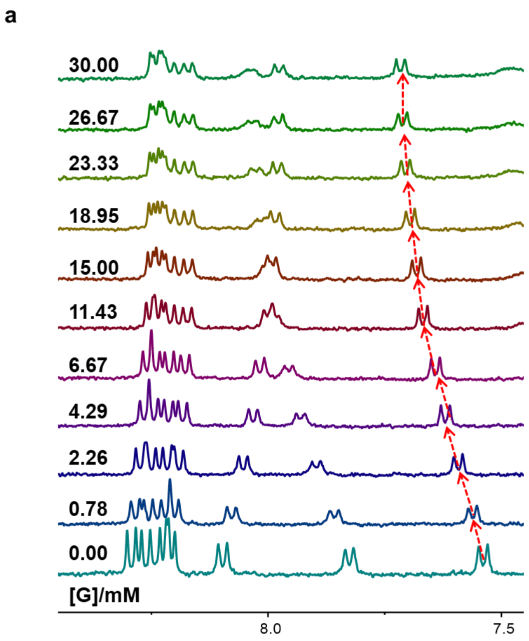

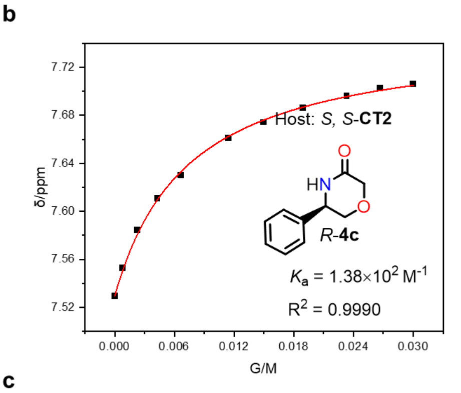

c

	K_a /M^{-1}	R^2
Titration-1	1.38×10^2	0.9990
Titration-2	1.58×10^2	0.9987
Titration-3	1.67×10^2	0.9998
Average	(1.5±0.1)×10^2	

Supplementary Figure 75. NMR titrations and association constants. a Partial ^{1}H NMR spectra (500 MHz, Toluene-d$_8$, 298 K) of *S,S*-**CT2** (0.5 mM) titrated by *R*-**4c**. From bottom to top, the concentration range of 1 is 0 ~ 30.00 mM. **b** Nonlinear curve fitting for the complexation between *S,S*-**CT2** and *R*-**4c** based on the NMR data, and **c** the averaged values and standard deviations are given in table.

a

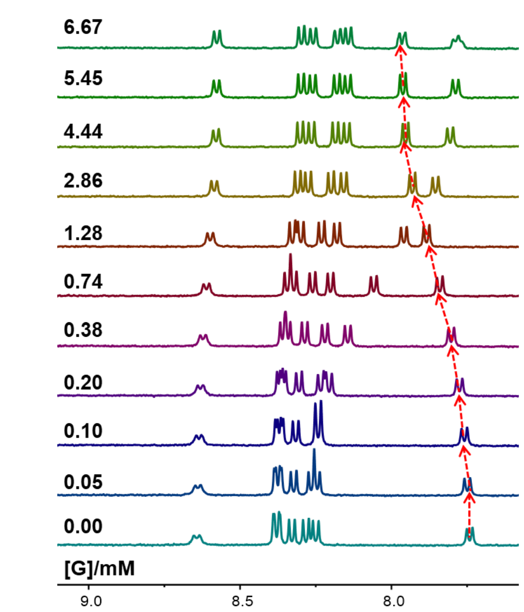

b

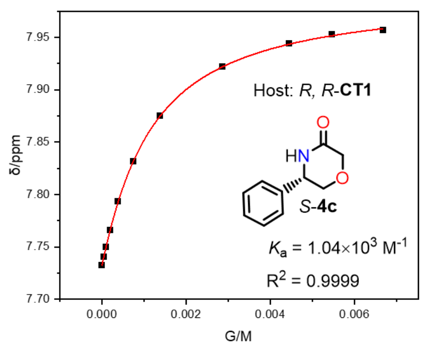

c

	K_a /M^{-1}	R^2
Titration-1	1.04×10^3	0.9999
Titration-2	1.09×10^3	0.9998
Titration-3	1.11×10^3	0.9998
Average	(1.1±0.1)×10^3	

Supplementary Figure 76. NMR titrations and association constants. a Partial ^{1}H NMR spectra (500 MHz, Toluene-d$_8$, 298 K) of *R,R*-**CT1** (0.5 mM) titrated by *S*-**4c**. From bottom to top, the concentration range of 1 is 0 ~ 6.67 mM. **b** Nonlinear curve fitting for the complexation between *R,R*-**CT1** and *S*-**4c** based on the NMR data, and **c** the averaged values and standard deviations are given in table.

a

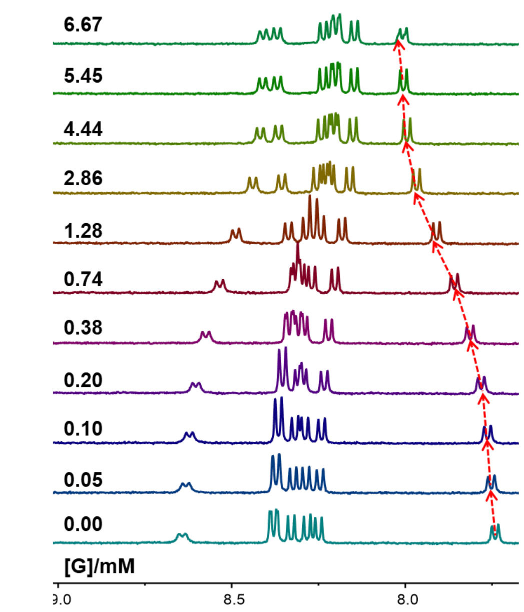

b

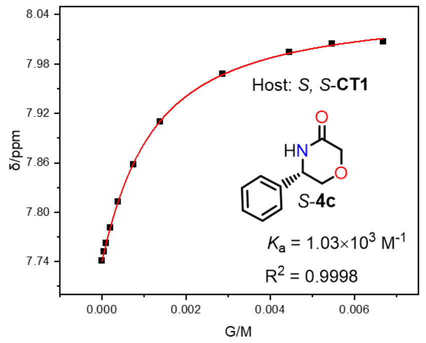

c

	K_a /M^{-1}	R^2
Titration-1	1.03×10^3	0.9998
Titration-2	1.19×10^3	0.9997
Titration-3	1.16×10^3	0.9998
Average	(1.1±0.2)×10^3	

Supplementary Figure 77. NMR titrations and association constants. a Partial ^{1}H NMR spectra (500 MHz, Toluene-d$_8$, 298 K) of *S,S*-**CT1** (0.5 mM) titrated by *S*-**4c**. From bottom to top, the concentration range of 1 is 0 ~ 6.67 mM. **b** Nonlinear curve fitting for the complexation between *S,S*-**CT1** and *S*-**4c** based on the NMR data, and **c** the averaged values and standard deviations are given in table.

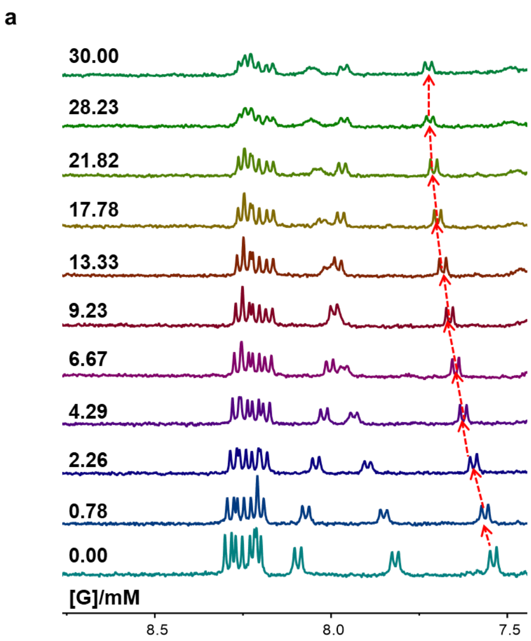

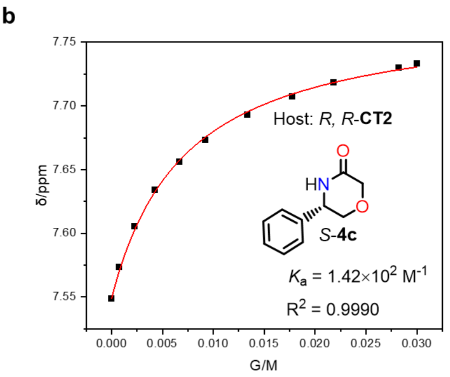

c

	K_a /M^{-1}	R^2
Titration-1	1.42×10^2	0.9990
Titration-2	1.83×10^2	0.9997
Titration-3	1.71×10^2	0.9982
Average	$(1.7\pm0.2)\times10^2$	

Supplementary Figure 78. NMR titrations and association constants. a Partial ^{1}H NMR spectra (500 MHz, Toluene-d$_8$, 298 K) of *R,R*-**CT2** (0.5 mM) titrated by *S*-**4c**. From bottom to top, the concentration range of 1 is 0 ~ 30.00 mM. **b** Nonlinear curve fitting for the complexation between *R,R*-**CT2** and *S*-**4c** based on the NMR data, and **c** the averaged values and standard deviations are given in table.

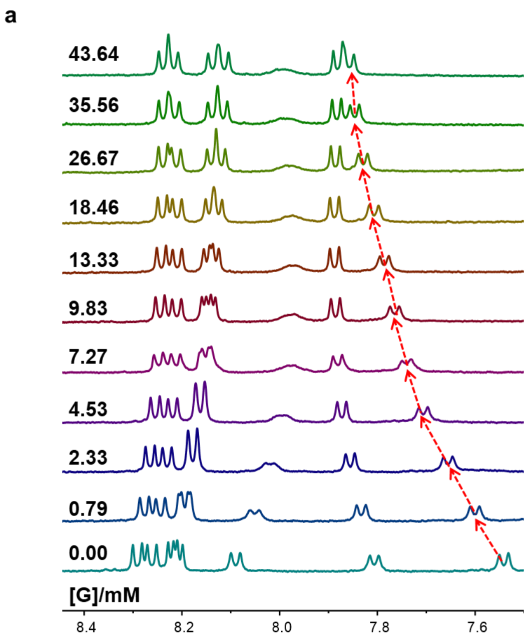

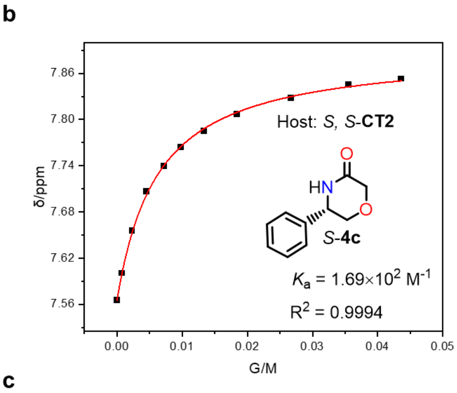

c

	K_a /M^{-1}	R^2
Titration-1	1.69×10^2	0.9994
Titration-2	1.80×10^2	0.9982
Titration-3	1.72×10^2	0.9983
Average	$(1.7\pm0.1)\times10^2$	

Supplementary Figure 79. NMR titrations and association constants. a Partial ^{1}H NMR spectra (500 MHz, Toluene-d$_8$, 298 K) of *S,S*-**CT2** (0.5 mM) titrated by *S*-**4c**. From bottom to top, the concentration range of 1 is 0 ~ 43.64 mM. **b** Nonlinear curve fitting for the complexation between *S,S*-**CT2** and *S*-**4c** based on the NMR data, and **c** the averaged values and standard deviations are given in table.

a

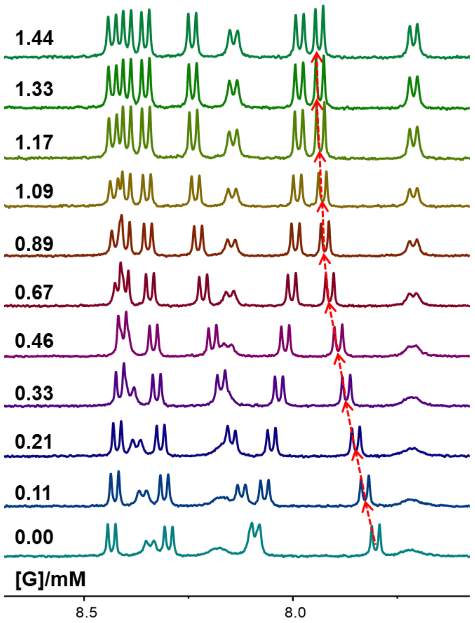

b

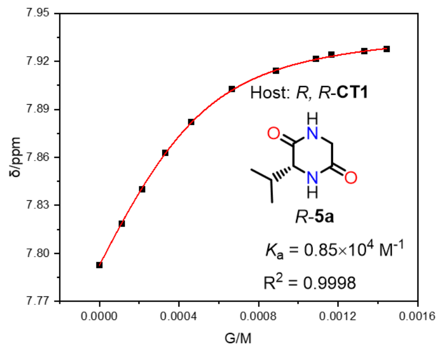

c

	K_a /M^{-1}	R^2
Titration-1	0.85×10^4	0.9998
Titration-2	1.09×10^4	0.9998
Titration-3	1.02×10^4	0.9999
Average	(1.0±0.1)×10^4	

Supplementary Figure 80. NMR titrations and association constants. a Partial ^{1}H NMR spectra (500 MHz, $CDCl_3$, 298 K) of *R,R*-**CT1** (0.5 mM) titrated by *R*-**5a**. From bottom to top, the concentration range of 1 is 0 ~ 1.44 mM. **b** Nonlinear curve fitting for the complexation between *R,R*-**CT1** and *R*-**5a** based on the NMR data, and **c** the averaged values and standard deviations are given in table.

a

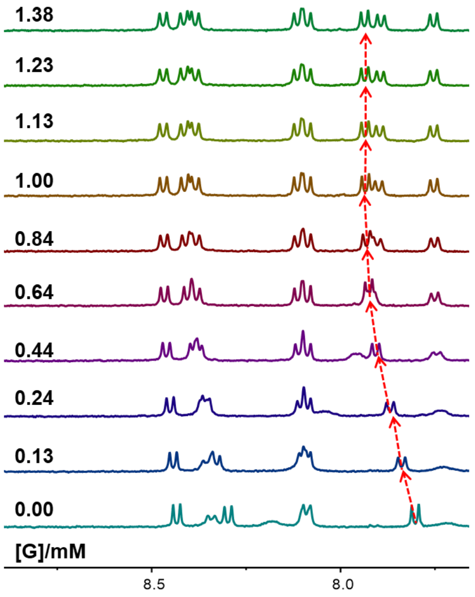

b

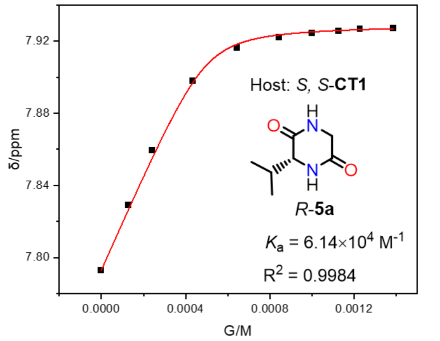

c

	K_a /M^{-1}	R^2
Titration-1	6.14×10^4	0.9984
Titration-2	5.43×10^4	0.9992
Titration-3	5.13×10^4	0.9992
Average	(5.6±0.4)×10^4	

Supplementary Figure 81. NMR titrations and association constants. a Partial ^{1}H NMR spectra (500 MHz, $CDCl_3$, 298 K) of *S,S*-**CT1** (0.5 mM) titrated by *R*-**5a**. From bottom to top, the concentration range of 1 is 0 ~ 1.38 mM. **b** Nonlinear curve fitting for the complexation between *S,S*-**CT1** and *R*-**5a** based on the NMR data, and **c** the averaged values and standard deviations are given in table.

a

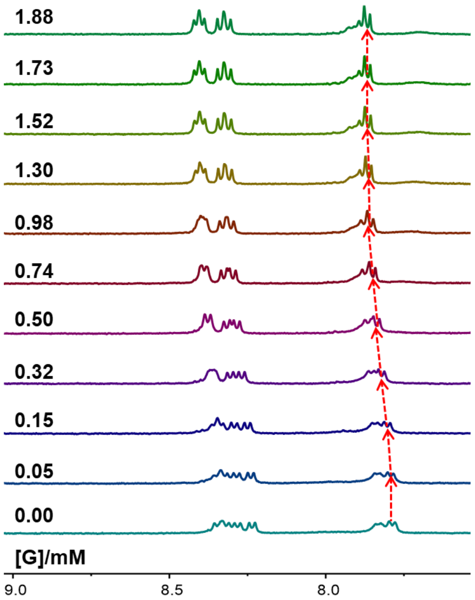

b

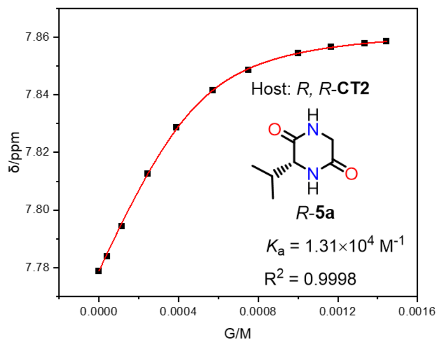

c

	K_a /M^{-1}	R^2
Titration-1	1.31×10^4	0.9998
Titration-2	1.74×10^4	0.9996
Titration-3	1.50×10^4	0.9996
Average	(1.5±0.2)×10^4	

Supplementary Figure 82. NMR titrations and association constants. a Partial ^{1}H NMR spectra (500 MHz, $CDCl_3$, 298 K) of *R,R*-**CT2** (0.5 mM) titrated by *R*-**5a**. From bottom to top, the concentration range of 1 is 0 ~ 1.88 mM. **b** Nonlinear curve fitting for the complexation between *R,R*-**CT2** and *R*-**5a** based on the NMR data, and **c** the averaged values and standard deviations are given in table.

a

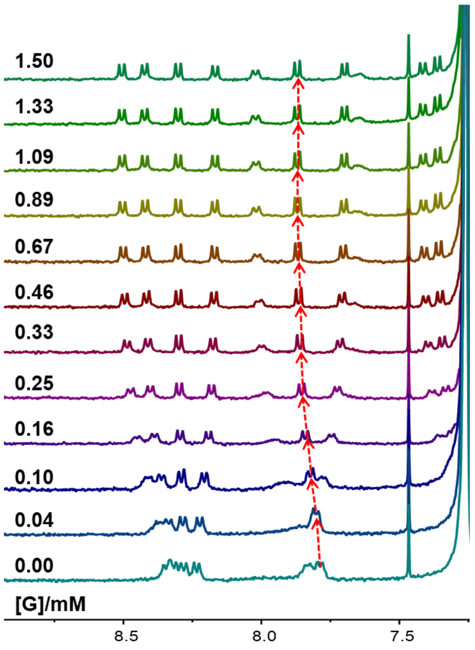

b

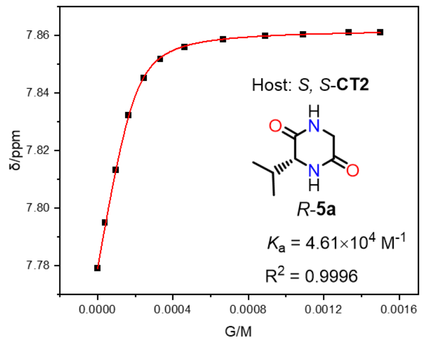

c

	K_a /M^{-1}	R^2
Titration-1	4.61×10^4	0.9996
Titration-2	4.35×10^4	0.9992
Titration-3	4.82×10^4	0.9999
Average	(4.6±0.2)×10^4	

Supplementary Figure 83. NMR titrations and association constants. a Partial ^{1}H NMR spectra (500 MHz, $CDCl_3$, 298 K) of *S,S*-**CT2** (0.5 mM) titrated by *R*-**5a**. From bottom to top, the concentration range of 1 is 0 ~ 1.50 mM. **b** Nonlinear curve fitting for the complexation between *S,S*-**CT2** and *R*-**5a** based on the NMR data, and **c** the averaged values and standard deviations are given in table.

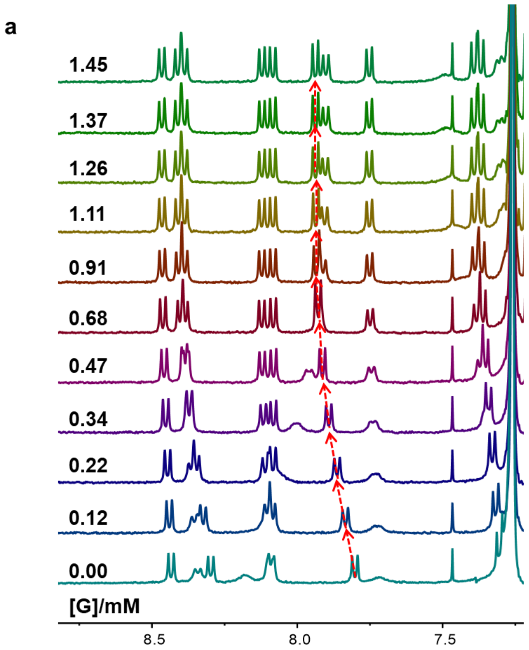

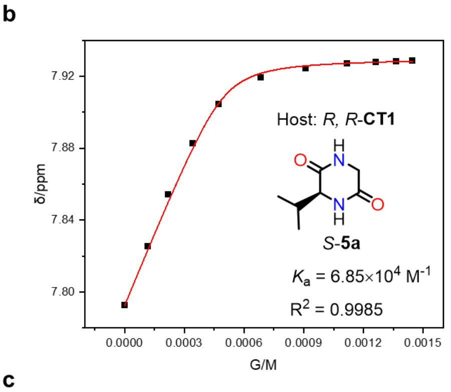

c

	K_a /M^{-1}	R^2
Titration-1	6.85×10^4	0.9985
Titration-2	6.34×10^4	0.9977
Titration-3	6.97×10^4	0.9974
Average	$(6.7\pm0.3)\times10^4$	

Supplementary Figure 84. NMR titrations and association constants. a Partial ^{1}H NMR spectra (500 MHz, $CDCl_3$, 298 K) of *R*,*R*-**CT1** (0.5 mM) titrated by *S*-**5a**. From bottom to top, the concentration range of 1 is 0 ~ 1.45 mM. **b** Nonlinear curve fitting for the complexation between *R*,*R*-**CT1** and *S*-**5a** based on the NMR data, and **c** the averaged values and standard deviations are given in table.

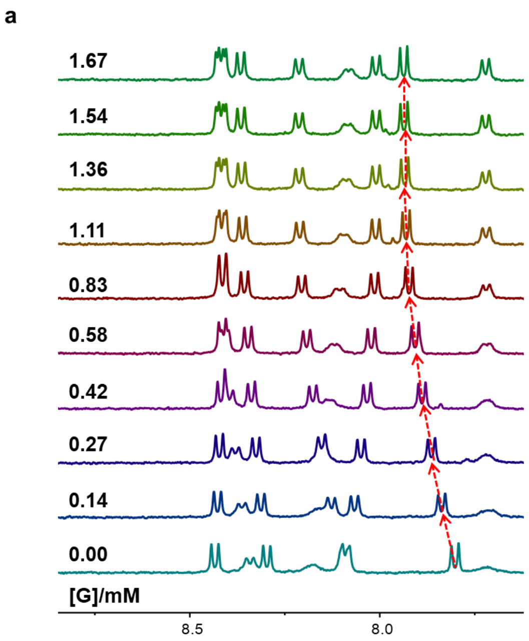

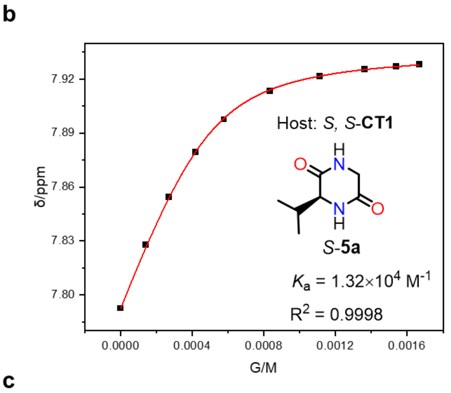

c

	K_a /M^{-1}	R^2
Titration-1	1.32×10^4	0.9998
Titration-2	1.45×10^4	0.9997
Titration-3	1.02×10^4	0.9999
Average	$(1.3\pm0.2)\times10^4$	

Supplementary Figure 85. NMR titrations and association constants. a Partial ^{1}H NMR spectra (500 MHz, $CDCl_3$, 298 K) of *S*,*S*-**CT1** (0.5 mM) titrated by *S*-**5a**. From bottom to top, the concentration range of 1 is 0 ~ 1.45 mM. **b** Nonlinear curve fitting for the complexation between *S*,*S*-**CT1** and *S*-**5a** based on the NMR data, and **c** the averaged values and standard deviations are given in table.

a

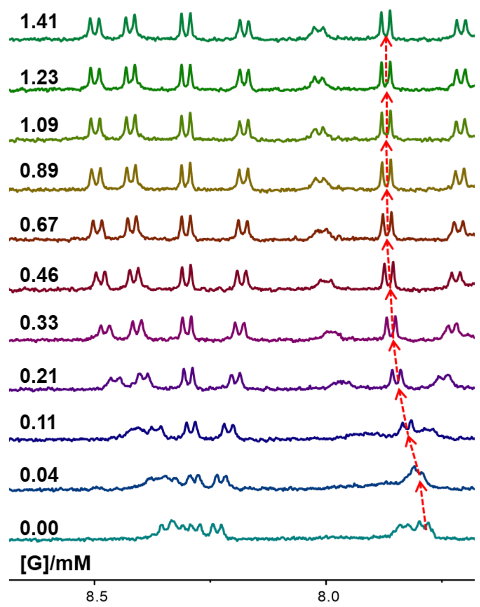

b

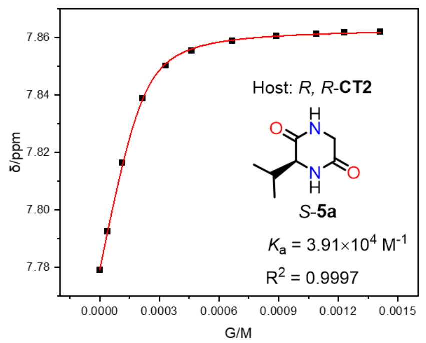

c

	K_a /M^{-1}	R^2
Titration-1	3.91×10^4	0.9997
Titration-2	4.33×10^4	0.9995
Titration-3	4.46×10^4	0.9980
Average	(4.2±0.2)×10^4	

Supplementary Figure 86. NMR titrations and association constants. a Partial ^{1}H NMR spectra (500 MHz, $CDCl_3$, 298 K) of *R,R*-**CT2** (0.5 mM) titrated by *S*-**5a**. From bottom to top, the concentration range of 1 is 0 ~ 40.00 mM. **b** Nonlinear curve fitting for the complexation between *R,R*-**CT2** and *S*-**5a** based on the NMR data, and **c** the averaged values and standard deviations are given in table.

a

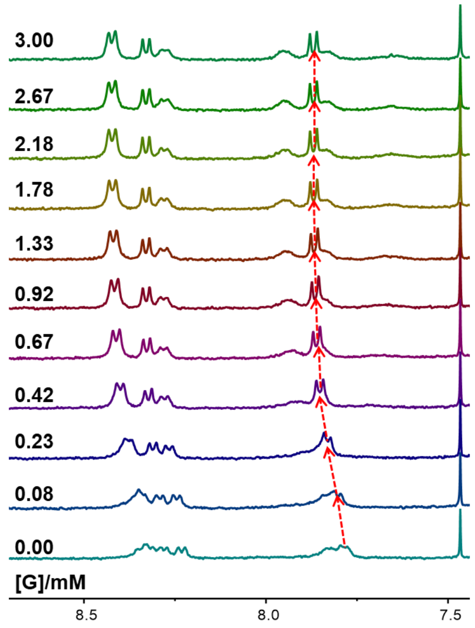

b

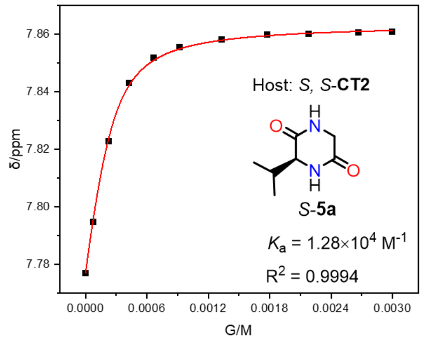

c

	K_a /M^{-1}	R^2
Titration-1	1.28×10^4	0.9994
Titration-2	1.14×10^4	0.9997
Titration-3	1.17×10^4	0.9997
Average	(1.2±0.1)×10^4	

Supplementary Figure 87. NMR titrations and association constants. a Partial ^{1}H NMR spectra (500 MHz, $CDCl_3$, 298 K) of *S,S*-**CT2** (0.5 mM) titrated by *S*-**5a**. From bottom to top, the concentration range of 1 is 0 ~ 3.00 mM. **b** Nonlinear curve fitting for the complexation between *S,S*-**CT2** and *S*-**5a** based on the NMR data, and **c** the averaged values and standard deviations are given in table.

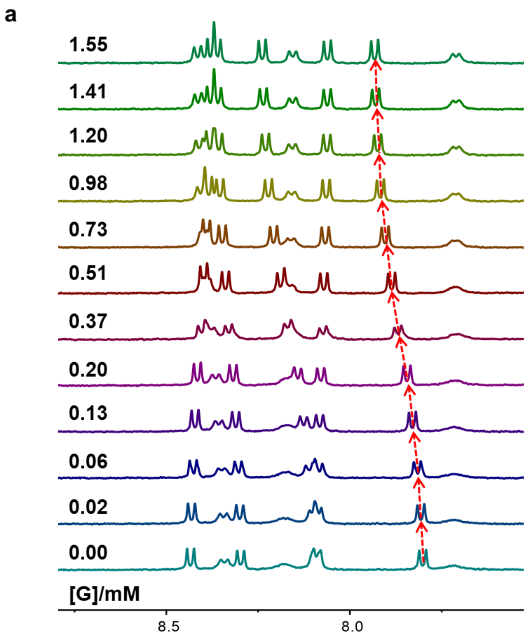

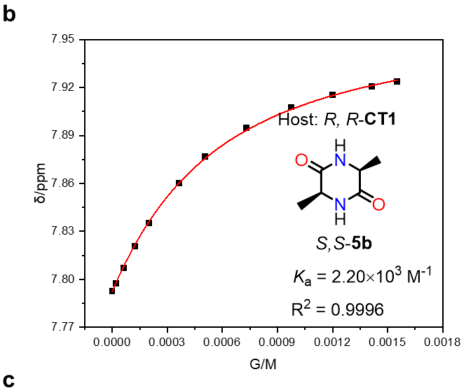

c

	K_a /M^{-1}	R^2
Titration-1	2.20×10^3	0.9996
Titration-2	2.61×10^3	0.9999
Titration-3	2.18×10^3	0.9994
Average	$(2.3\pm0.2)\times10^3$	

Supplementary Figure 88. NMR titrations and association constants. a Partial ^{1}H NMR spectra (500 MHz, $CDCl_3$, 298 K) of *R,R*-**CT1** (0.5 mM) titrated by *R,R*-**5b**. From bottom to top, the concentration range of 1 is 0 ~ 1.55 mM. **b** Nonlinear curve fitting for the complexation between *R,R*-**CT1** and *R,R*-**5b** based on the NMR data, and **c** the averaged values and standard deviations are given in table.

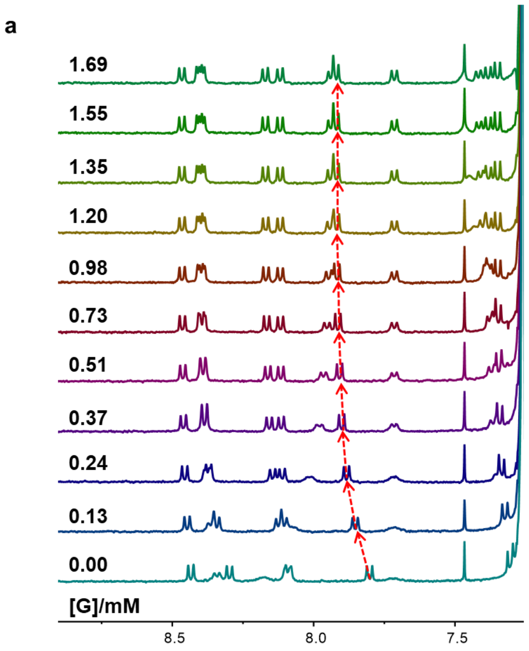

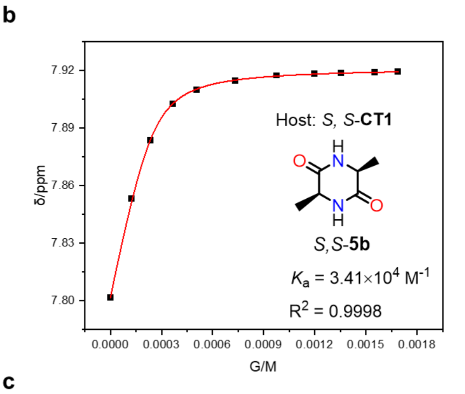

c

	K_a /M^{-1}	R^2
Titration-1	3.41×10^4	0.9998
Titration-2	4.05×10^4	0.9991
Titration-3	4.27×10^4	0.9986
Average	$(3.9\pm0.4)\times10^4$	

Supplementary Figure 89. NMR titrations and association constants. a Partial ^{1}H NMR spectra (500 MHz, $CDCl_3$, 298 K) of *S,S*-**CT1** (0.5 mM) titrated by *R,R*-**5b**. From bottom to top, the concentration range of 1 is 0 ~ 1.69 mM. **b** Nonlinear curve fitting for the complexation between *S,S*-**CT1** and *R,R*-**5b** based on the NMR data, and **c** the averaged values and standard deviations are given in table.

a

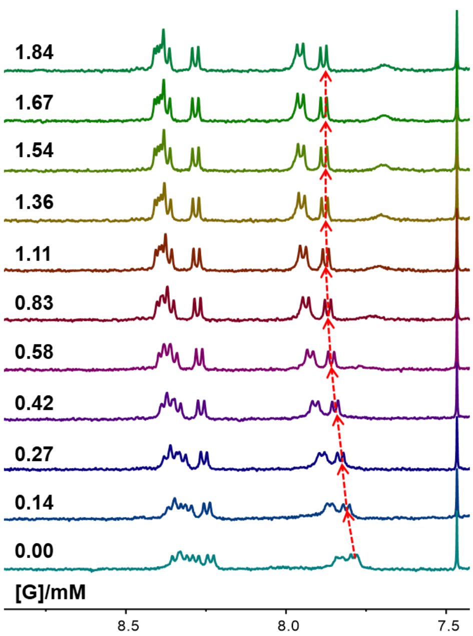

b

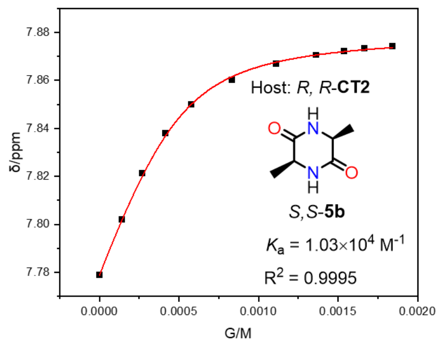

c

	K_a /M^{-1}	R^2
Titration-1	1.03×10^4	0.9995
Titration-2	0.96×10^4	0.9998
Titration-3	0.93×10^4	0.9995
Average	(1.0±0.1)×10^4	

Supplementary Figure 90. NMR titrations and association constants. a Partial ^{1}H NMR spectra (500 MHz, $CDCl_3$, 298 K) of *R,R*-**CT2** (0.5 mM) titrated by *R,R*-**5b**. From bottom to top, the concentration range of 1 is 0 ~ 1.84 mM. **b** Nonlinear curve fitting for the complexation between *R,R*-**CT2** and *R,R*-**5b** based on the NMR data, and **c** the averaged values and standard deviations are given in table.

a

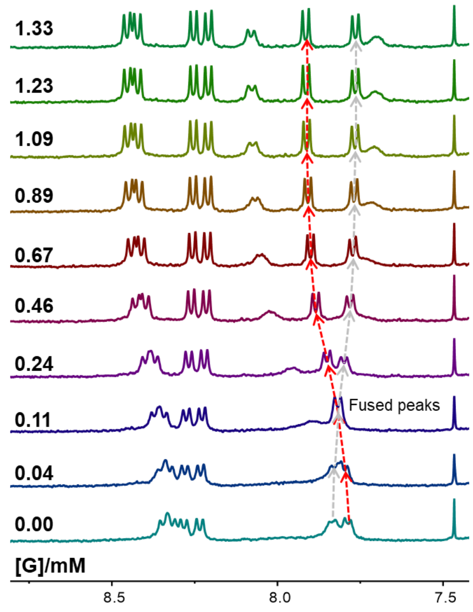

b

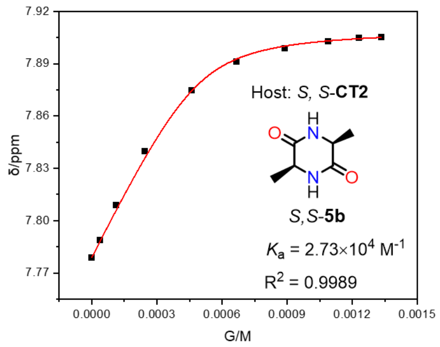

c

	K_a /M^{-1}	R^2
Titration-1	2.73×10^4	0.9989
Titration-2	3.01×10^4	0.9992
Titration-3	3.30×10^4	0.9981
Average	(3.0±0.2)×10^4	

Supplementary Figure 91. NMR titrations and association constants. a Partial ^{1}H NMR spectra (500 MHz, $CDCl_3$, 298 K) of *S,S*-**CT2** (0.5 mM) titrated by *S,S*-**5b**. From bottom to top, the concentration range of 1 is 0 ~ 1.33 mM. **b** Nonlinear curve fitting for the complexation between *S,S*-**CT2** and *S,S*-**5b** based on the NMR data, and **c** the averaged values and standard deviations are given in table.

a

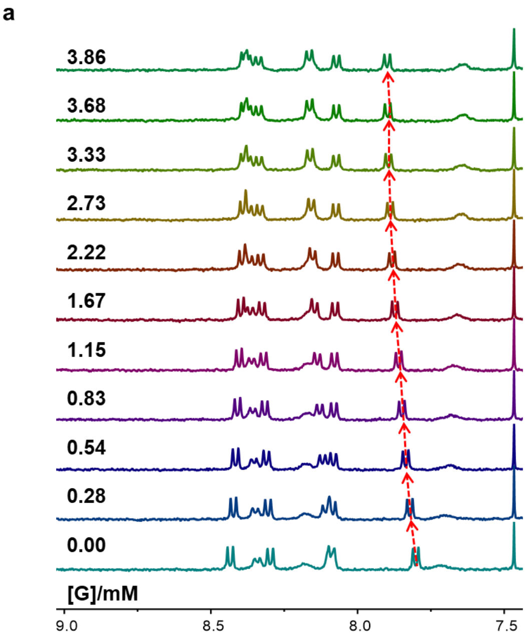

b

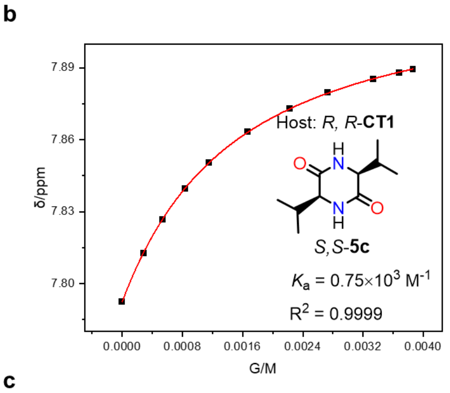

c

	K_a /M^{-1}	R^2
Titration-1	0.75×10^3	0.9999
Titration-2	0.83×10^3	0.9992
Titration-3	1.03×10^3	0.9999
Average	(8.7±1.1)×10^2	

Supplementary Figure 92. NMR titrations and association constants. a Partial ^{1}H NMR spectra (500 MHz, $CDCl_3$, 298 K) of *R,R*-**CT1** (0.5 mM) titrated by *S,S*-**5c**. From bottom to top, the concentration range of 1 is 0 ~ 3.86 mM. **b** Nonlinear curve fitting for the complexation between *R,R*-**CT1** and *R,R*-**5c** based on the NMR data, and **c** the averaged values and standard deviations are given in table.

a

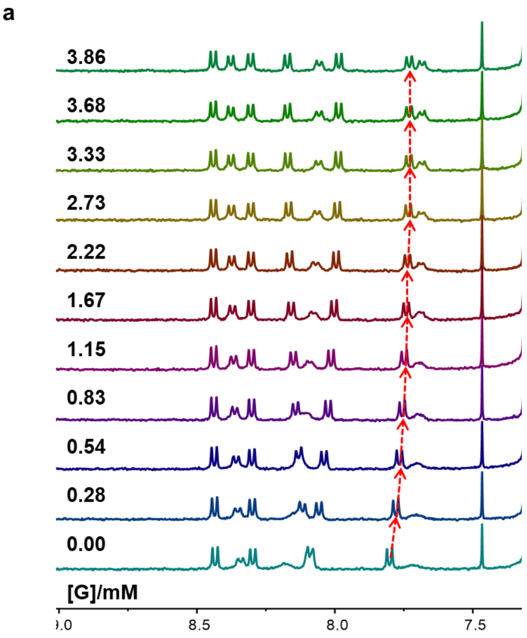

b

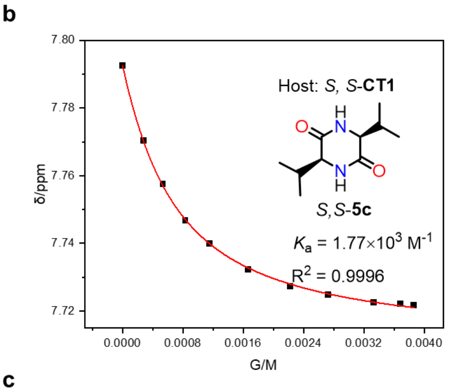

c

	K_a /M^{-1}	R^2
Titration-1	1.77×10^3	0.9996
Titration-2	1.87×10^3	0.9999
Titration-3	1.88×10^3	0.9999
Average	(1.8±0.1)×10^3	

Supplementary Figure 93. NMR titrations and association constants. a Partial ^{1}H NMR spectra (500 MHz, $CDCl_3$, 298 K) of *S,S*-**CT1** (0.5 mM) titrated by *S,S*-**5c**. From bottom to top, the concentration range of 1 is 0 ~ 3.86 mM. **b** Nonlinear curve fitting for the complexation between *S,S*-**CT1** and *S,S*-**5c** based on the NMR data, and **c** the averaged values and standard deviations are given in table.

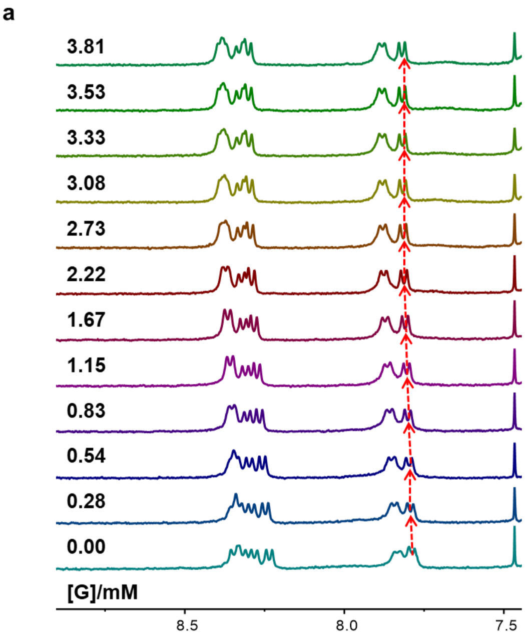

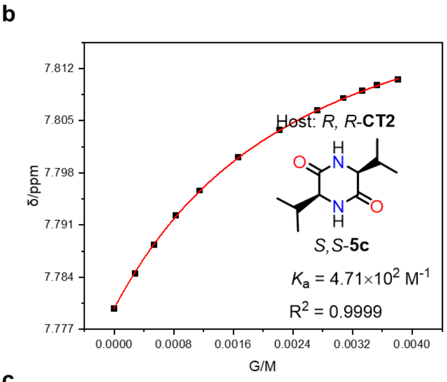

c

	K_a /M^{-1}	R^2
Titration-1	4.71×10^2	0.9999
Titration-2	5.41×10^2	0.9992
Titration-3	4.93×10^2	0.9994
Average	(5.0±0.3)×10^2	

Supplementary Figure 94. NMR titrations and association constants. a Partial ^{1}H NMR spectra (500 MHz, $CDCl_3$, 298 K) of *R,R*-**CT2** (0.5 mM) titrated by *S,S*-**5c**. From bottom to top, the concentration range of 1 is 0 ~ 3.81 mM. **b** Nonlinear curve fitting for the complexation between *R,R*-**CT2** and *S,S*-**5c** based on the NMR data, and **c** the averaged values and standard deviations are given in table.

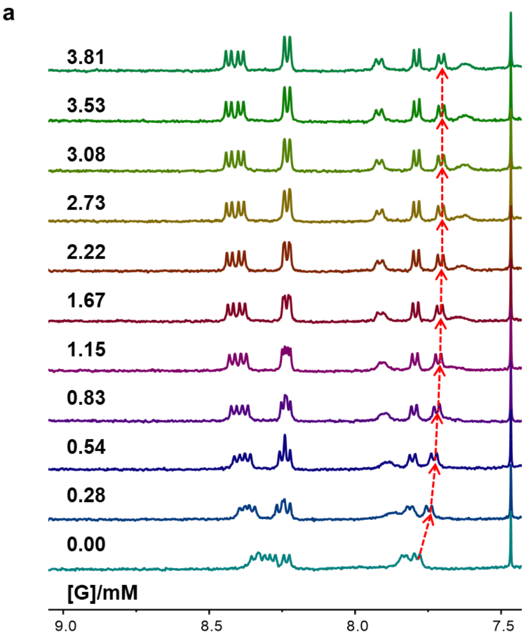

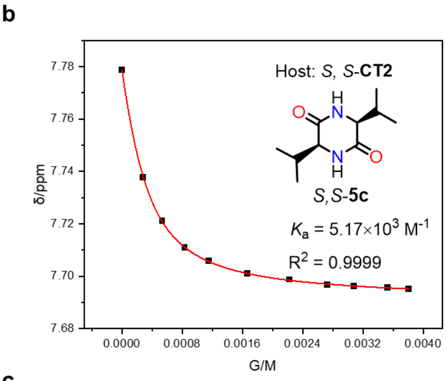

c

	K_a /M^{-1}	R^2
Titration-1	5.17×10^3	0.9999
Titration-2	5.95×10^3	0.9990
Titration-3	5.03×10^3	0.9994
Average	(5.4±0.4)×10^3	

Supplementary Figure 95. NMR titrations and association constants. a Partial ^{1}H NMR spectra (500 MHz, $CDCl_3$, 298 K) of *S,S*-**CT2** (0.5 mM) titrated by *S,S*-**5c**. From bottom to top, the concentration range of 1 is 0 ~ 3.81 mM. **b** Nonlinear curve fitting for the complexation between *S,S*-**CT2** and *S,S*-**5c** based on the NMR data, and **c** the averaged values and standard deviations are given in table.

a

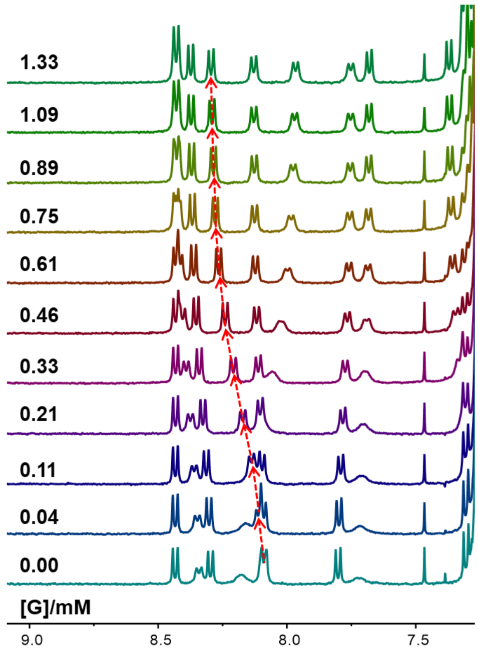

b

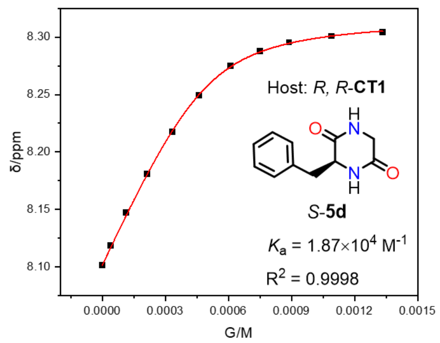

c

	K_a /M^{-1}	R^2
Titration-1	1.87×10^4	0.9998
Titration-2	1.42×10^4	0.9999
Titration-3	1.47×10^4	0.9999
Average	$(1.6\pm0.2)\times10^4$	

Supplementary Figure 96. NMR titrations and association constants. a Partial ^{1}H NMR spectra (500 MHz, $CDCl_3$, 298 K) of *R,R*-**CT1** (0.5 mM) titrated by *S*-**5d**. From bottom to top, the concentration range of 1 is 0 ~ 1.33 mM. **b** Nonlinear curve fitting for the complexation between *R,R*-**CT1** and *S*-**5d** based on the NMR data, and **c** the averaged values and standard deviations are given in table.

a

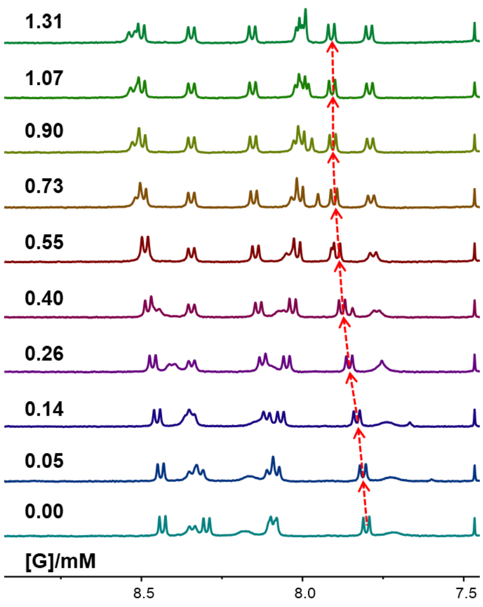

b

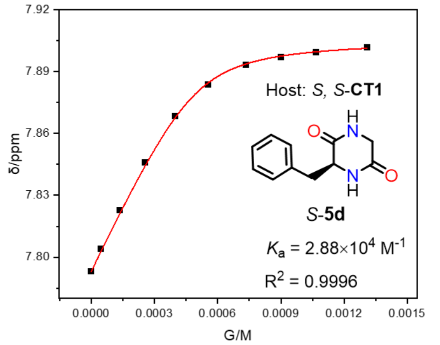

c

	K_a /M^{-1}	R^2
Titration-1	2.88×10^4	0.9996
Titration-2	3.15×10^4	0.9985
Titration-3	2.80×10^4	0.9990
Average	$(2.9\pm0.1)\times10^4$	

Supplementary Figure 97. NMR titrations and association constants. a Partial ^{1}H NMR spectra (500 MHz, $CDCl_3$, 298 K) of *S,S*-**CT1** (0.5 mM) titrated by *S*-**5d**. From bottom to top, the concentration range of 1 is 0 ~ 1.31 mM. **b** Nonlinear curve fitting for the complexation between *S,S*-**CT1** and *S*-**5d** based on the NMR data, and **c** the averaged values and standard deviations are given in table.

a

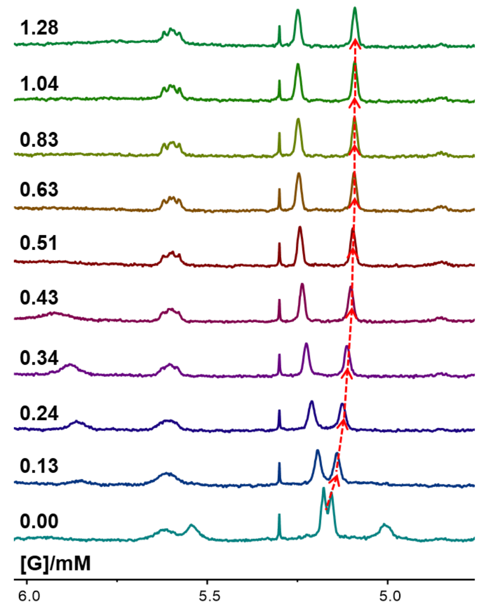

b

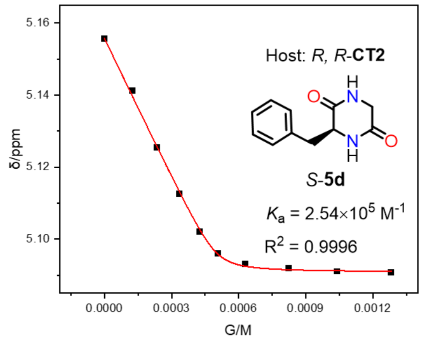

c

	K_a /M^{-1}	R^2
Titration-1	2.54×10^5	0.9993
Titration-2	2.47×10^5	0.9991
Titration-3	1.96×10^5	0.9995
Average	$(2.3\pm0.3)\times10^5$	

Supplementary Figure 98. NMR titrations and association constants. a Partial ^{1}H NMR spectra (500 MHz, $CDCl_3$, 298 K) of *R,R*-**CT2** (0.5 mM) titrated by *S*-**5d**. From bottom to top, the concentration range of 1 is 0 ~ 1.28 mM. **b** Nonlinear curve fitting for the complexation between *R,R*-**CT2** and *S*-**5d** based on the NMR data, and **c** the averaged values and standard deviations are given in table.

a

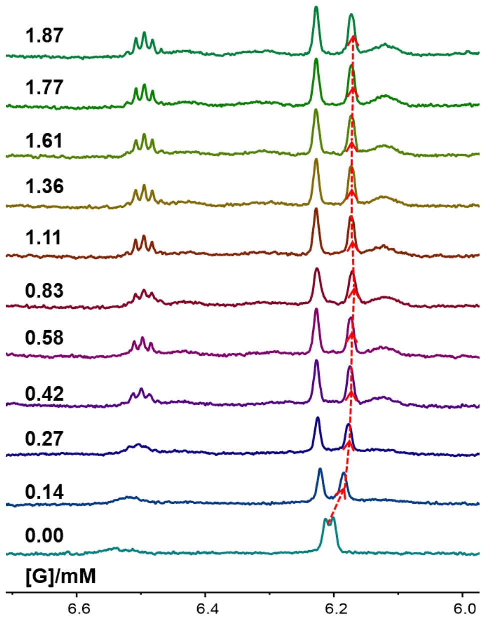

b

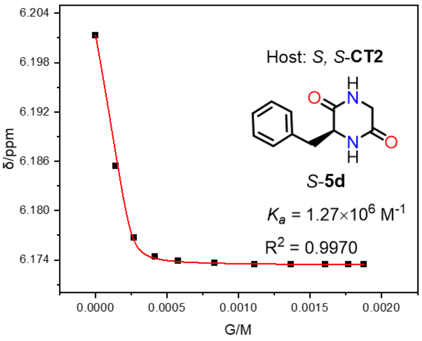

c

	K_a /M^{-1}	R^2
Titration-1	1.27×10^6	0.9970
Titration-2	1.54×10^6	0.9951
Titration-3	1.25×10^6	0.9961
Average	$(1.4\pm0.1)\times10^6$	

Supplementary Figure 99. NMR titrations and association constants. a Partial ^{1}H NMR spectra (500 MHz, $CDCl_3$, 298 K) of *S,S*-**CT2** (0.5 mM) titrated by *S*-**5d**. From bottom to top, the concentration range of 1 is 0 ~ 1.87 mM. **b** Nonlinear curve fitting for the complexation between *S,S*-**CT2** and *S*-**5d** based on the NMR data, and **c** the averaged values and standard deviations are given in table.

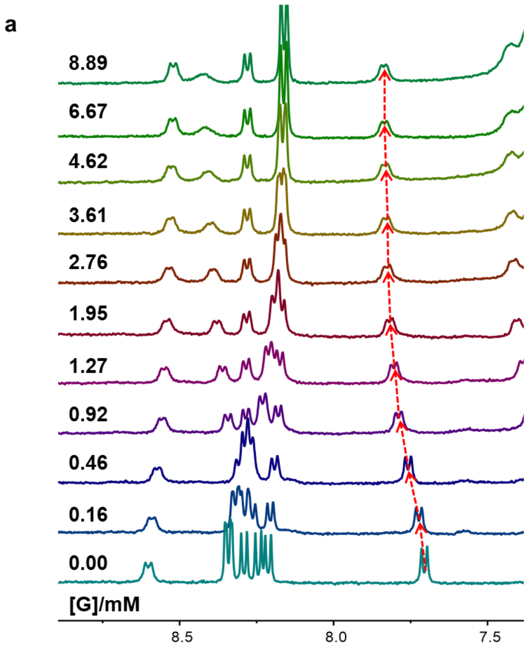

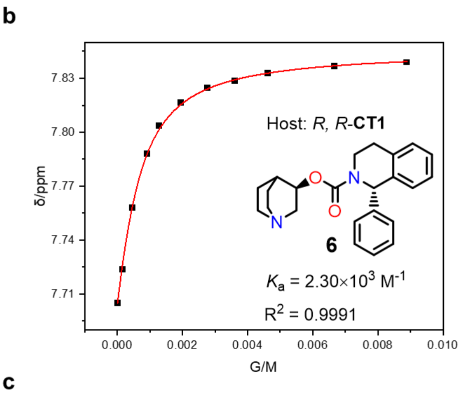

c

	K_a /M^{-1}	R^2
Titration-1	2.30×10^3	0.9991
Titration-2	2.29×10^3	0.9982
Titration-3	1.89×10^3	0.9987
Average	(2.2±0.2)×10^3	

Supplementary Figure 100. NMR titrations and association constants. a Partial ^{1}H NMR spectra (500 MHz, Toluene-d$_8$, 298 K) of *R,R*-**CT1** (0.5 mM) titrated by **6**. From bottom to top, the concentration range of 1 is 0 ~ 8.89 mM. **b** Nonlinear curve fitting for the complexation between *R,R*-**CT1** and **6** based on the NMR data, and **c** the averaged values and standard deviations are given in table.

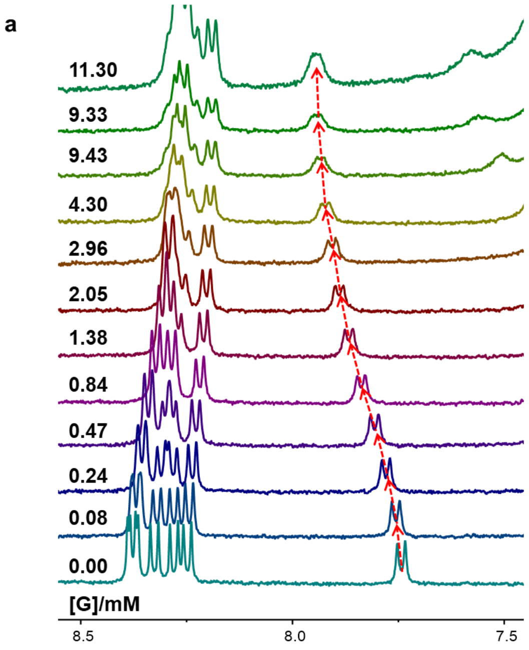

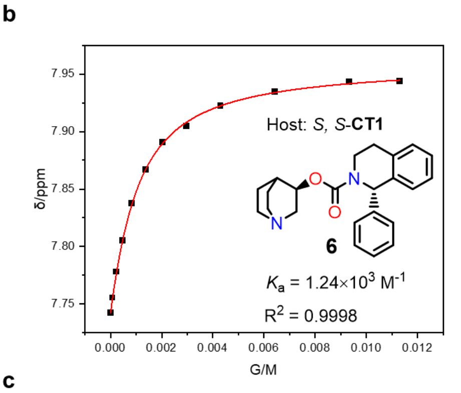

c

	K_a /M^{-1}	R^2
Titration-1	1.24×10^3	0.9998
Titration-2	1.01×10^3	0.9997
Titration-3	0.91×10^3	0.9994
Average	(1.1±0.1)×10^3	

Supplementary Figure 101. NMR titrations and association constants. a Partial ^{1}H NMR spectra (500 MHz, Toluene-d$_8$, 298 K) of *S,S*-**CT1** (0.5 mM) titrated by **6**. From bottom to top, the concentration range of 1 is 0 ~ 11.30 mM. **b** Nonlinear curve fitting for the complexation between *S,S*-**CT1** and **6** based on the NMR data, and **c** the averaged values and standard deviations are given in table.

a

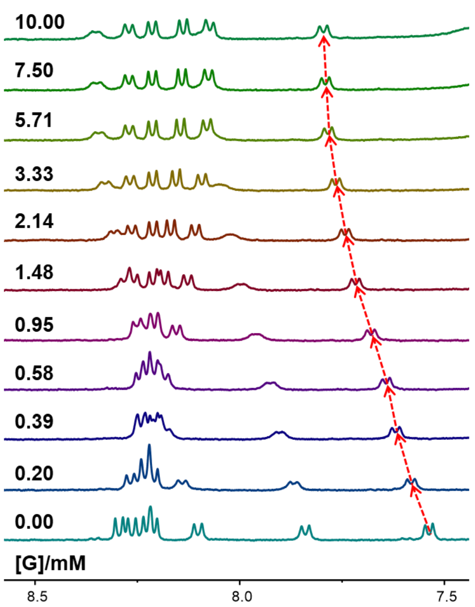

b

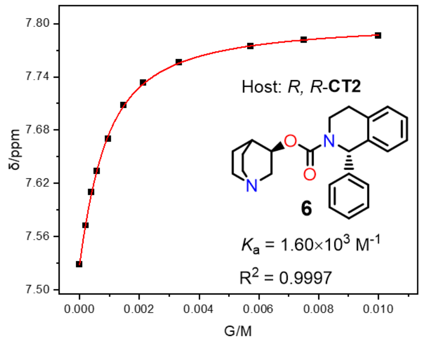

c

	K_a /M^{-1}	R^2
Titration-1	1.60×10³	0.9997
Titration-2	1.91×10³	0.9975
Titration-3	1.69×10³	0.9990
Average	(1.7±0.1)×10³	

Supplementary Figure 102. NMR titrations and association constants. a Partial ^{1}H NMR spectra (500 MHz, Toluene-d$_8$, 298 K) of *R,R*-**CT2** (0.5 mM) titrated by **6**. From bottom to top, the concentration range of 1 is 0 ~ 10.00 mM. **b** Nonlinear curve fitting for the complexation between *R,R*-**CT2** and **6** based on the NMR data, and **c** the averaged values and standard deviations are given in table.

a

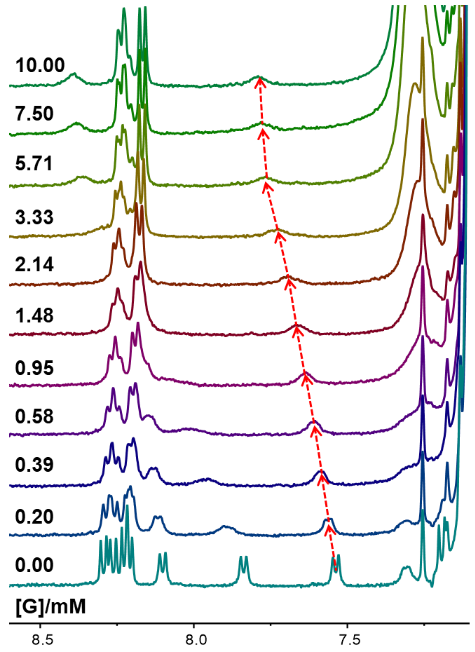

b

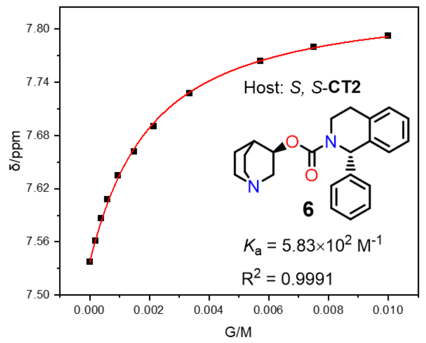

c

	K_a /M^{-1}	R^2
Titration-1	5.83×10²	0.9991
Titration-2	4.50×10²	0.9980
Titration-3	5.88×10²	0.9987
Average	(5.4±0.6)×10²	

Supplementary Figure 103. NMR titrations and association constants. a Partial ^{1}H NMR spectra (500 MHz, Toluene-d$_8$, 298 K) of *S,S*-**CT2** (0.5 mM) titrated by **6**. From bottom to top, the concentration range of 1 is 0 ~ 10.00 mM. **b** Nonlinear curve fitting for the complexation between *S,S*-**CT2** and **6** based on the NMR data, and **c** the averaged values and standard deviations are given in table.

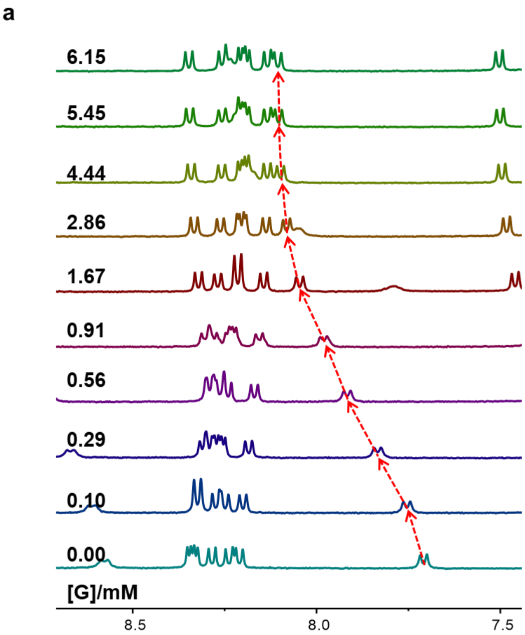

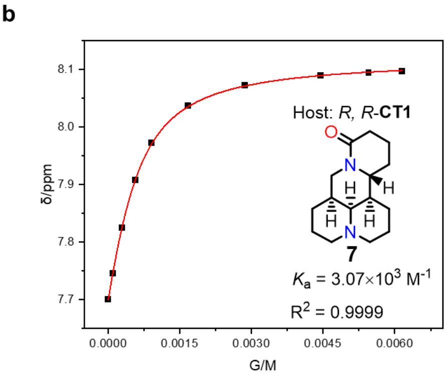

c

	K_a /M^{-1}	R^2
Titration-1	3.07×10^3	0.9999
Titration-2	3.03×10^3	0.9996
Titration-3	3.12×10^3	0.9999
Average	(3.1±0.1)×10^3	

Supplementary Figure 104. NMR titrations and association constants. a Partial ^{1}H NMR spectra (500 MHz, Toluene-d$_8$, 298 K) of *R,R*-**CT1** (0.5 mM) titrated by **7**. From bottom to top, the concentration range of 1 is 0 ~ 6.15 mM. **b** Nonlinear curve fitting for the complexation between *R,R*-**CT1** and **7** based on the NMR data, and **c** the averaged values and standard deviations are given in table.

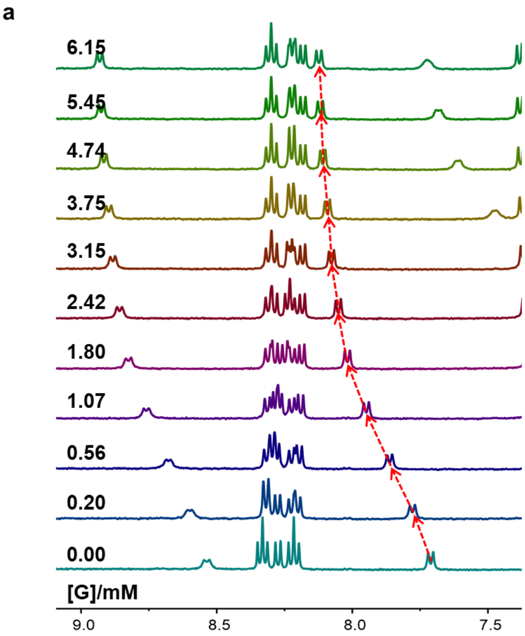

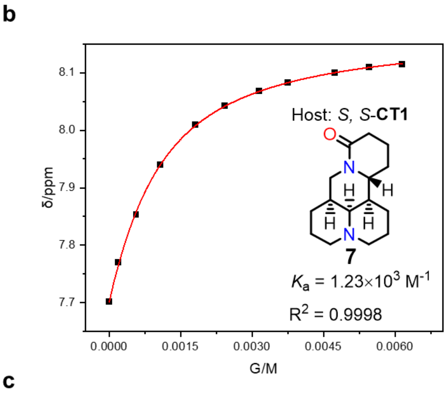

c

	K_a /M^{-1}	R^2
Titration-1	1.23×10^3	0.9998
Titration-2	1.13×10^3	0.9999
Titration-3	1.10×10^3	0.9999
Average	(1.2±0.1)×10^3	

Supplementary Figure 105. NMR titrations and association constants. a Partial ^{1}H NMR spectra (500 MHz, Toluene-d$_8$, 298 K) of *S,S*-**CT1** (0.5 mM) titrated by **7**. From bottom to top, the concentration range of 1 is 0 ~ 6.15 mM. **b** Nonlinear curve fitting for the complexation between *S,S*-**CT1** and **7** based on the NMR data, and **c** the averaged values and standard deviations are given in table.

a

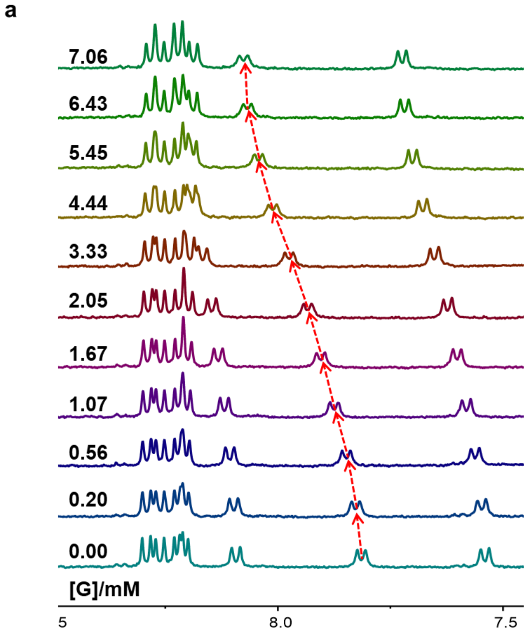

b

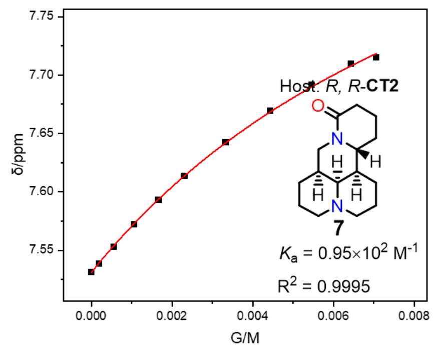

c

	K_a /M^{-1}	R^2
Titration-1	0.95×10^2	0.9995
Titration-2	0.88×10^2	0.9997
Titration-3	0.90×10^2	0.9995
Average	91±3	

Supplementary Figure 106. NMR titrations and association constants. a Partial ^{1}H NMR spectra (500 MHz, Toluene-d$_8$, 298 K) of *R*,*R*-**CT2** (0.5 mM) titrated by **7**. From bottom to top, the concentration range of 1 is 0 ~ 7.06 mM. **b** Nonlinear curve fitting for the complexation between *R,R*-**CT2** and **7** based on the NMR data, and **c** the averaged values and standard deviations are given in table.

a

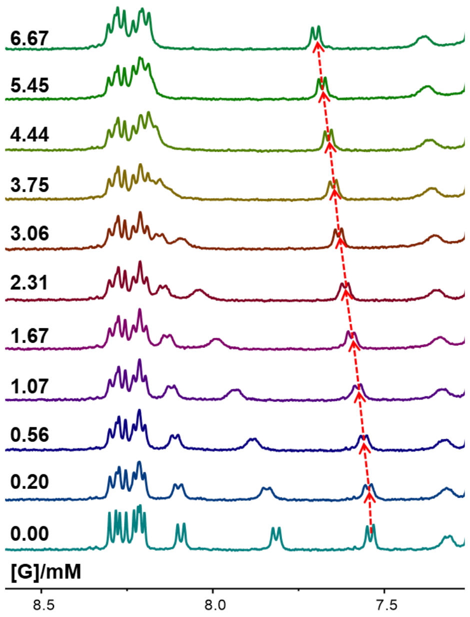

b

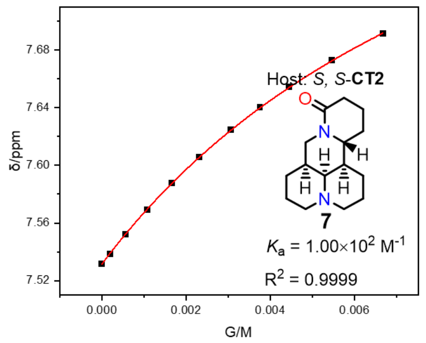

c

	K_a /M^{-1}	R^2
Titration-1	1.00×10^2	0.9999
Titration-2	0.91×10^2	0.9998
Titration-3	1.05×10^2	0.9995
Average	99±6	

Supplementary Figure 107. NMR titrations and association constants. a Partial ^{1}H NMR spectra (500 MHz, Toluene-d$_8$, 298 K) of *S*,*S*-**CT2** (0.5 mM) titrated by **7**. From bottom to top, the concentration range of 1 is 0 ~ 6.67 mM. **b** Nonlinear curve fitting for the complexation between *S,S*-**CT2** and **7** based on the NMR data, and **c** the averaged values and standard deviations are given in table.

2.12. X-Ray Single Crystallography

- Suitable single crystals of *R,R*-**CT2** and *S,S*-**CT2** were successfully obtained by slow evaporation of their saturated solutions in CH_2Cl_2 and acetone mixed solution.
- Suitable single crystals of *R*-**5a**@*S,S*-**CT2** and *S*-**5a**@*S,S*-**CT2** were successfully obtained by slow evaporation of their saturated solutions of CH_2Cl_2 and toluene.
- Suitable single crystals of *R,R*-**5b**@*S,S*-**CT2** were successfully obtained by slow evaporation of its saturated solution of CH_2Cl_2 and toluene.
- Suitable single crystals of *S,S*-**5c**@*S,S*-**CT2** were obtained by slow evaporation of their saturated solutions in CH_2Cl_2 and toluene mixed solution. Single crystal of 2**Toluene**@*S,S*-**CT2** was obtained by slow evaporation of *R,R*-**5c** and *S,S*-**CT2** saturated solutions in CH_2Cl_2 and toluene mixed solution.

Single crystal X-ray data were collected on a Bruker D8 VENTURE with Ga Kα radiation (λ = 1.34138 Å) at 100 K. The structures were solved by intrinsic phasing methods (SHELXT) and refined by full-matrix least-squares F^2 using SHELXL[9] in the OLEX2 program package.[10] All non-hydrogen atoms were refined with anisotropic thermal parameters and the hydrogen atoms were fixed at calculated positions and refined by a riding mode.

Supplementary Table *1*: Crystal data and structure refinement for *R,R*-**CT2•**2Acetone, *S,S*-**CT2•**2Acetone, *R*-**5a@***S,S*-**CT2**, *S*-**5a@***S,S*-**CT2**, *S,S*-**5c@***S,S*-**CT2**, 2**Toluene@***R,R*-**CT2**, *S,S*-**5b@***R,R*-**CT2** complex

entry	*R,R*-**CT2•**2Acetone	*S,S*-**CT2•**2Acetone	*R*-**5a@***S,S*-**CT2**	*S*-**5a@***S,S*-**CT2**	*S,S*-**5c@***S,S*-**CT2**	2**Toluene@***R,R*-**CT2**	*S,S*-**5b@***R,R*-**CT2**
Empirical formula	$C_{76}H_{88}N_4O_{10}S_2$	$C_{76}H_{88}N_4O_{10}S_2$	$C_{77}H_{88}N_6O_{10}S_2$	$C_{77}H_{88}N_6O_{10}S_2$	$C_{102.57}H_{119.79}N_6O_{10}S_2$	$C_{84}H_{92}N_4O_8S_2$	$C_{78.4}H_{87.8}N_{5.4}O_{9.4}S_2$
Formula weight	1281.62	1281.62	1321.65	1321.65	1660.76	1349.73	1320.26
Temperature/K	100.00	100.0	100.00	100.00	100.00	100.00	100.00
Crystal system	monoclinic	monoclinic	monoclinic	monoclinic	monoclinic	monoclinic	monoclinic
Space group	*P* 2_1	*P* 2_1 m	*P* 2_1	*P* 2_1	*C* 2	*P* 2_1	*P* 2_1 m
a/Å	11.0748(8)	11.0817(6)	10.9618(3)	10.9865(7)	23.962(3)	11.2491(4)	10.9848(5)
b/Å	17.0269(12)	17.0128(10)	17.1073(5)	17.1302(10)	19.289(2)	17.1295(6)	17.0970(7)
c/Å	18.2174(13)	18.2036(11)	18.2899(5)	18.2901(11)	21.308(3)	18.1480(6)	18.3349(7)
α/°	90	90	90	90	90	90	90
β/°	95.968(3)	95.979(2)	97.5270(10)	97.639(2)	101.636(5)	96.0400(10)	96.457(2)
γ/°	90	90	90	90	90	90	90
Volume/Å^3	3416.6(4)	3413.3(3)	3400.29(17)	3411.7(4)	9646(2)	3477.6(2)	3421.6(2)
Z	2	2	2	2	4	2	2
ρ_{calc}g/cm^3	1.246	1.247	1.291	1.287	1.144	1.289	1.281
μ/mm^{-1}	0.782	0.783	0.802	0.799	0.627	0.772	0.791
F(000)	1368.0	1368.0	1408.0	1408	3557.0	1440.0	1406.0
Reflections collected	79979	102196	94883	89068	57132	126720	66692
Independent reflections	16665[R_{int} = 0.0710 R_{sigma} = 0.0609]	16792[R_{int} = 0.0598 R_{sigma} = 0.0446]	16806[R_{int} = 0.0593 R_{sigma}= 0.0471]	16297[R_{int} = 0.0479 R_{sigma} = 0.0420]	19609 [R_{int} = 0.0420 R_{sigma} = 0.0486]	20963[R_{int} = 0.0564 R_{sigma}= 0.0415]	13791[R_{int} = 0.0552 R_{sigma}=0.0491]
Data/restraints/parameters	16665/8/845	16792/8/850	16806/1966/895	16297/171/862	19609/1740/1559	20963/1/895	13791/566/965
Goodness-of-fit on F^2	1.081	1.056	1.088	0.931	1.069	1.088	1.063
Final R indexes [I>=2σ (I)]	R_1 = 0.0464, wR_2 = 0.1206	R_1 = 0.0384, wR_2 = 0.1019	R_1 = 0.0493, wR_2 = 0.1309	R_1 = 0.0470, wR_2 = 0.1283	R_1 = 0.0834, wR_2 = 0.2230	R_1 = 0.0493, wR_2 = 0.1267	R_1 = 0.0698, wR_2 = 0.1876
Final R indexes [all data]	R_1 = 0.0495, wR_2 = 0.1226	R_1 = 0.0416, wR_2 = 0.1043	R_1 = 0.0543, wR_2 = 0.1340	R_1 =0.0480, wR_2 = 0.1292	R_1 = 0.0928, wR_2 = 0.2358	R_1 = 0.0509, wR_2 = 0.1277	R_1 = 0.0775, wR_2 = 0.1935
CCDC number	2268760	2269592	2247191	2247223	2247283	2247282	2269211

R-**5a**@*S,S*-**CT2**

No A or B alerts

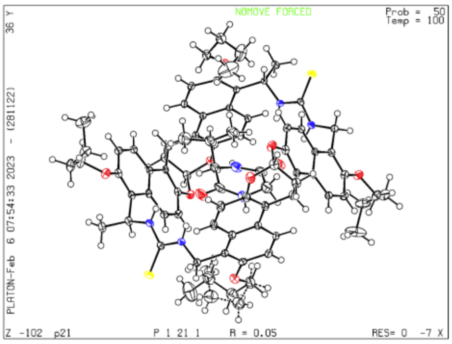

Crystal structure of *R*-**5a**@*S,S*-**CT2**.

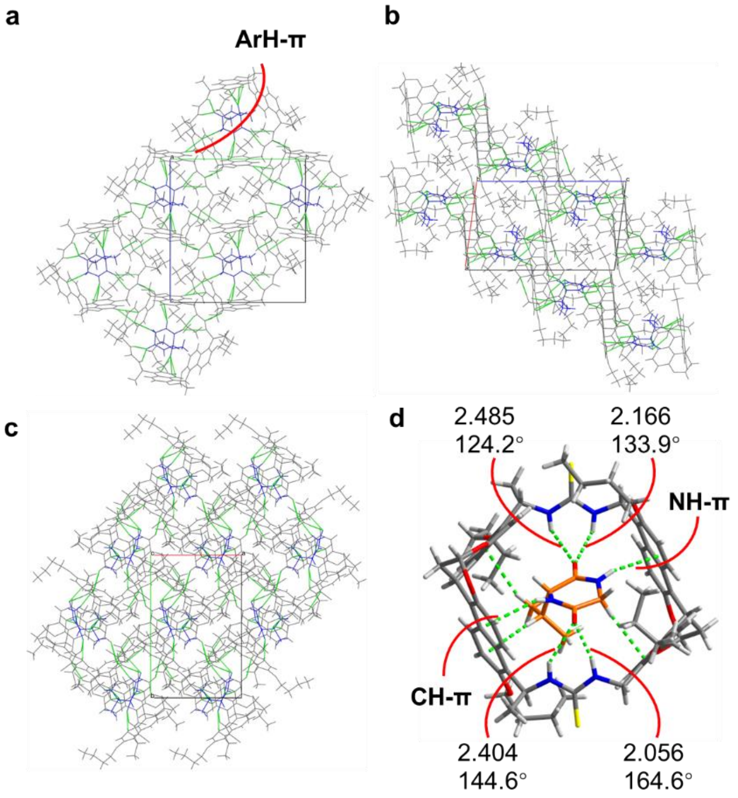

Supplementary Figure 108. Crystal data. Crystal structure of *R*-**5a**@*S,S*-**CT2**. Stacking diagrams viewed along **a** a-axis; **b** b-axis; and **c** c-axis; **d** key intermolecular interactions between host and guest. In an *S,S*-**CT2** molecule, *R*-**5a** molecule form hydrogen bondings, CH-π and NH-π with two thiourea and naphthyl groups, and there are multiple CH-π (ArH-π) interactions in the crystal to achieve crystal stability. Non-covalent interactions are shown as green dashed lines.

S-**5a**@*S,S*-**CT2**

Alert level B: PLAT220_ALERT_2_B NonSolvent Resd 1 C Ueq(max)/Ueq(min) Range 7.2 Ratio. **Response:** In general, C atoms have similar thermal parameters, but in structures where there is a tail chain, one C atom exhibits disorder, resulting in larger thermal parameters.

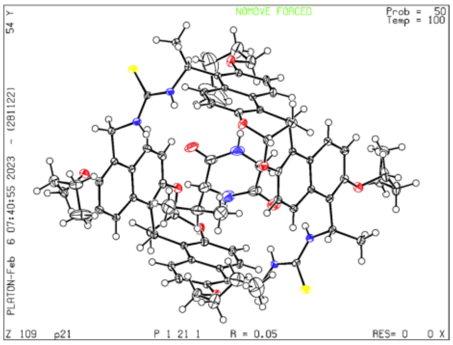

Crystal structure of *S*-**5a**@*S,S*-**CT2**.

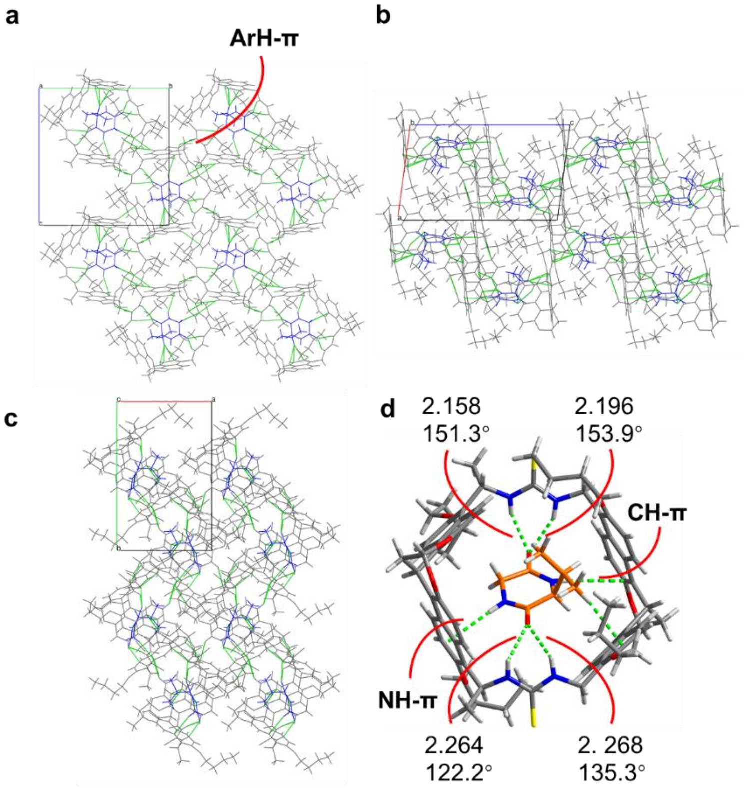

Supplementary Figure 109. Crystal data. Crystal structure of *S*-**5a**@*S,S*-**CT2**. Stacking diagrams viewed along **a** a-axis; **b** b-axis; and **c** c-axis; **d** key intermolecular interactions between host and guest. In an *S,S*-**CT2** molecule, *S*-**5a** molecule form hydrogen bondings, CH-π and NH-π with two thiourea and naphthyl groups, and there are multiple CH-π (ArH-π) interactions in the crystal to achieve crystal stability. Non-covalent interactions are shown as green dashed lines.

S,S-**5b**@*R,R*-**CT2**

No A or B alerts

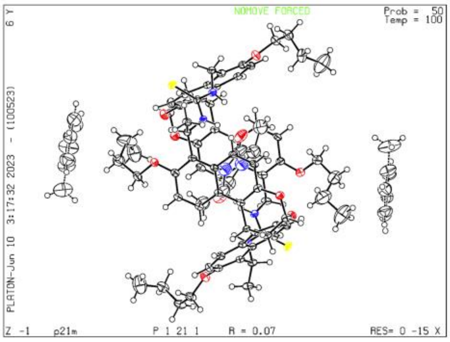

Crystal structure of *S,S*-**5b**@*R,R*-**CT2**.

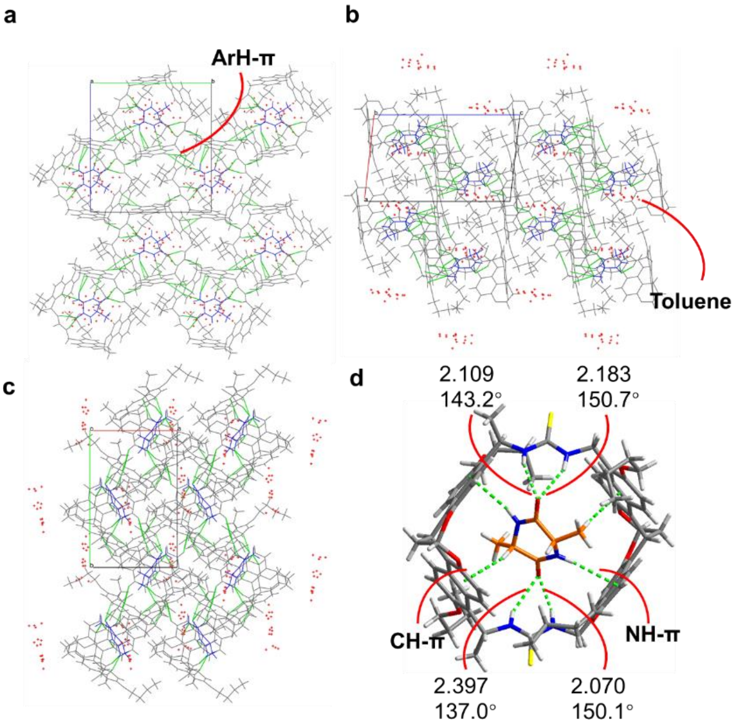

Supplementary Figure 110. Crystal data. Crystal structure of *S,S*-**5b**@*R,R*-**CT2**. Stacking diagrams viewed along **a** a-axis; **b** b-axis; and **c** c-axis; **d** key intermolecular interactions between host and guest. In an *R,R*-**CT2** molecule, *S,S*-**5b** molecule form hydrogen bondings, CH-π and NH-π with two thiourea and naphthyl groups. The red asterisks indicate the presence of toluene molecules within the interval of host-guest complexes.The toluene molecules within the interval form multiple CH-π (ArH-π) interactions with host in the crystal to achieve crystal stability. Non-covalent interactions are shown as green dashed lines.

S,S-**5c**@*S,S*-**CT2**

No A or B alerts

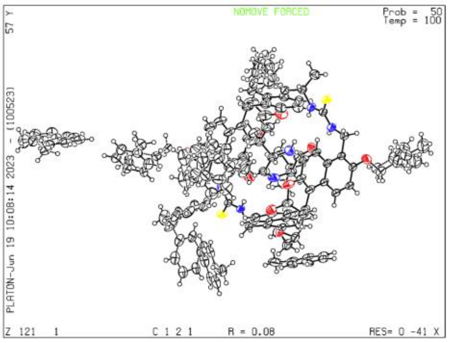

Crystal structure of *S,S*-**5c**@*S,S*-**CT2**.

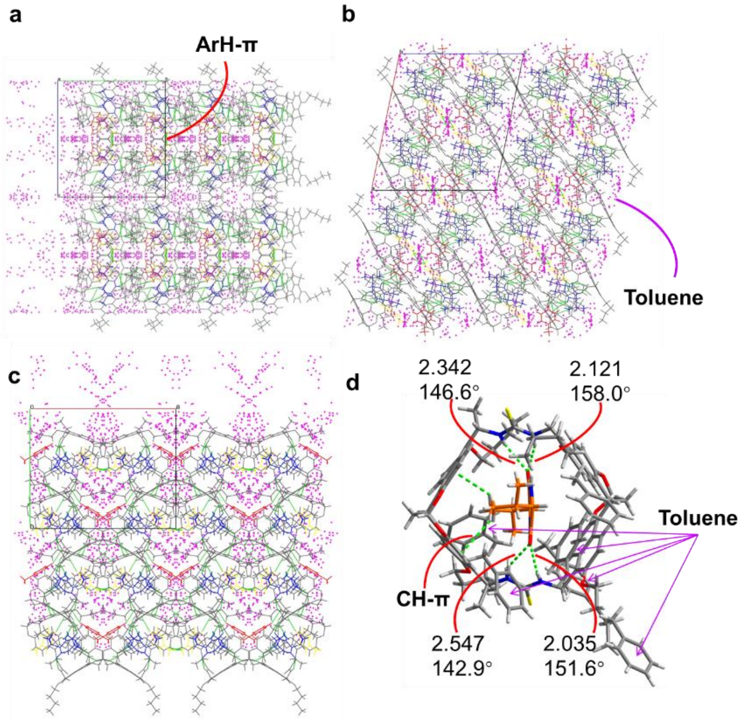

Supplementary Figure 111. Crystal data. Crystal structure of *S,S*-**5c**@*S,S*-**CT2**. Stacking diagrams viewed along **a** a-axis; **b** b-axis; and **c** c-axis; **d** key intermolecular interactions between host and guest. In an *S,S*-**CT2** molecule, *S,S*-**5c** molecule form hydrogen bondings and CH-π with two thiourea and naphthyl groups. The purple asterisks indicate the toluene molecules located within the interval of host-guest complexes. Some parts of the toluenes are depicted as yellow and red lines. The toluene molecules form multiple CH-π (ArH-π) interactions with host in the crystal to achieve crystal stability. Non-covalent interactions are shown as green dashed lines.

2**Toluene@***R,R*-**CT2**

No A or B alerts

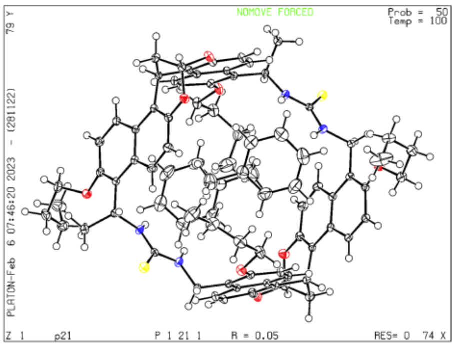

Crystal structure of 2**Toluene@***R,R*-**CT2**

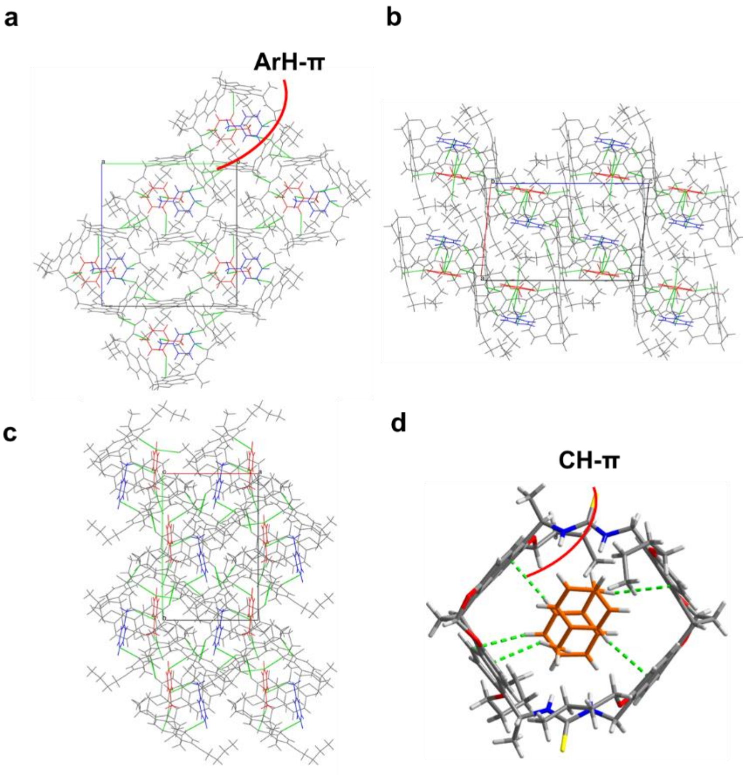

Supplementary Figure 112. Crystal data. Crystal structure of 2**Toluene@***R,R*-**CT2**. Stacking diagrams viewed along **a** a-axis; **b** b-axis; and **c** c-axis; **d** key intermolecular interactions between host and two toluene molecules. In an *R,R*-**CT2** molecule, two toluene molecules, which are depicted as blue and red lines, form CH-π interactions with naphthyl groups. There are multiple CH-π (ArH-π) interactions in the crystal to achieve crystal stability. Non-covalent interactions are shown as green dashed lines.

2.13. Variable Temperature ^{1}H NMR Experiments

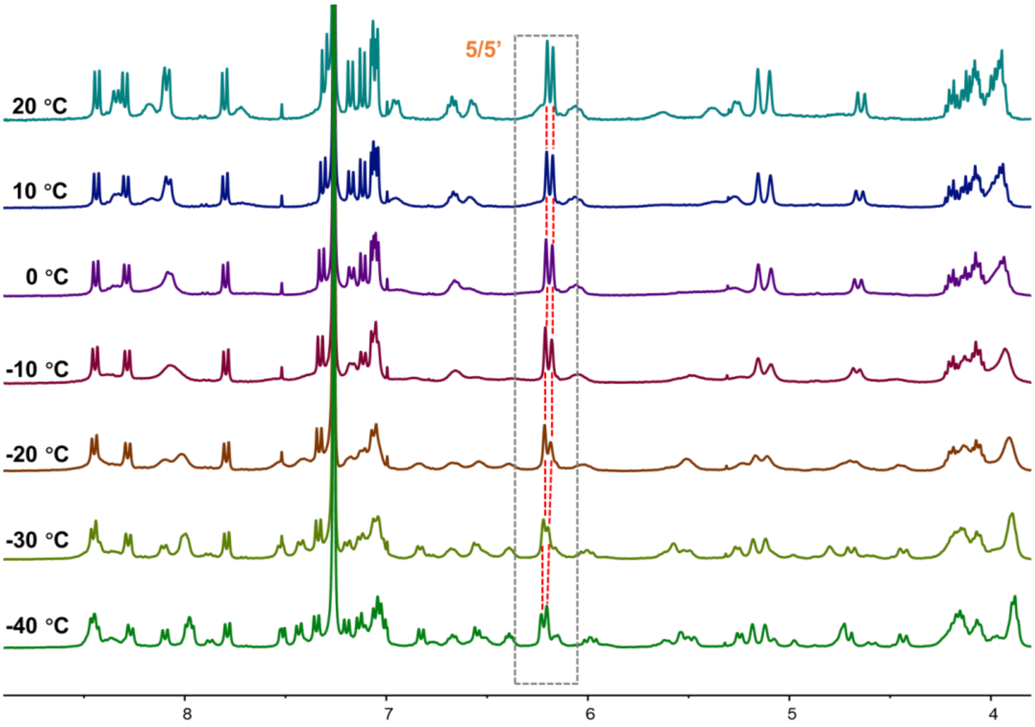

Supplementary Figure 113. Variable temperature experiments. Variable-temperature ^{1}H NMR spectra of *S,S*-**CT1**, the temperature decreased from 20 °C to -40 °C (500 MHz, $CDCl_3$, 0.5 mM, 298 K).

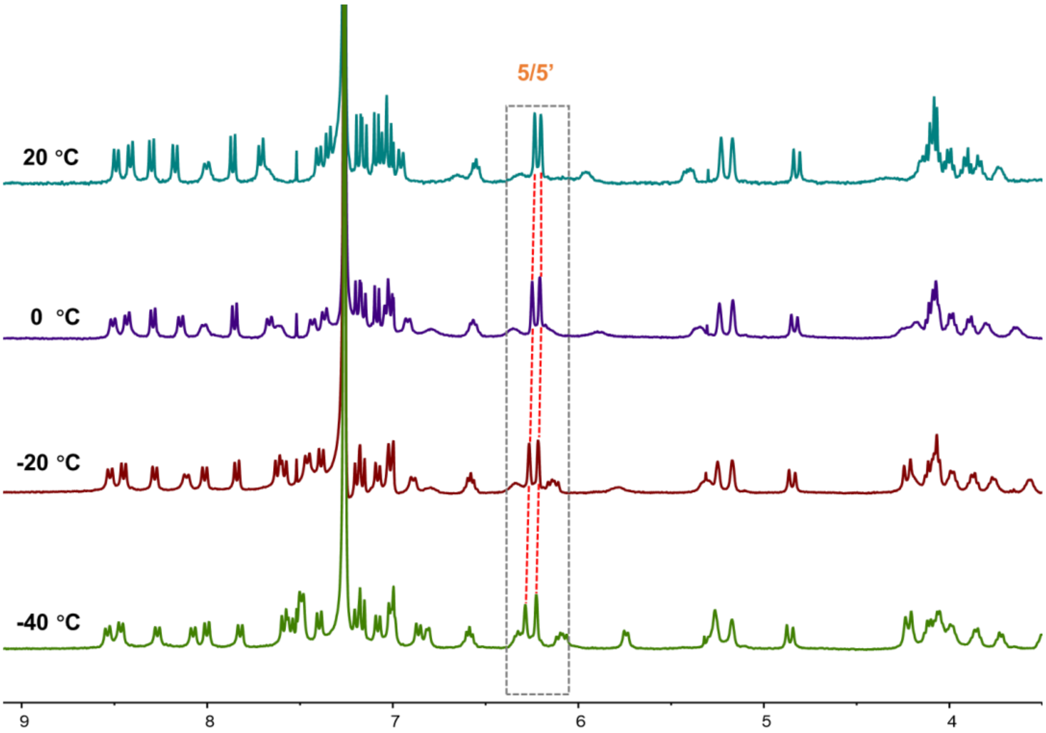

Supplementary Figure 114. Variable temperature experiments. Variable-temperature ^{1}H NMR spectra of *R*-**5a**@*S,S*-**CT2**, the temperature decreased from 20 °C to -40 °C (500 MHz, $CDCl_3$, 0.5 mM, 298 K).

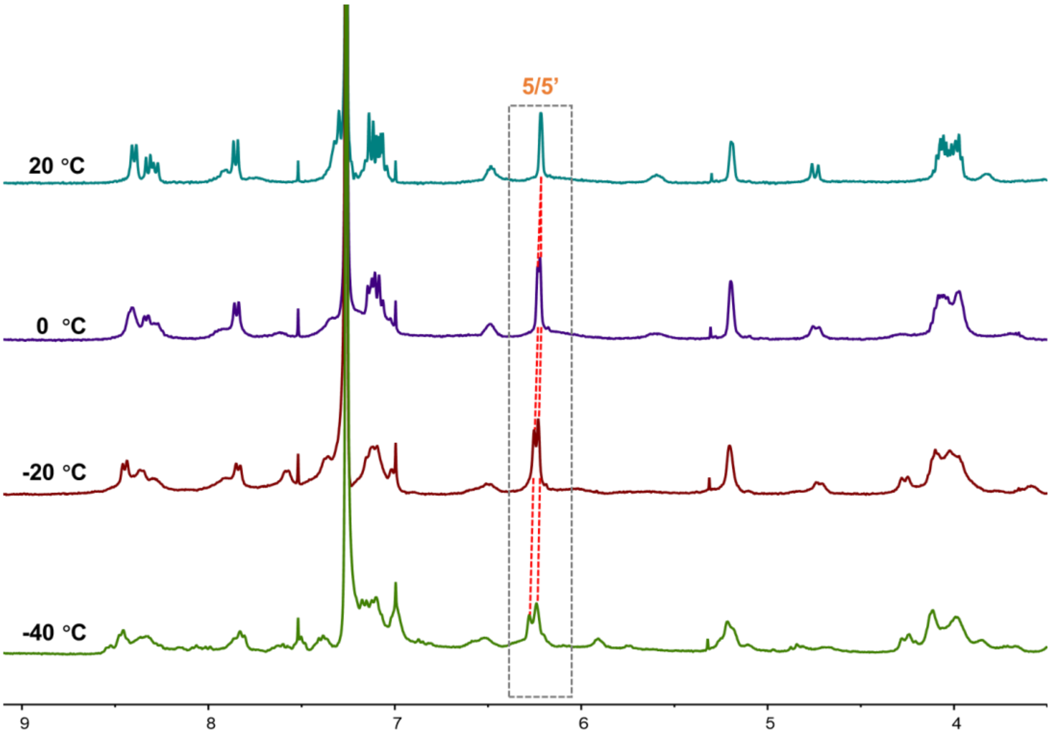

Supplementary Figure 115. Variable temperature experiments. Variable-temperature ^{1}H NMR spectra of *S*-**5a**@*S,S*-**CT2**, the temperature decreased from 20 °C to -40 °C (500 MHz, $CDCl_3$, 0.5 mM, 298 K).

2.14. Circular dichroism (CD), UV-Vis and Fluorescent Spectra of Hosts Interact with Guests (5)

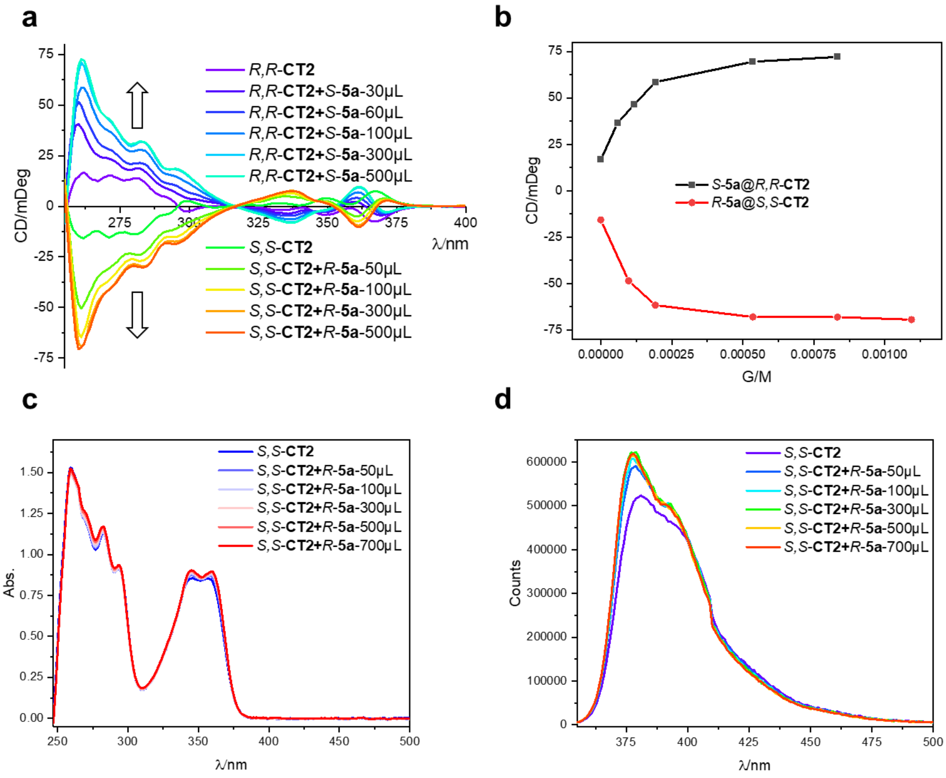

Supplementary Figure 116. Spectral analysis of 5a@CT2 with hetero-chirality by titration exprements. (2.5mL 25 μM host were added by 5 mM guest in dichloroethane to saturated binding, 25 °C). **a** CD spectra; **b** CD intensity at 262 nm in titration experiments; **c** UV-Vis spectra; **d** fluorescent spectra of *R*-**5a**@*S,S*-**CT2**, the spetra of *S*-**5a**@*R,R*-**CT2** are similar.

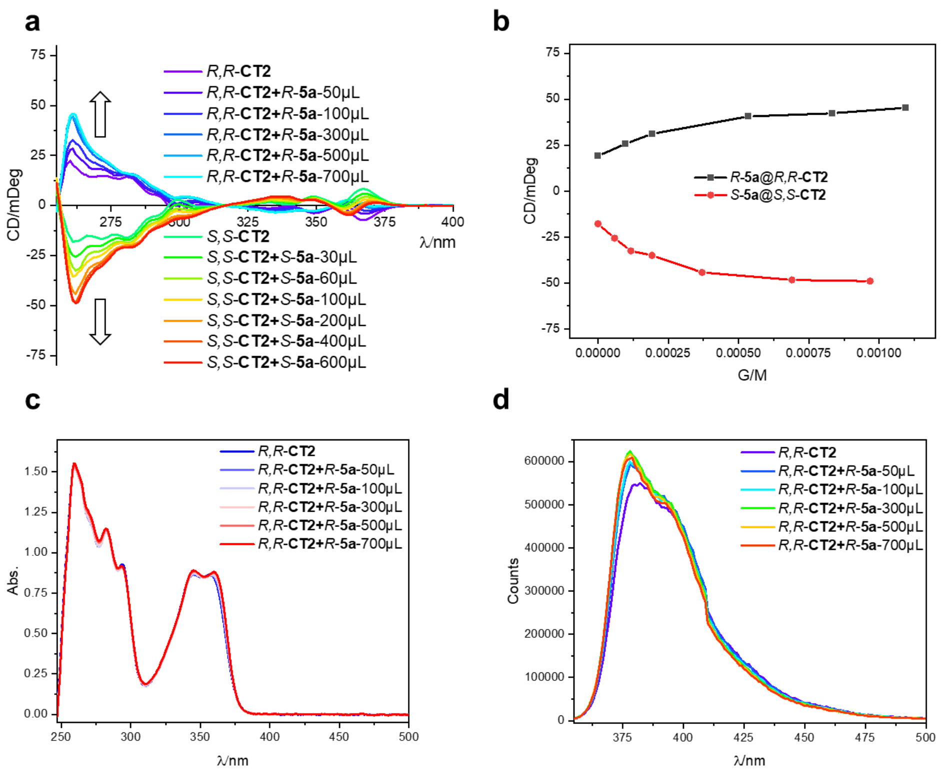

Supplementary Figure 117. Spectral analysis of 5a@CT2 with homo-chirality by titration exprements. (2.5mL 25 μM host were added by 5 mM guest in dichloroethane to saturated binding, 25 °C). **a** CD spectra; **b** CD intensity at 262 nm in titration experiments; **c** UV-Vis spectra; **d** fluorescent spectra of *R*-**5a**@*R*,*R*-**CT2**, the spetra of *S*-**5a**@*S*,*S*-**CT2** are similar.

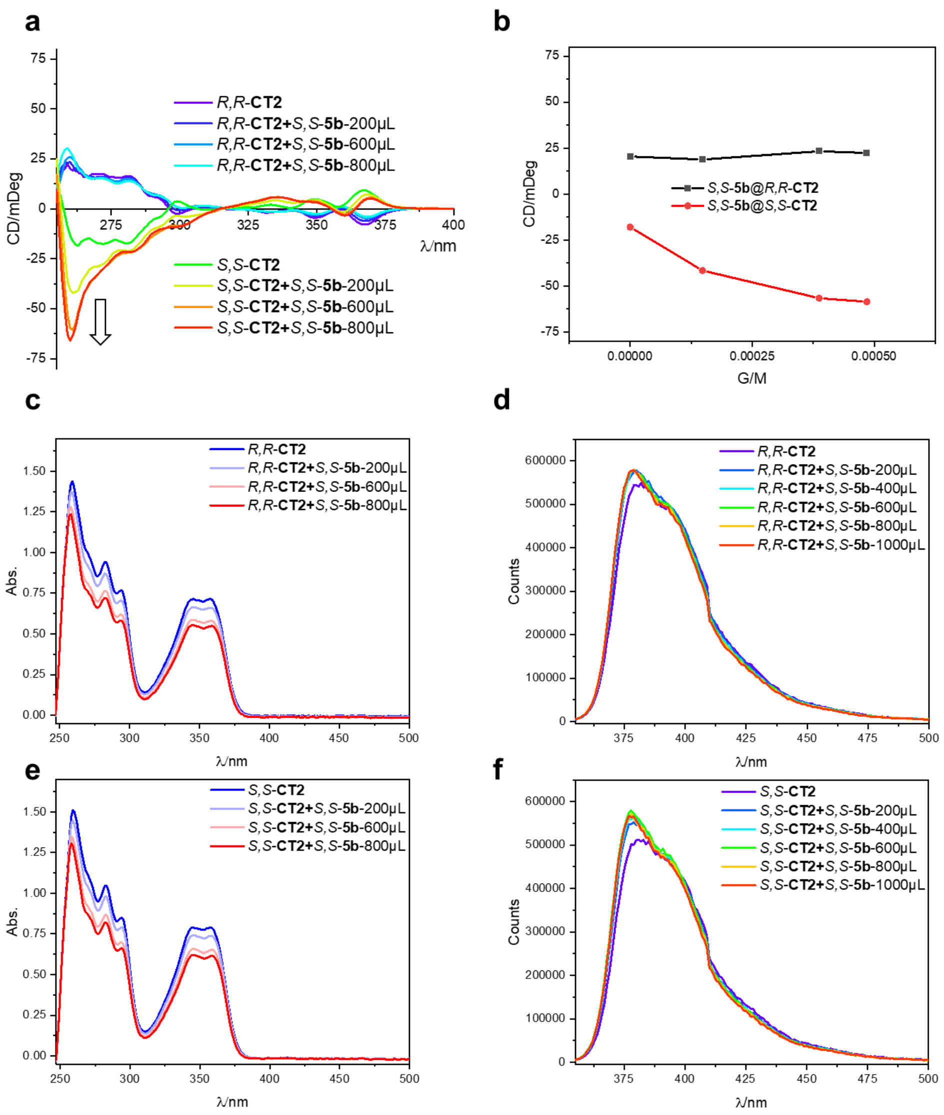

Supplementary Figure 118. Spectral analysis of *S,S*-5b@CT2 by titration exprements. (2.5mL 25 μM host were added by 2 mM guest in dichloroethane to saturated binding, 25 °C). **a** CD spectra; **b** CD intensity at 262 nm in titration experiments; **c** UV-Vis spectra and **d** fluorescent spectra of *S,S*-**5b**@*R,R*-**CT2**; **e** UV-Vis spectra and **f** fluorescent spectra of *S,S*-**5b**@*S,S*-**CT2**.

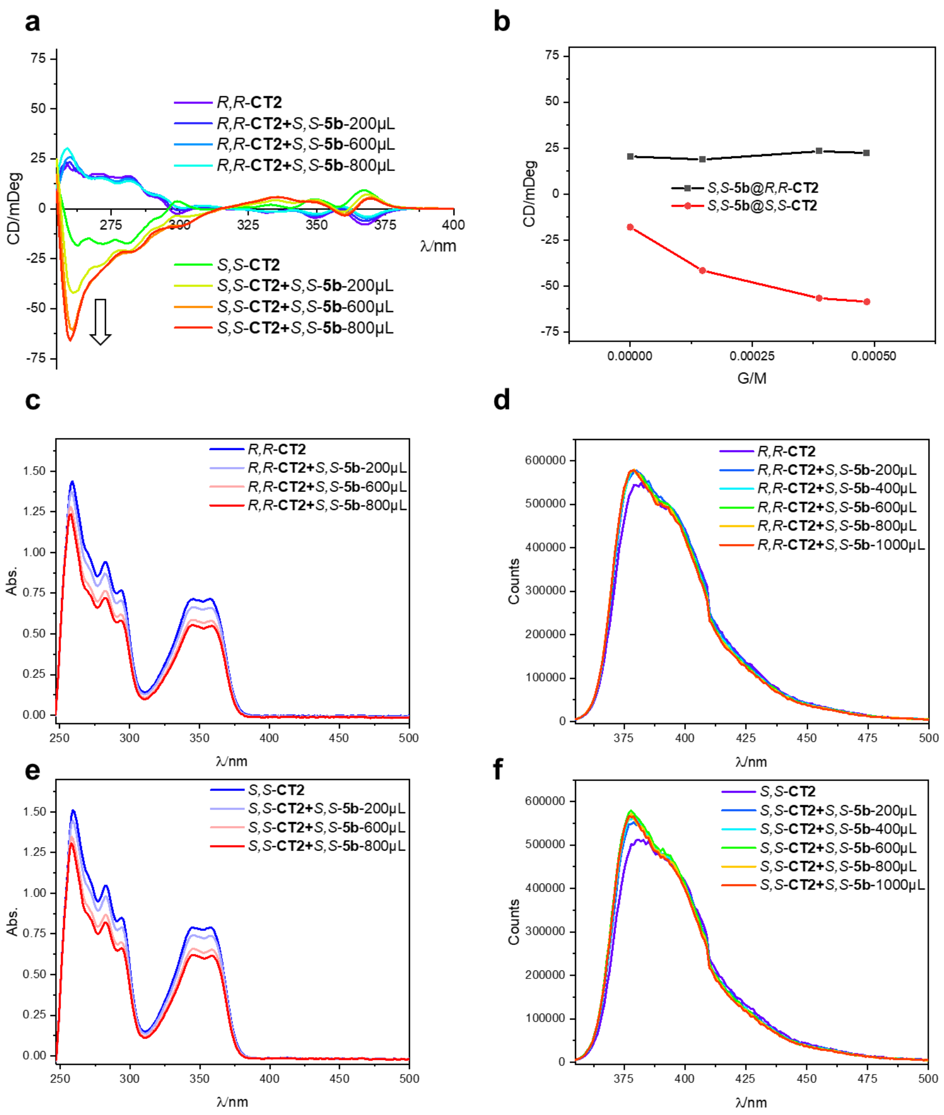

Supplementary Figure 119. Spectral analysis of *S,S*-5c@CT2 by titration exprements. (2.5mL 25 μM host were added by 5 mM guest in dichloroethane to saturated binding, 25 °C). **a** CD spectra; **b** CD intensity at 262 nm in titration experiments; **c** UV-Vis spectra and **d** fluorescent spectra of *S,S*-**5c**@*R,R*-**CT2**; **e** UV-Vis spectra and **f** fluorescent spectra of *S,S*-**5c**@*S,S*-**CT2**.

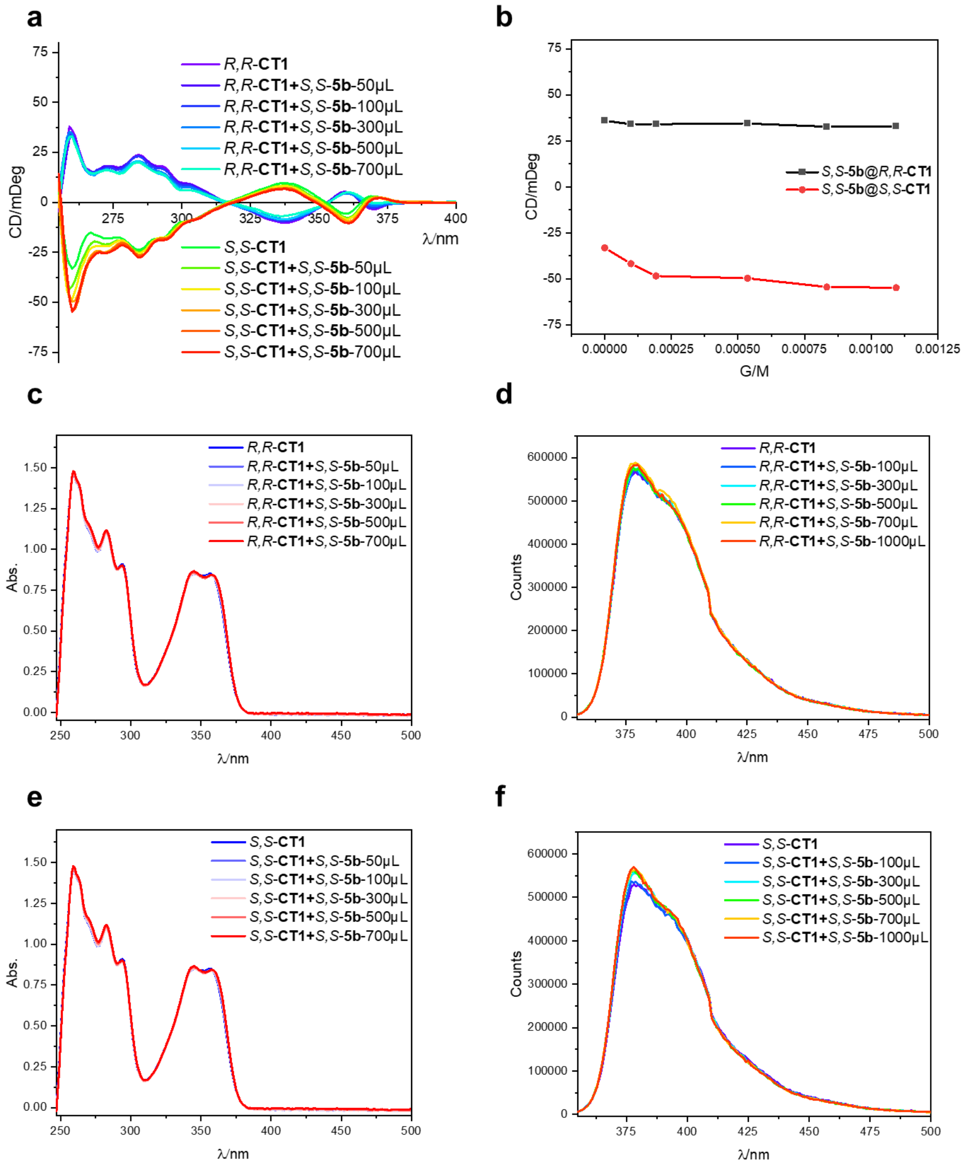

Supplementary Figure 120. Spectral analysis of *S,S*-5b@CT1 by titration exprements. (2.5mL 25 μM host were added by 2 mM guest in dichloroethane to saturated binding, 25 °C). **a** CD spectra; **b** CD intensity at 260 nm in titration experiments; **c** UV-Vis spectra and **d** fluorescent spectra of *S,S*-**5b**@*R,R*-**CT1**; **e** UV-Vis spectra and **f** fluorescent spectra of *S,S*-**5b**@*S,S*-**CT1**.

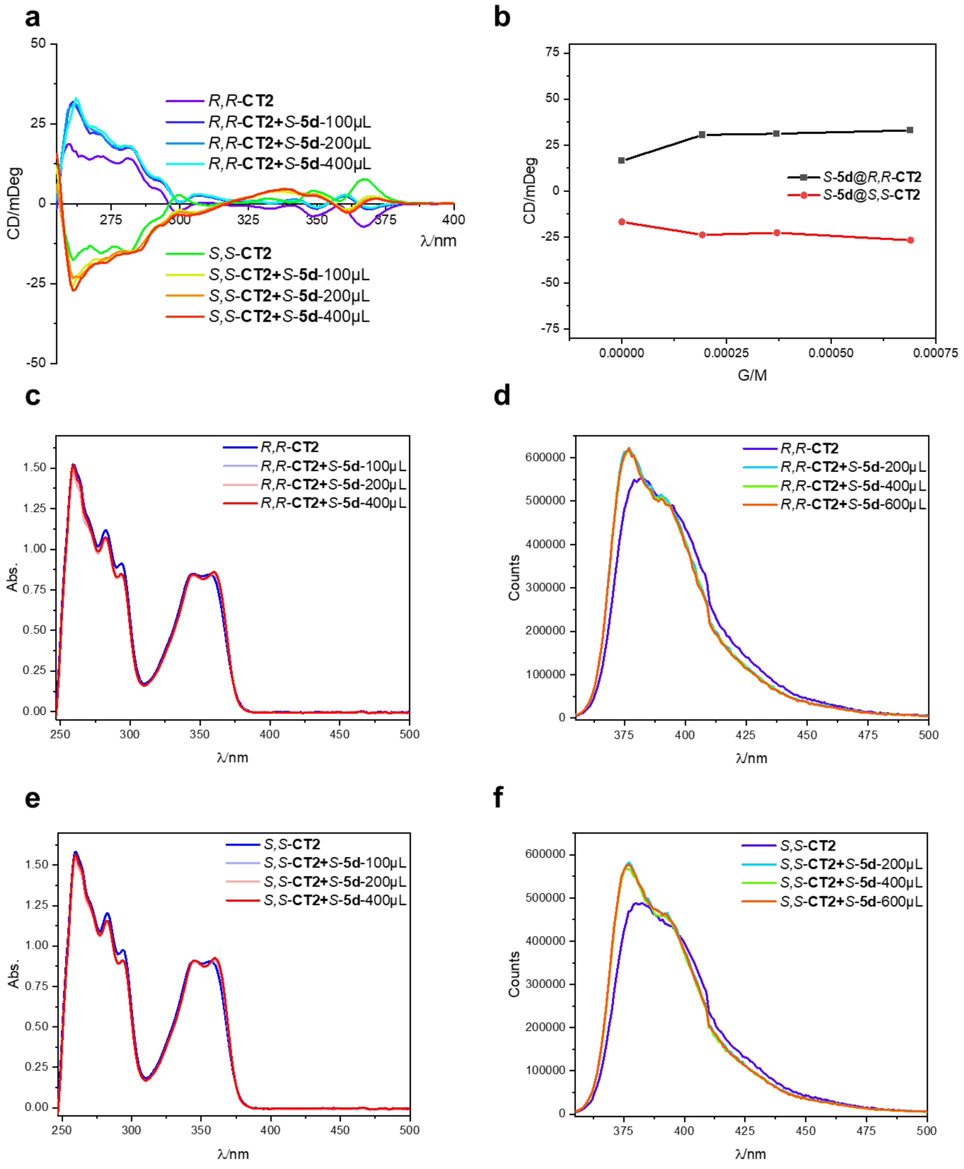

Supplementary Figure 121. Spectral analysis of *S*-5d@CT2 by titration expements. (2.5mL 25 μM host were added by 5 mM guest in dichloroethane to saturated binding, 25 °C). **a** CD spectra; **b** CD intensity at 262 nm in titration experiments; **c** UV-Vis spectra and **d** fluorescent spectra of *S*-**5d**@*R,R*-**CT2**; **e** UV-Vis spectra and **f** fluorescent spectra of *S*-**5d**@*S,S*-**CT2**.

2.15. Computational Data

Geometry optimizations were performed with Gaussian 16 software package,[11] using the M06-2x/def2-svp[12,13] method, by considering the solvent effects (Polarizable-Continuum Model, PCM, Toluene or $CHCl_3$)[6] or spartan 14.0 (Semi-Empirical PM6) without applying any geometry constraints (C1 symmetry). Frequency calculations were then conducted at the same computational level to confirm the nature of all located stationary points and to obtain the thermal correction to Gibbs free energy and enthalpy. Single point energy was calculated with Gaussian 16 software package using the M062x/ma-def2-tzvpp (or ma-def2-tzvp) method,[12] by considering the solvent effects (Solvation Model Based on Solute Electron Density, SMD, Toluene or $CHCl_3$).[14] The total Gibbs free energy of the complex is the addition of single point energy and the thermal correction to Gibbs free energy (ZPE+$\Delta G_{0\rightarrow T}$). Non-covalent interaction (Independent gradient model based on Hirshfeld partition, IGMH) analysis[15] was carried out with Multiwfn 3.8 (dev) program[16,17] and visualized by the VMD 1.9.3 program.[18] (set isovalue = 0.008)

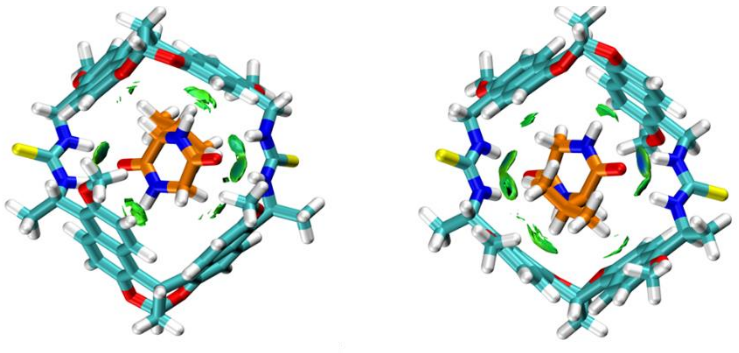

S-**5a**@*S,S*-**CT2** **0** kJ/mol

R-**5a**@*S,S*-**CT2** **-1.59** kJ/mol

Supplementary Figure 122. Non-covalent interaction analysis. Energy minimized structures obtained by DFT (M06-2x/def2-svp) calculations with the PCM solution model in chloroform at 298 K. The single crystal structure of *S*-**5a**@*S,S*-**CT2** and *R*-**5a**@*S,S*-**CT2** were used as an initial guess for self-consistent field (SCF) procedure. The relative Gibbs free energy (ZPE+$\Delta G_{0\rightarrow T}$) is shown (M06-2x/ma-def2-tzvpp with SMD model). *R*-**5a**@*S,S*-**CT2** is more stable than *S*-**5a**@*S,S*-**CT2** by -1.59 kJ/mol, which qualitatively agrees with the results of ^{1}H NMR titration.

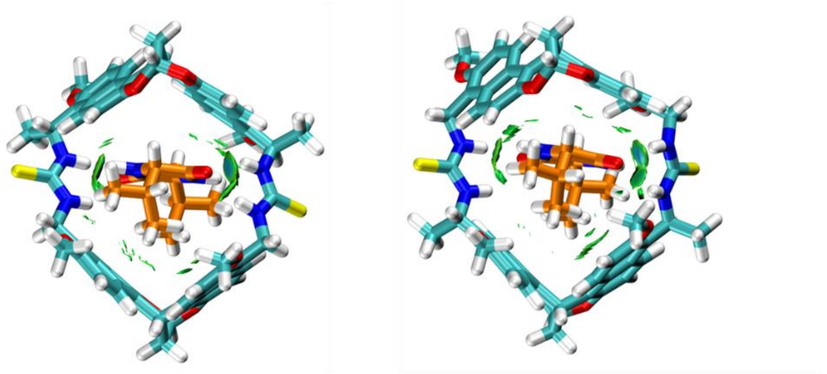

S,S-**5c**@*R,R*-**CT2** **0** kJ/mol

S,S-**5c**@*S,S*-**CT2** **-3.33** kJ/mol

Supplementary Figure 123. Non-covalent interaction analysis. Energy minimized structures obtained by DFT (M06-2x/def2-svp) calculations with the PCM solution model in chloroform at 298 K. The relative Gibbs free energy (ZPE+$\Delta G_{0\rightarrow T}$) is shown (M06-2x/ma-def2-tzvpp with SMD model). *S,S*-**5c**@*S,S*-**CT2** is more stable than *S,S*-**5c**@*R,R*-**CT2** by -3.33 kJ/mol, which qualitatively agrees with the results of ^{1}H NMR titration.

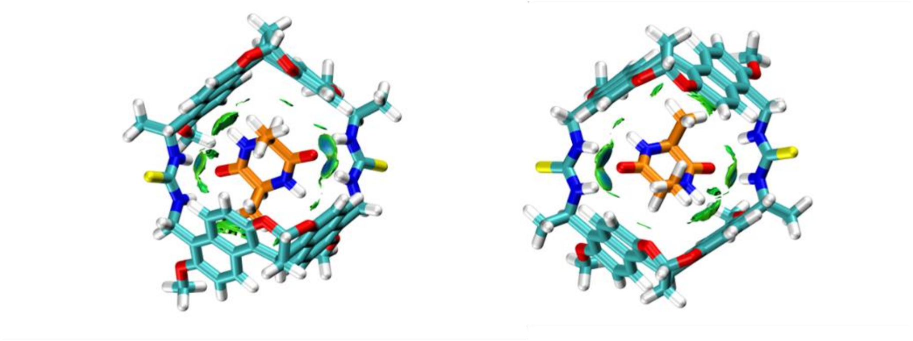

S,S-**5b**@*R,R*-**CT2**
0 kJ/mol

S,S-**5b**@*S,S*-**CT2**
-2.51 kJ/mol

Supplementary Figure 124. Non-covalent interaction analysis. Energy minimized structures obtained by DFT (M06-2x/def2-svp) calculations with the PCM solution model in chloroform at 298 K. The relative Gibbs free energy (ZPE+$\Delta G_{0\rightarrow T}$) is shown (M06-2x/ma-def2-tzvpp with SMD model). *S,S*-**5b**@*S,S*-**CT2** is more stable than *S,S*-**5b**@*R,R*-**CT2** by -2.51 kJ/mol, which qualitatively agrees with the results of ^{1}H NMR titration.

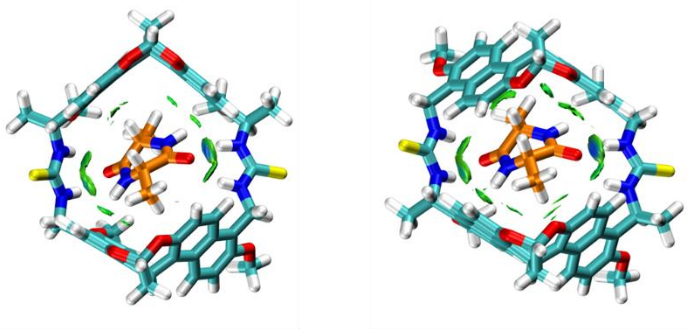

S,S-**5b**@*R,R*-**CT1**
0 kJ/mol

S,S-**5b**@*S,S*-**CT1**
-6.77 kJ/mol

Supplementary Figure 125. Non-covalent interaction analysis. Energy minimized structures obtained by DFT (M06-2x/def2-svp) calculations with the PCM solution model in chloroform at 298 K. The relative Gibbs free energy (ZPE+$\Delta G_{0\rightarrow T}$) is shown(M06-2x/ma-def2-tzvpp with SMD model). *S,S*-**5b**@*S,S*-**CT1** is more stable than *S,S*-**5b**@*R,R*-**CT1** by -6.77 kJ/mol, which qualitatively agrees with the results of ^{1}H NMR titration.

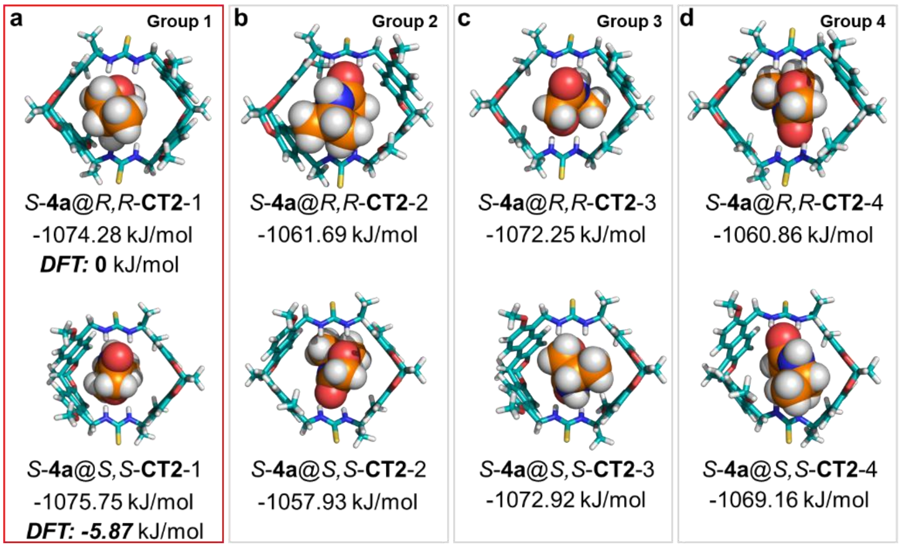

Supplementary Figure 126. Binding modes of *S*-4a@CT2. According to the opposite binding mode obtained in chiral guests **5** within chiral hosts, four possible binding modes of *S*-**4a**@*R*,*R*-**CT2** were used as initial guess for theoretical calculation, including **a** group 1; **b** group 2; **c** group 3; **d** group 4. Accordingly, the *S,S*-**CT2** accommodate the *S*-**4a** with similar space and binding sites in this binding mode, four possible binding modes of *S*-**4a**@*S,S*-**CT2** were given by flipping *S*-**4a** within cavity as initial guess. Energy minimized structures and energies were obtained by Spartan 14.0 (Semi-Empirical PM6). Group 1 (marked with red box) was considered as the most possible binding mode for both binding strutures with the lowest energy. The host-guest binding structures in group 1 as initial guess were used for more precise theoretical calculations by DFT calculation (M06-2x/def2-svp) calculations with the PCM model in toluene at 298 K, the relative energies are shown (M06-2x/ma-def2-tzvp with SMD model), the stability of *S*-**4a**@*S,S*-**CT2**-1 is higher than that of *S*-**4a**@*R,R*-**CT2**-1 with a lower free energy of -5.87 kJ/mol, which is in qualitative agreement with the ^{1}H NMR titration result.

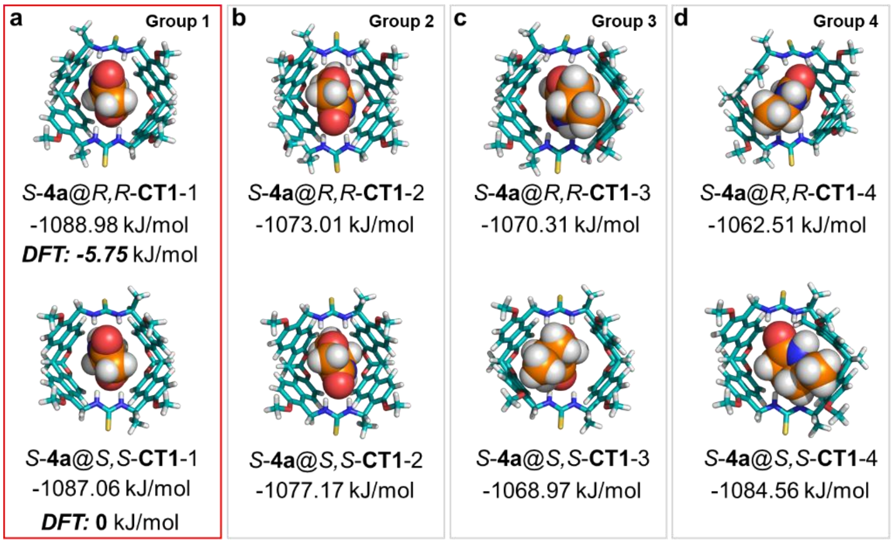

Supplementary Figure 127. Binding modes of *S*-4a@CT1. By the same way used in *S*-**4a**@*R*,*R*-**CT2**, four possible binding modes of *S*-**4a@CT1** were used as initial guess for theoretical calculation, including **a** group 1; **b** group 2; **c** group 3; **d** group 4. The binding mode of *S*-**4a@CT1** was determined as group 1 (marked with red box) adopting a standing orientation. the stability of *S*-**4a**@*R*,*R*-**CT1**-1 is higher than that of *S*-**4a**@*S*,*S*-**CT1**-1, with a lower free energy of -5.75 kJ/mol, which was in qualitative agreement with the ^{1}H NMR titration result.

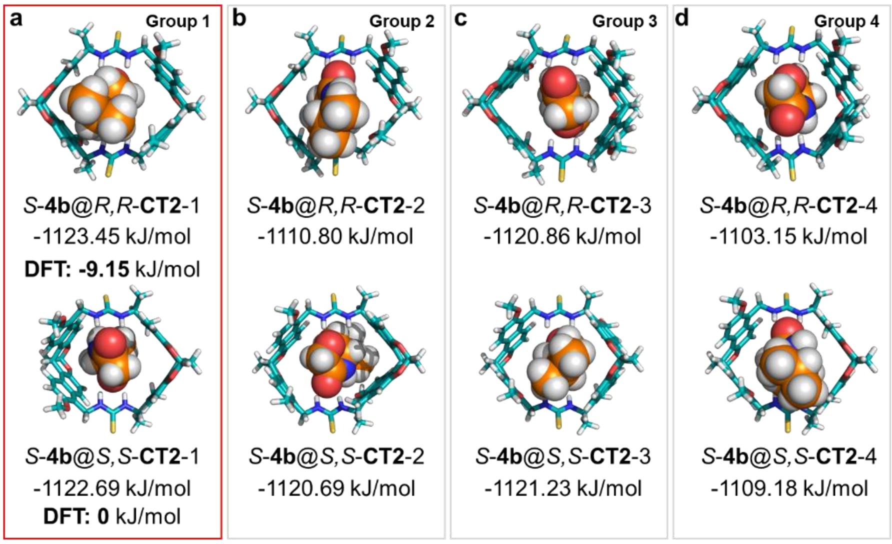

Supplementary Figure 128. Binding modes of *S*-4b@CT2. By the same way used in *S*-**4a**@*R*,*R*-**CT2**, four possible binding modes of *S*-**4b@CT2** were used as initial guess for theoretical calculation, including **a** group 1; **b** group 2; **c** group 3; **d** group 4. The binding mode of *S*-**4b@CT2** was determined as group 1 (marked with red box) adopting a standing orientation as *S*-**4a@CT2**. The stability of *S*-**4b**@*R*,*R*-**CT2**-1 is higher than that of *S*-**4b**@*S*,*S*-**CT2**-1, with a lower free energy of -9.15 kJ/mol, which was in qualitative agreement with the ^{1}H NMR titration result.

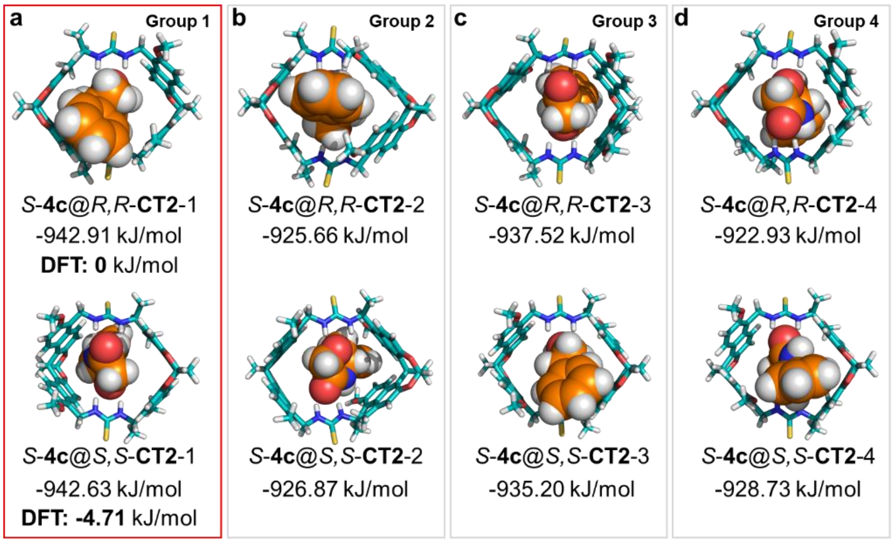

Supplementary Figure 129. Binding modes of *S*-**4c@CT2**. By the same way used in *S*-**4a**@*R,R*-**CT2**, four possible binding modes of *S*-**4c@CT2** were used as initial guess for theoretical calculation, including **a** group 1; **b** group 2; **c** group 3; **d** group 4. The binding mode of *S*-**4c@CT2** was determined as Group 1 (marked with red box) adopting a standing orientation as *S*-**4a@CT2**. The stability of *S*-**4c**@*S,S*-**CT2**-1 is higher than that of *S*-**4c**@*R,R*-**CT2**-1, with a lower free energy of -4.71 kJ/mol, which was in qualitative agreement with the ^{1}H NMR titration result.

Supplementary Table 2: Thermodynamic parameters of all calculated structures (298 K) Gibbs free energy (Δ***G*** = ZPE + $G_{0\to T}$, kJ/mol) and enthalpy (Δ***H*** = ZPE + $H_{0\to T}$, kJ/mol) are obtained after thermal correction. ΔΔ***G*** represent the differences of Gibbs free energy of chiral recognition, ΔΔ***H*** represent the differences of enthalpy of chiral recognition, ***-T*ΔΔ*S*** *(*kJ/mol*)* were calculated by thermodynamic formula (ΔΔ***G*** = ΔΔ***H*** - ***T*ΔΔ*S***).

	ΔG	*ΔΔG*	*ΔH*	*ΔΔH*	*-TΔΔS*
R-**5a**@*S,S*-**CT2**	-11530210.56	**-1.59**	-11529750.38	**-2.91**	**1.32**
S-**5a**@*S,S*-**CT2**	-11530208.96		-11529747.48		
S,S-**5b**@*S,S*-**CT1**	-11427093.58	**-6.77**	-11426634.81	**-8.19**	**1.42**
S,S-**5b**@*R,R*-**CT1**	-11427086.80		-11426626.62		
S,S-**5b**@*S,S*-**CT2**	-11427086.01	**-2.51**	-11426629.47	**-1.47**	**-1.04**
S,S-**5b**@*R,R*-**CT2**	-11427083.50		-11426627.99		
S,S-**5c**@*S,S*-**CT2**	-11839629.10	**-3.33**	-11839154.18	**-5.58**	**2.25**
S,S-**5c**@*R,R*-**CT2**	-11839625.78		-11839148.61		
S-**4a**@*S,S*-**CT2**	-11181530.78	**-5.87**	-11181085.44	**-4.21**	**-1.66**
S-**4a**@*R,R*-**CT2**	-11181524.91		-11181081.22		
S-**4a**@*R,R*-**CT1**	-11181552.70	**-5.75**	-11181107.18	**-7.30**	**1.55**
S-**4a**@*S,S*-**CT1**	-11181546.95		-11181099.87		
S-**4b**@*R,R*-**CT2**	-11387809.55	**-9.15**	-11387343.96	**-5.36**	**-3.79**
S-**4b**@*S,S*-**CT2**	-11387800.40		-11387338.59		
S-**4c**@*S,S*-**CT2**	-11684724.80	**-4.71**	-11684261.74	**-4.70**	**-0.01**
S-**4c**@*R,R*-**CT2**	-11684729.51		-11684257.03		

2.16. Circular dichroism (CD), UV-Vis and Fluorescent Spectra of Hosts Interact with Guests (4)

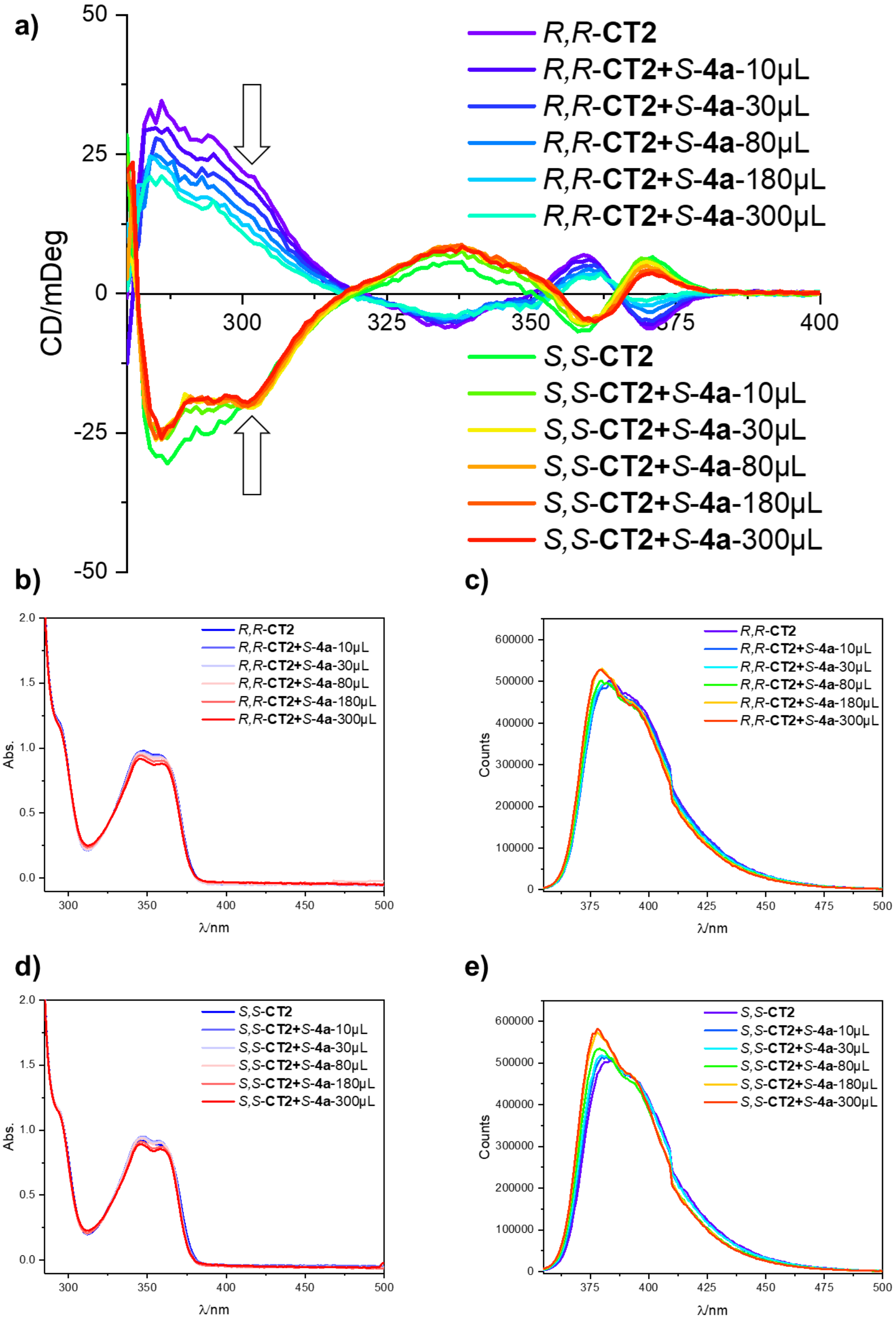

Supplementary Figure 130. Spectral analysis of *S*-**4a@CT2** by titration exprements. (2.5mL 25 μM host were added by 800 mM guest in toluene to saturated binding, 25 °C). a) CD spectra; b) UV-Vis spectra and c) fluorescent spectra of *S*-**4a**@*R,R*-**CT2**; d) UV-Vis spectra and e) fluorescent spectra of *S*-**4a**@*S,S*-**CT2**.

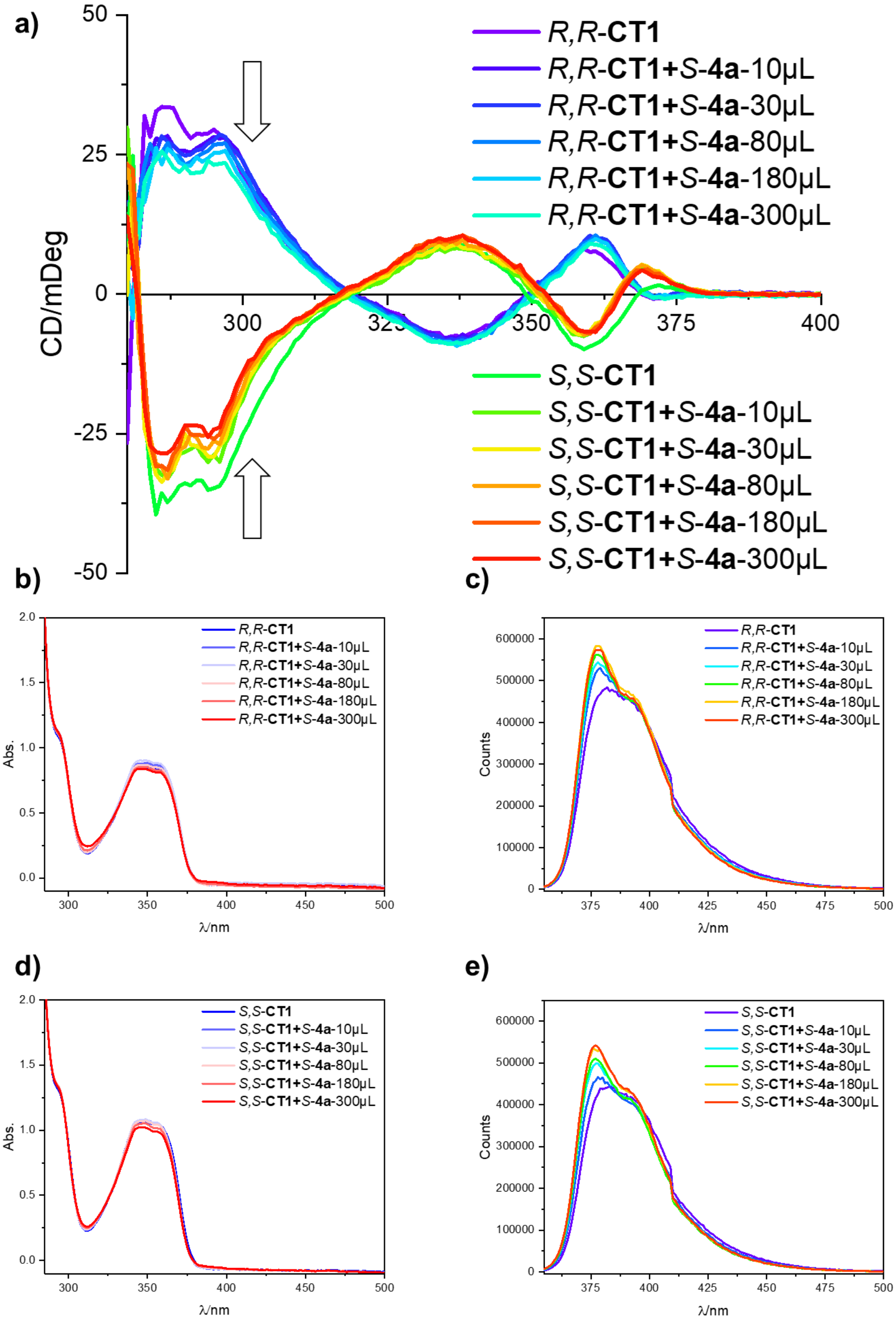

Supplementary Figure 131. Spectral analysis of *S*-**4a@CT1** by titration exprements (2.5mL 25 μM host were added by 800 mM guest in toluene to saturated binding, 25 °C). a) CD spectra; b) UV-Vis spectra and c) fluorescent spectra of *S*-**4a**@*R*,*R*-**CT1**; d) UV-Vis spectra and e) fluorescent spectra of *S*-**4a**@*S*,*S*-**CT1**.

3. Supplementary References

1. Shorthill, B. J., Avetta, C. T., & Glass, T. E. Shape-selective sensing of lipids in aqueous solution by a designed fluorescent molecular tube. *J. Am. Chem. Soc.* **126**, 12732-12733 (2004).
2. Hariharan, P. C., & Pople, J. A. Accuracy of AH nequilibrium geometries by single determinant molecular orbital theory. *Mol. Phys.* **27**, 209-214 (1974).
3. Petersson, G. A., Bennett, A., Tensfeldt, T. G., Laham, Al, M. A., Shirley, W. A., & Mantzaris, J. A complete basis set model chemistry. I. the total energies of closed-shell atoms and hydrides of the first-row elements. *J. Chem. Phys.* **89**, 2193-2218 (1988).
4. Grimme, S., Antony, J., Ehrlich, S., & Krieg, H. A Consistent and accurate ab Initio parametrization of density functional dispersion correction (DFT-D) for the 94 elements H-Pu. *J. Chem. Phys.* **132**, 154104 (2010).
5. Grimme, S., Ehrlich, S., & Goerigk, L. Effect of the damping function in dispersion corrected density functional theory. *J. Comput. Chem.* **32**, 1456-1465 (2011).
6. Cancès E., Mennucci B., & Tomasi J. A new integral equation formalism for the polarizable continuum model: Theoretical background and applications to isotropic and anisotropic dielectrics. *J. Chem. Phys.* **107**, 3032-3041 (1997).
7. Mennucci B., & Tomasi J. Continuum solvation models: A new approach to the problem of solute's charge distribution and cavity boundaries. *J. Chem. Phys.* **106**, 5151-5158 (1997).
8. Guo D.-S., Uzunova V. D., Su X., Liu Y. & Nau W. M. Operational calixarene-based fluorescent sensing systems for choline and acetylcholine and their application to enzymatic reactions. *Chem. Sci.* **2**, 1722-1734 (2011).
9. Sheldrick G. M. Crystal structure refinement with SHELXL. *Acta Cryst.* **71**, 3-8 (2015).
10. Dolomanov O. V., Bourhis L. J., Gildea R. J., Howard J. A. K., & Puschmann H. OLEX2: a complete structure solution, refinement and analysis program. *J. Appl. Cryst.* **42**, 339-341 (2009).
11. Gaussian 16, Revision A.03, M. J. Frisch, G. W. Trucks, H. B. Schlegel, G. E. Scuseria, M. A. Robb, J. R. Cheeseman, G. Scalmani, V. Barone, G. A. Petersson, H. Nakatsuji, X. Li, M. Caricato, A. V. *Marenich*, J. Bloino, B. G. Janesko, R. Gomperts, B. Mennucci, H. P. Hratchian, J. V. Ortiz, A. F. Izmaylov, J. L. Sonnenberg, D. Williams-Young, F. Ding, F. Lipparini, F. Egidi, J. Goings, B. Peng, A. Petrone, T. Henderson, D. Ranasinghe, V. G. Zakrzewski, J. Gao, N. Rega, G. Zheng, W. Liang, M. Hada, M. Ehara, K. Toyota, R. Fukuda, J. Hasegawa, M. Ishida, T. Nakajima, Y. Honda, O. Kitao, H. Nakai, T. Vreven, K. Throssell, J. A. Montgomery, Jr., J. E. Peralta, F. Ogliaro, M. J. Bearpark, J. J. Heyd, E. N. Brothers, K. N. Kudin, V. N. Staroverov, T. A. Keith, R. Kobayashi, J. Normand, K. Raghavachari, A. P. Rendell, J. C. Burant, S. S. Iyengar, J. Tomasi, M. Cossi, J.

M. Millam, M. Klene, C. Adamo, R. Cammi, J. W. Ochterski, R. L. Martin, K. Morokuma, O. Farkas, J. B. Foresman, and D. J. Fox, Gaussian, Inc., Wallingford CT, 2016.

12. Zhao Y. & Truhlar D. G. *Theor. Chem. Account* The M06 suite of density functionals for main group thermochemistry, thermochemical kinetics, noncovalent interactions, excited states, and transition elements: two new functionals and systematic testing of four M06-class functionals and 12 other functionals. **120**, 215-241 (2008).
13. Weigend F., & Ahlrichs R. Balanced basis sets of split valence, triple zeta valence and quadruple zeta valence quality for H to Rn: Design and assessment of accuracy. *Phys. Chem. Chem. Phys.* **7**, 3297-3305 (2005).
14. Marenich, A. V., Cramer, C. J., & Truhlar, D. G. Universal solvation model based on solute electron density and on a continuum model of the solvent defined by the bulk dielectric constant and atomic surface tensions. *J. Phys. Chem. B* **113**, 6378–6396 (2009).
15. Lu, T., & Chen, Q. Independent gradient model based on Hirshfeld partition: A new method for visual study of interactions in chemical systems. *J. Comput. Chem.* **43**, 539-555 (2022).
16. Lu T., & Chen F., Multiwfn: A multifunctional wavefunction analyzer. *J. Comput. Chem.* **33***,* 580−592 (2012)..
17. Zhang J., & Lu T. Efficient evaluation of electrostatic potential with computerized optimized code. *Phys. Chem. Chem. Phys. 23*, 20323-20328 (2021).
18. Humphrey W., Dalke A., Schulten K. VMD: Visual molecular dynamics. *J. Mol. Graph. Model.* **14***,* 33-38 (1996).
